# Vinclozolin induced epigenetic transgenerational inheritance of pathologies and sperm epimutation biomarkers for specific diseases

**DOI:** 10.1371/journal.pone.0202662

**Published:** 2018-08-29

**Authors:** Eric Nilsson, Stephanie E. King, Margaux McBirney, Deepika Kubsad, Michelle Pappalardo, Daniel Beck, Ingrid Sadler-Riggleman, Michael K. Skinner

**Affiliations:** Center for Reproductive Biology, School of Biological Sciences, Washington State University, Pullman, WA, United States of America; INIA, SPAIN

## Abstract

Exposure to vinclozolin has been shown to induce the epigenetic transgenerational inheritance of increased susceptibility to disease, and to induce transgenerational changes to the epigenome. In the current study, gestating F0 generation rats were exposed to vinclozolin, and the subsequent F1, F2 and transgenerational F3 generations were evaluated for diseases and pathologies. F1 and F2 generation rats exhibited few abnormalities. However, F3 generation rats showed transgenerational increases in testis, prostate, and kidney disease, changes in the age of puberty onset in males, and an increased obesity rate in females. Overall there was an increase in the rate of animals with disease, and in the incidence of animals with multiple diseases. The objective of the current study was to analyze the sperm epigenome of F3 generation rats with specific abnormalities and compare them to rats without those abnormalities, in an effort to find epigenetic biomarkers of transgenerational disease. Unique signatures of differential DNA methylation regions (DMRs) in sperm were found that associated with testis disease, prostate disease and kidney disease. Confounding factors identified were the presence of multiple diseases in the analysis and the limited number of animals without disease. These results further our understanding of the mechanisms governing epigenetic transgenerational inheritance, and may lead in the future to the use of epigenetic biomarkers that will help predict an individual’s susceptibility for specific diseases.

## Introduction

Epigenetic transgenerational inheritance involves the germline transmission of epigenetic information and phenotypic change across generations in the absence of any continued direct environmental exposure or genetic manipulation [1, 2]. Epigenetics is defined as molecular factors around the DNA that regulate genome activity independent of DNA sequence and that are mitotically stable [3]. Epigenetic factors include DNA methylation, histone modifications, non-coding RNA (ncRNA), RNA methylation and chromatin structure [2]. Examples of epigenetic transgenerational inheritance were observed in studies in which pregnant F0 generation female rats were exposed to the fungicide vinclozolin or insecticide DDT (dichlorodiphenyltrichloroethane) during the period of gonadal sex determination for the developing fetus [4, 5]. This treatment regimen directly exposed the F0 generation female and the developing F1 generation fetus to the toxicant, as well as exposing the developing F1 generation germ cells that will become sperm and eggs that produce the F2 generation. The subsequent F3 generation rats (great-grand-offspring) received no direct toxicant exposure so are the first transgenerational generation. The F3 generation rats showed increased rates of obesity, testis disease, polycystic ovary disease and kidney disease compared to controls. They also had epigenetic changes in sperm comprised of differential DNA methylated regions (DMRs). These pathologies and epigenetic changes in non-exposed F3 generation rats involve the non-genetic inheritance phenomenon termed epigenetic transgenerational inheritance [4]. Those changes seen in the F1 and F2 generations are from direct toxicant exposure and/or induced epigenetic change, so are examples of multigenerational inheritance [6]. The current study is focused on the transgenerational F3 generation disease biomarkers.

Epigenetic transgenerational inheritance of increased disease rates and susceptibility has been demonstrated after ancestral exposure to a variety of toxicants in addition to vinclozolin or DDT [4, 7]. These toxicants include the insecticide permethrin and the insect repellant DEET (N,N-diethyl-meta-toluamide) [8], the plastic compounds bisphenol A (BPA) and phthalates [9–11], jet fuel JP8 [12], the industrial pollutants dioxin (TCDD) [13–15], benzo(a)pyrene [16] and methylmercury [17], the pesticide methoxychlor [18], the estrogenic compound genistein [19], and the herbicide atrazine [20]. In addition to environmental toxicants, nutrition is one of the primary environmental factors able to promote the epigenetic transgenerational inheritance of pathologies [2]. The epigenetic transgenerational inheritance phenomenon has been described in plants [21], flies [22], worms [23], fish [24], birds [19], rodents [4], pigs [25], and humans [26]. Therefore, the epigenetic transgenerational inheritance phenomenon is highly conserved and this non-genetic form of inheritance is a critical method by which the environment impacts biology.

Vinclozolin is an agricultural fungicide known to have anti-androgenic endocrine disrupting activity [27]. Exposure to vinclozolin has been shown to induce the epigenetic transgenerational inheritance of increased susceptibility to disease, and transgenerational changes to the epigenome [4]. In rats, ancestral vinclozolin exposure has resulted in transgenerational increases in testis disease and associated decreases in sperm number and motility [4, 28], as well as an increased prostate disease, kidney disease, ovarian disease and tumor incidence [29–32]. These increases in disease susceptibility have been associated with transgenerational epigenome changes (i.e. DMRs or epimutations) in the promoter regions of sperm DNA [33], and altered small non-coding RNA (ncRNA) in the sperm [34]. Since the germline (e.g. sperm) transmits an altered epigenome to the early developing embryos and embryonic stem cells, all subsequent tissues derived from the embryonic stem cells will also have epigenetic alterations [2]. Previous studies have demonstrated the transgenerational changes to transcriptomes and epigenomes in somatic cells, including transgenerational DMRs in testis Sertoli cells, primordial germ cells [35, 36], and ovarian granulosa cells [32]. Transgenerational increases in disease incidence and associated changes in DNA methylation following ancestral exposure to vinclozolin have also been correlated to transgenerational changes in gene expression in the affected tissues [30, 32, 35, 37]. In mice, ancestral exposure to vinclozolin has been shown to promote epigenetic transgenerational inheritance of disease and induce transgenerational changes in sperm DNA methylation [38, 39]. In a recent study in mosquitoes, exposure to vinclozolin has resulted in a transgenerational increase in the phenotypic variation seen in subsequent generations as it relates to cold hardiness of eggs and larvae, which is accompanied by an epigenetic transgenerational change in global DNA methylation levels [40]. Previous studies indicate vinclozolin exposure is a robust inducer of the epigenetic transgenerational inheritance of disease.

Two recent studies have suggested that sperm DNA may contain definable patterns of epigenetic changes that can serve as epigenetic biomarkers for specific environmental exposures and disease susceptibility. The first study used sperm from human males of approximately 30 years of age that had received chemotherapy exposure during puberty to treat bone cancer and a control population with no chemotherapy exposure [41]. A sperm DMR signature was observed providing a potential biomarker or diagnostic for earlier life chemotherapy exposure. How this may influence subsequent offspring remains to be established. The second study involved gestating F0 generation female rats exposed to the herbicide atrazine [20]. Analyses of DMR associations with specific diseases in the F3 generation atrazine lineage males identified sets of DMRs (epimutations) that were specifically associated with individuals with either testis or metabolic disease. These studies provide the proof of concept that transgenerational sperm DMRs may provide biomarkers for specific diseases. Several studies have also demonstrated the use of sperm DMRs for the identification of human male infertility [42, 43]. Therefore, the major objectives of the current study was to identify potential epigenetic biomarkers for disease.

In the current study, pregnant F0 generation rats were exposed to vinclozolin and the subsequent F1, F2 and transgenerational F3 generations were evaluated for disease and pathologies. In previous studies the exposure effects on F2 generation animal pathology have not been well characterized and so are analyzed here. The study provided a population of F3 generation animals with and without specific transgenerational abnormalities and disease conditions. Since the F1 and F2 generation animals involved direct exposure toxicity, the study for epigenetic analysis was focused on the transgenerational F3 generation. The objective of the current study was to analyze the sperm epigenome of F3 generation rats with specific abnormalities, such as prostate disease, and compare them to rats without those abnormalities in an effort to find epigenetic biomarkers of transgenerational disease. These results will further our understanding of the mechanisms governing epigenetic transgenerational inheritance and may lead in the future to the use of epigenetic biomarkers that will help predict an individual’s susceptibility for specific diseases.

## Results

### Pathology analysis

To investigate the transgenerational actions of vinclozolin, pregnant female rats (F0) were exposed to vinclozolin or the vehicle control, dimethyl sulfoxide (DMSO), during days 8 to 14 of gestation. The resulting F1 generation offspring (direct fetal exposure) were then bred, avoiding inbreeding, to produce the F2 generation (direct germline exposure). The F2 generation offspring were bred in the same manner to produce the F3 generation offspring (transgenerational). Ten different F0 generation female lineages were generated so that no sibling or cousin breeding was used in later generations avoiding inbreeding artifacts. All rats were bred at 90 days of age when no disease was present, then aged to 1 year and sacrificed for tissue collection and pathology analysis. Testis, prostate, kidney, ovary and gonadal fat were collected and processed for histopathology analysis. The pathologies for each tissue were analyzed according to specific parameters and the rate of disease was determined for each tissue type and generation as described in the Methods [29]. In addition, the weaning weight, age at puberty and weight at the time of sacrifice were measured.

**Fig 1:**
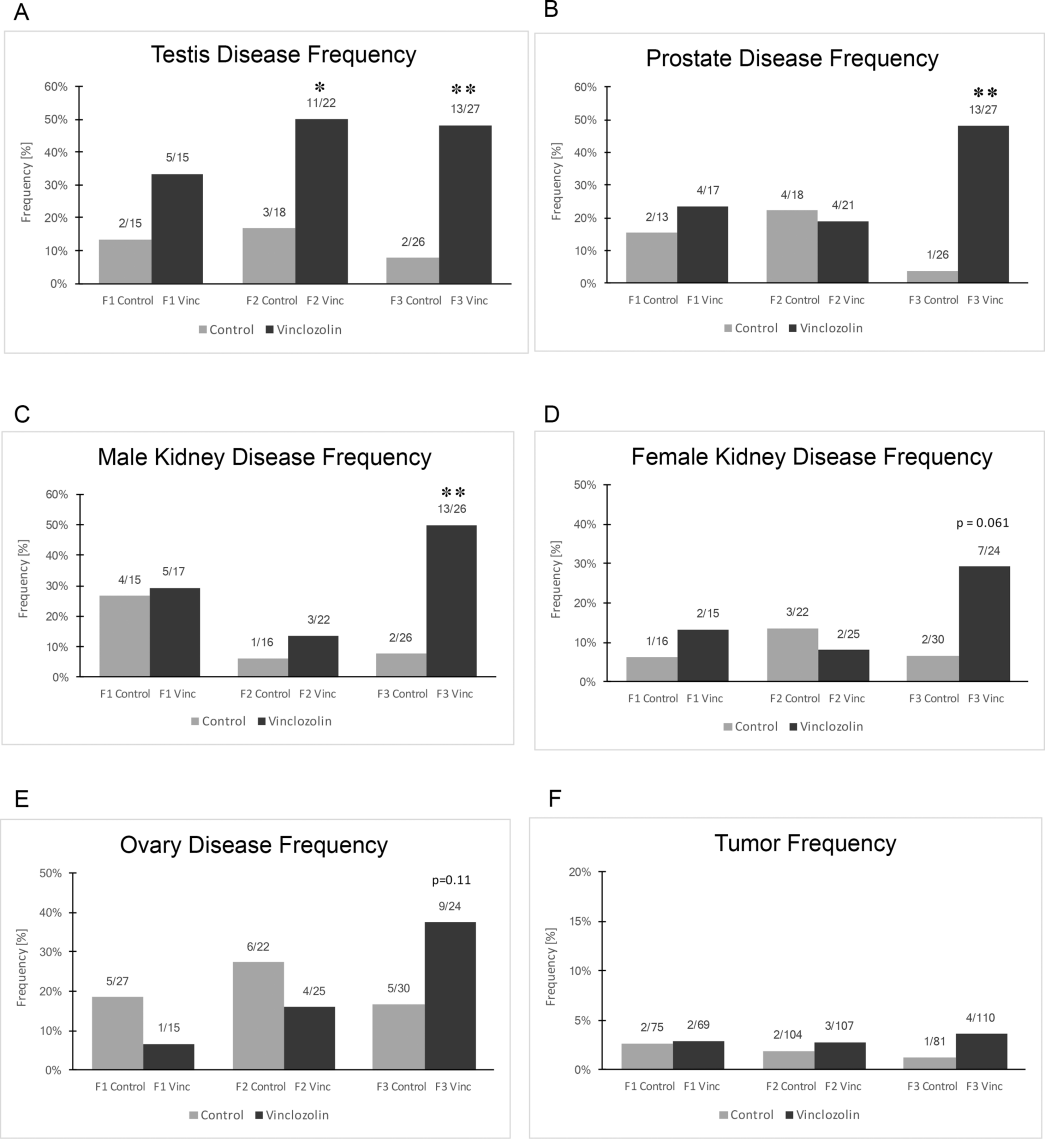
Pathology that shows the percentage of animals in each treatment group that were designated diseased for (A) Testis (B) Prostate (C) Male kidney (D) Female kidney, (E) Ovary, (F) Tumor. The proportion of affected animals over the evaluated population is shown over each bar. Asterisks indicate statistical significance by Fisher’s exact test (*) p < .05 and (**) p<0.01.

**Fig 2:**
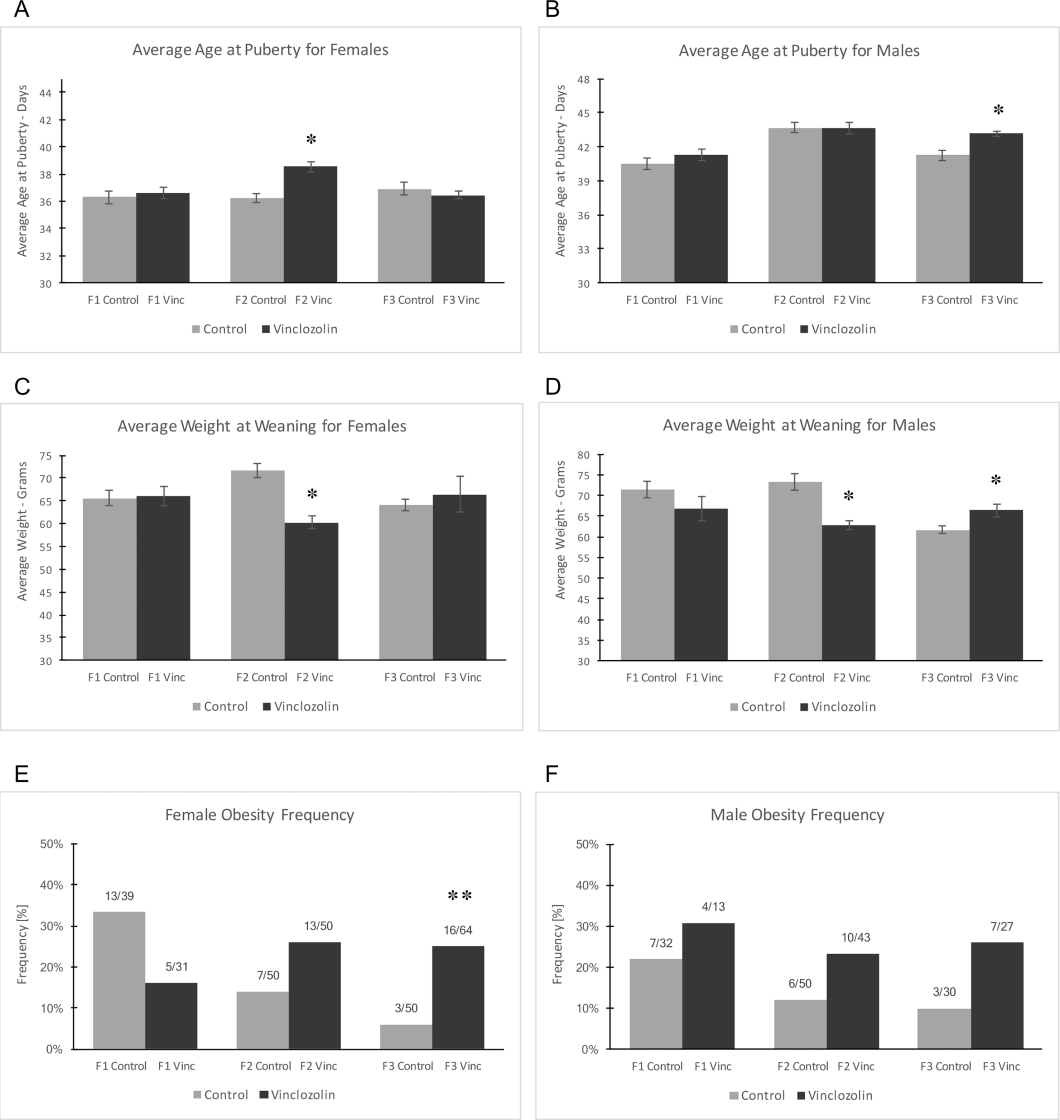
Pathology analysis indicates average age at puberty (A & B), weight at weaning for males and females (C & D), and obesity frequency (E & F). Error bars show the standard error of the mean (SEM) for A–D. The proportion of affected animals over the evaluated population and frequency of disease is shown for E & F. Asterisks indicate statistical significance by Student’s t-test (A–D) or Fisher’s exact test (E & F) *p<0.05 **p<0.01.

The F0 generation females were observed for 3–6 months after pregnancy, and no overt toxicity was noted. In the F1 generation, no significant abnormalities were observed in the assessed tissues (Figs 1 & 2, and S1–S3 Tables), nor were there significant effects on weaning weight, age at puberty, or weight at euthanization. In contrast to previous studies [29], these results indicate that there was little to no overt toxicity of the direct fetal exposure.

Testis disease was characterized by the presence of atrophied seminiferous tubules, vacuoles in the basal region of the tubule, and germ cells sloughed into the lumen of the seminiferous tubules. In addition, spermatogenic cell apoptosis was evaluated and is associated with reduced sperm count and motility [28]. In the F1 generation, the incidence of testis disease in the vinclozolin lineage males was not significantly higher than in controls, Fig 1A. In the F2 and F3 generations of vinclozolin lineage males there was a significantly higher rate of testis disease (p<0.01), Fig 1A. Therefore, there was transgenerational (F3 generation) testis disease, Table 1 and S3 Table.

**Table 1:** Transgenerational F3 vinclozolin males.

	Puberty		Testis	Prostate	Kidney	Tumor	Lean	Obese	Multiple Diseases	Total Disease
Rat ID	Early	Late								
14V1-3-1-3	**-**	**-**	**+**	**+**	**-**	**-**	**-**	**+**	**+**	**3**
14V1-3-1-4	**-**	**-**	**-**	**-**	**+**	**-**	**-**	**-**	**-**	**1**
14V1-3-4-5	**-**	**-**	**-**	**+**	**+**	**-**	**-**	**-**	**+**	**2**
14V1-3-4-6	**-**	**-**	**-**	**-**	**+**	**-**	**+**	**-**	**+**	**2**
14V1-3-4-7	**-**	**-**	**+**	**-**	**-**	**-**	**-**	**-**	**-**	**1**
14V1-3-4-8	**-**	**-**				**-**			-	
14V1-3-4-9	**-**	**-**				**-**			-	
14V1-3-4-10	**-**	**-**				**-**			-	
14V1-3-4-11	**-**	**-**				**-**			-	
14V1-3-4-12	**-**	**-**				**-**			-	
14V2-3-3-7	**-**	**-**	**-**	**-**	**+**	**-**	**-**	**+**	**+**	**2**
14V2-3-3-8	**-**	**-**	**+**	**+**	**-**	**-**	**-**	**-**	**+**	**2**
14V2-3-3-9	**-**	**-**				**-**			-	
14V2-3-3-10	**-**	**-**				**-**			-	
14V3-3-2-5	**-**	**-**	**-**	**+**	**-**	**-**	**-**	**+**	**+**	**2**
14V3-3-2-7	**-**	**-**				**-**			-	
14V3-3-2-6	**-**	**-**	**-**	**-**	**-**	**-**	**-**	**-**	-	
14V3-3-2-8	**-**	**-**				**-**			-	
14V3-3-8-6	**-**	**-**	**-**	**-**	**+**	**-**	**+**	**-**	**+**	**2**
14V3-3-8-7	**-**	**-**	**+**	**-**	**+**	**-**	**-**	**-**	**+**	**2**
14V3-3-8-8	**-**	**-**				**-**			-	
14V6-3-6-6	**-**	**-**	**-**	**-**	**+**	**-**	**-**	**-**	**-**	**1**
14V6-3-6-7	**-**	**-**	**+**	**-**	**+**	**-**	**-**	**-**	**+**	**2**
14V6-3-6-8	**-**	**-**				**-**			-	
14V6-3-7-2	**-**	**-**	**+**	**-**	**+**	**-**	**+**	**-**	**+**	**3**
14V6-3-7-3	**-**	**-**				**-**			-	
15V14-3-14-6	**-**	**-**	**-**	**+**	**-**	**-**	**-**	**-**	**-**	**1**
15V14-3-14-7	**-**	**-**	**+**	**-**	**+**	**-**	**-**	**-**	**+**	**2**
15V14-3-14-8	**-**	**-**	**+**	**-**	**-**	**-**	**-**	**+**	**+**	**2**
15V14-3-14-9	**-**	**-**	**-**	**+**	**-**	**-**	**-**	**+**	**+**	**2**
15V14-3-14-10	**-**	**-**				**-**			-	
15V14-3-14-11	**-**	**-**				**-**			-	
15V14-3-14-12	**-**	**-**				**-**			-	
15V14-3-14-13	**-**	**-**				**-**			-	
15V14-3-15-10	**-**	**-**	**+**	**-**	**-**	**-**	**-**	**-**	**-**	**1**
15V14-3-15-11	**-**	**-**	**+**	**+**	**+**	**-**	**-**	**-**	**+**	**3**
15V14-3-15-7	**-**	**-**	**+**	**+**	**-**	**-**	**-**	**+**	**+**	**3**
15V14-3-15-8			**+**	**+**	**-**	**-**	**-**	**-**	**+**	**2**
15V14-3-15-9	**-**	**-**	**-**	**+**	**-**	**-**	**-**	**-**	**-**	**1**
15V15-3-17-9			**+**	**+**		**-**	**-**	**-**	**+**	**2**
15V15-3-19-8	**-**	**-**				**-**			-	
15V15-3-19-7	**-**	**-**				**-**			-	
15V25-3-20-5	**-**	**-**	**-**	**-**	-	**-**	**-**	**-**	-	
15V25-3-20-6	**-**	**-**	**-**	**+**	**+**	**-**	**-**	**+**	**+**	**3**
15V25-3-20-7	**-**	**-**	**-**	**+**	**+**	**-**	**-**	**-**	**+**	**2**
15V25-3-20-9	**-**	**+**				**+**			**+**	**2**
**Affected**	**0**	**1**	**13**	**13**	**13**	**1**	**3**	**7**	**20**	
**Population**	**44**	**44**	**27**	**27**	**26**	**46**	**27**	**27**	**46**	

Vinclozolin F3 generation male pathology. The specific disease pathologies are indicated and a (+) indicates the presence of the disease and (-) the absence of the disease. Individual rat data are presented with the affected and total population indicated.

Prostate disease was characterized by the presence of vacuoles, atrophic epithelial cells, or hyperplastic epithelium in the prostate glandular epithelium [29]. In the F1 and F2 generation of vinclozolin lineage males the incidence of prostate disease was not different than that of controls, Fig 1B. In the F3 generation, vinclozolin lineage males had a significantly higher rate of prostate disease (p<0.01) compared to the controls, Fig 1B. Transgenerational prostate disease affected roughly 50% of the F3 vinclozolin lineage males, Table 1 and S3 Table.

Kidney disease was characterized by a reduction in the size of glomeruli, thickening of the Bowman’s capsule, and the presence of cysts [29]. In F1 and F2 generation males and females the incidence of kidney disease in vinclozolin lineage rats did not differ significantly from the controls, Fig 1C & 1D. However, in the F3 generation vinclozolin lineage males there was a significantly higher rate of kidney disease (p<0.01). In the F3 vinclozolin lineage females there was a trend toward increased kidney disease (P = 0.06), Fig 1D. Thus, kidney disease was a transgenerational disease in vinclozolin lineage males affecting approximately 50% of the population, Table 1 and S3 Table.

Ovarian disease was characterized by the development of polycystic ovaries with an increase in the number of small and large cysts, or by a decrease in the size of the primordial follicle pool [32]. There were no significant (p<0.05) differences in the incidence of ovarian disease between vinclozolin and control lineages in the F1, F2 or F3 generations, Fig 1E. However, in the F3 generation there was a trend toward an increased incidence of ovarian disease at a p value of 0.11, Fig 1E.

During the ageing of the control and vinclozolin lineage colonies all animals were monitored for the development of tumors. The incidence of tumor development was analyzed across the generations for females and males combined. In all three generations, compared to the controls, the rate of tumor development was not significantly different, and for all populations the overall rate was below 5%, Fig 1F. The most predominant tumor types that developed in the male or female were mammary tumors and tumor histopathology analysis generally identified adenomas or sarcomas of the tissues. Females had a higher rate of mammary tumor development than males, S3 Table.

The age of puberty was measured for every animal, and the average age at puberty was analyzed for males and females. In the F1 generation, there was no significant difference between the control and vinclozolin lineage males or females, Fig 2A & 2B. In the F2 generation, the average age at puberty was significantly later (p<0.05) for vinclozolin lineage females, but there was no effect on males, Fig 2A & 2B. For the F3 generation there was no significant difference in the age at puberty for females, but the vinclozolin lineage males had a significantly later average age at puberty, Fig 2A & 2B and S3 Table.

At the time of weaning the weight was measured for each pup, and the average weight at weaning was analyzed for all generations. There was no significant effect for males or females in the F1 generation, Fig 2C & 2D. However, in the F2 generation, the vinclozolin lineage females had a significantly lower average weight at weaning compared to the control females, and the same occurred in the F2 vinclozolin lineage males, Fig 2C & 2D. In the F3 generation, there was no significant effect on the females, but the vinclozolin lineage males had a significantly higher average weight at weaning, Fig 2C & 2D. Therefore, the vinclozolin lineage transgenerational males had an increased age at puberty and increased weight at weaning.

Rats were evaluated for obesity at one year of age. Criteria taken into consideration included the measured area of adipocytes in gonadal fat pads, the rat body length and body-mass index, and an evaluation of abdominal adiposity at the time of sacrifice (see Methods). Male rats showed no difference in obesity rates between control and vinclozolin lineages, Table 1. Female rats showed a transgenerational increase in the rate of obesity in the F3 generation, Fig 2E & 2F and S3 Table.

Behavioral tests were performed with F3 generation animals as described in Methods to assess basal levels of anxiety behaviors and activity. Time spent by a rat in the walled closed arm of an Elevated Plus Maze (EPM) as opposed to the exposed open arm can correlate with altered anxiety levels. The number of times a rat crosses lines in an EPM or an Open Field (OF) test measures innate activity levels. The number of crossings into the open or closed arms of an EPM are thought to also correlate with activity (locomotor) level as it affects exploratory behavior. F3 generation female rats showed no difference from controls in behavioral tests, S1 Fig. However, F3 generation vinclozolin lineage males spent significantly less time in the closed arm of the EPM, and there was a trend (p = 0.1) toward them spending more time in the open arm, S1A & S1B Fig. Vinclozolin lineage males also made more attempts (i.e. line crossings) into both the open and closed arms of the EPM, S1C & S1D Fig, which was reflected in an increased number of EPM total attempts, S1E Fig. This indicator of increased activity level was not seen in the open field test where line crossings were not statistically different between vinclozolin and control lineages, S1F Fig. Therefore, no overt behavior effects were observed for the F3 generation females and the males had a trend for increased locomotor activity.

**Fig 3:**
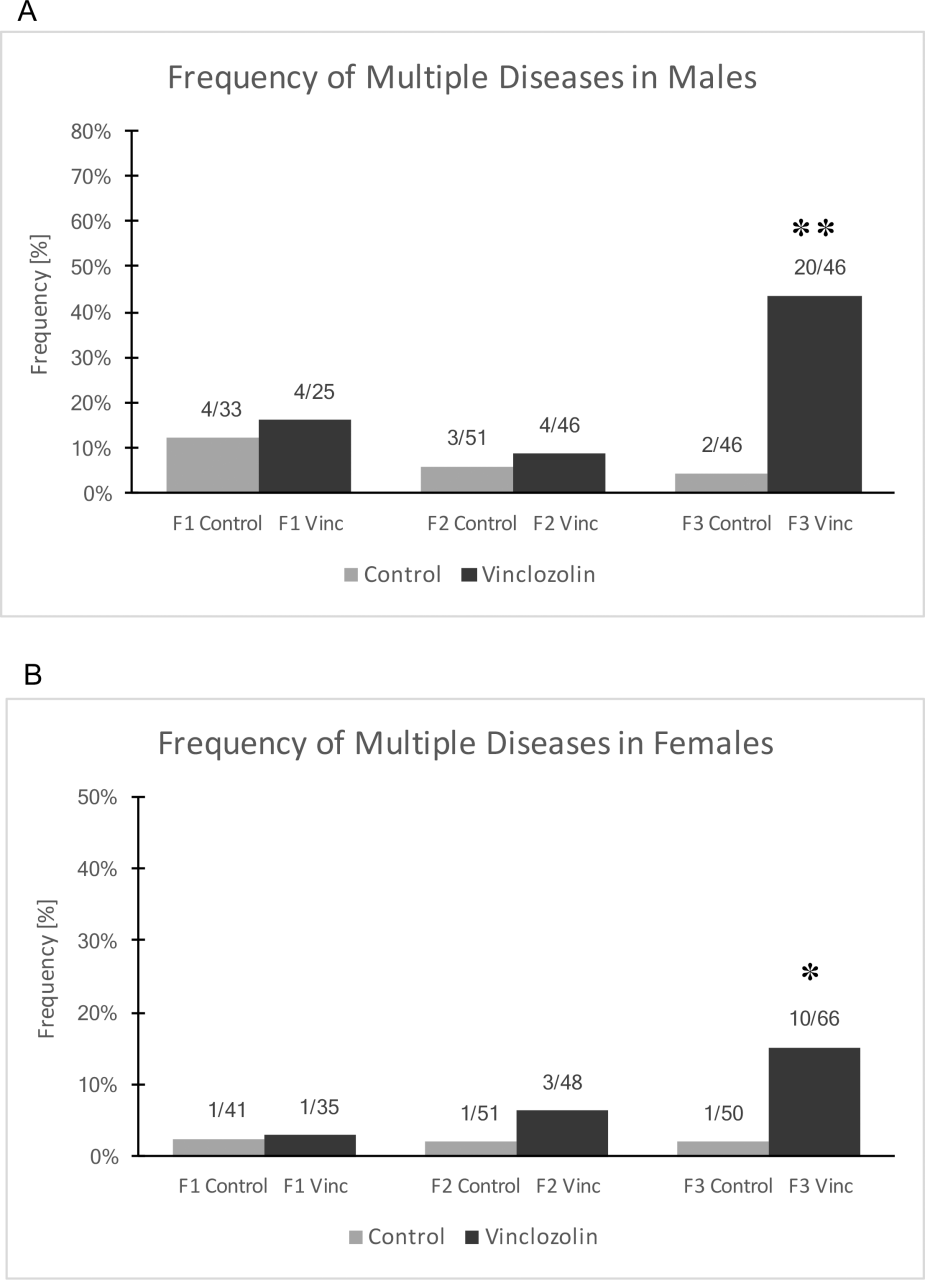
Frequency of multiple diseases is the proportion of animals having two or more diseases (A & B). The proportion of affected animals over the evaluated population is shown over each bar. Asterisks indicate statistical significance by Fisher’s exact test (*) p < .05 and (**) p<0.01.

The disease or abnormalities described above that occurred for each rat are listed in S1, S2 and S3 Tables for the F1, F2 and F3 generations, respectively. Some rats were shown to have more than one disease or abnormality, termed multiple diseases, Fig 3A & 3B. There was a transgenerational increase in the proportion of rats that had multiple diseases or abnormalities in both F3 generation males, Table 1, and F3 generation females, S3 Table.

### Disease associated sperm epimutations -

**Fig 4:**
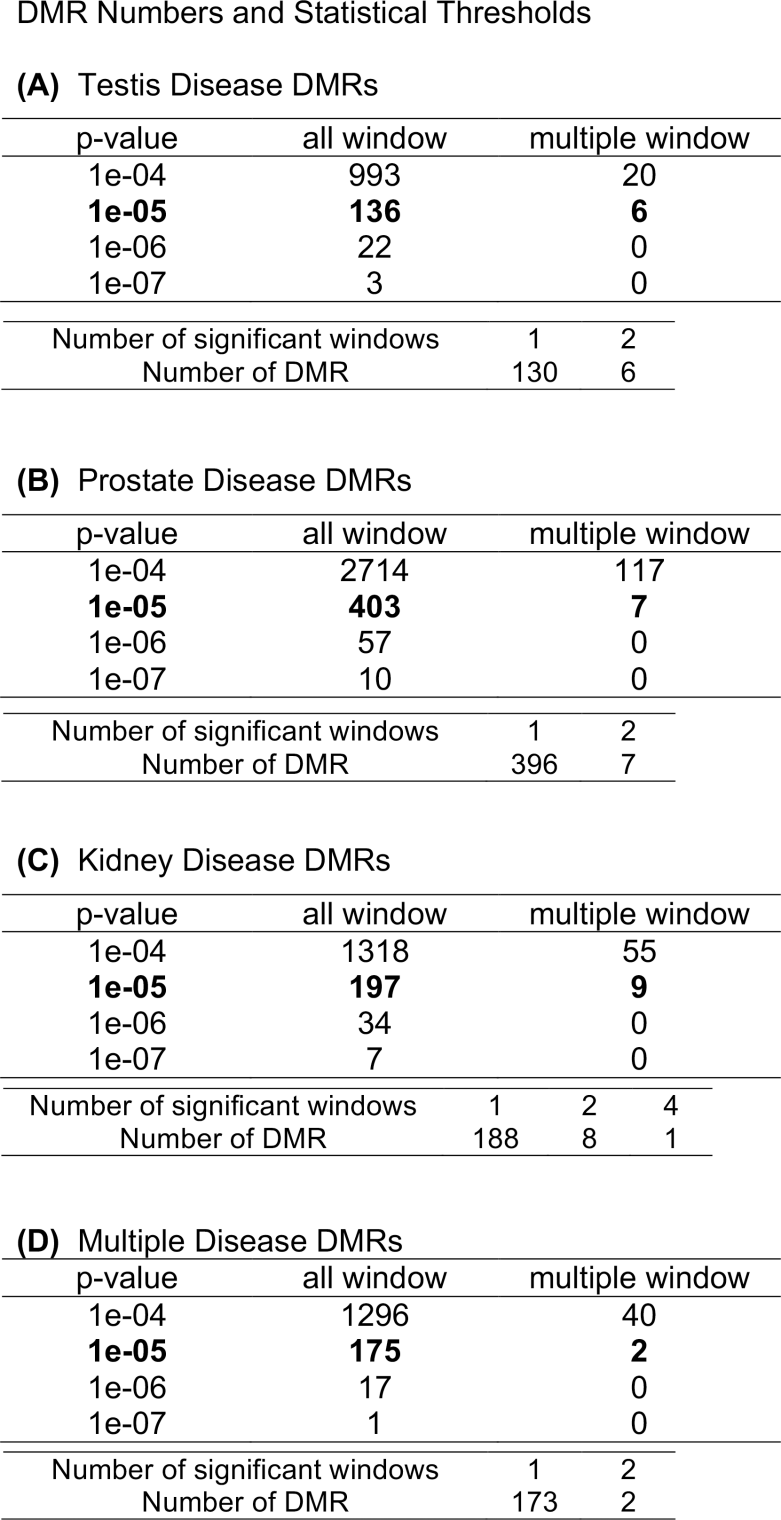
The number of F3 generation DMRs found using different p-value cutoff thresholds. The all window column shows all DMRs. The multiple window column shows the number of DMRs containing at least two significant windows. The bold column shows the number of DMR with each specific number of significant windows at a p-value threshold of 1e-05. (A) Testis disease DMRs (B) Prostate disease DMRs (C) Kidney disease DMRs (D) Multiple diseases DMRs.

The epigenetic transgenerational inheritance of disease and abnormalities requires the germline transmission of epimutations [1, 44, 45]. Previously F3 generation sperm have been shown to have differential DNA methylation regions (DMRs) induced by a variety of environmental toxicants [4, 7, 33]. Interestingly, the sperm DMRs appear to be largely unique to the specific environmental exposure [7]. The current study investigated the sperm epimutations in the F3 generation vinclozolin lineage males for different disease conditions, Table 1. Since the F1 and F2 generation sperm epigenetic alterations are due to direct exposure, the focus was on the transgenerational F3 generation. Sperm samples from individual F3 generation vinclozolin lineage males were processed and analyzed to characterize the sperm epigenome of each rat. This was required to identify specific disease-associated biomarkers as discussed below. The epigenetic analysis procedure involved a methylated DNA immunoprecipitation (MeDIP) followed by next generation sequencing (MeDIP-Seq) as described in the Methods [41]. Those DMRs between the vinclozolin lineage individuals with and without a specific pathology were identified. The genome was divided into 100bp windows for DMR identification. Vinclozolin lineage testis disease, prostate disease, kidney disease and multiple diseases occurrence provided sufficient numbers of diseased individuals for investigation, Table 1 and S3 Table. The majority of DMRs were single window DMRs, but some had multiple adjacent windows, and both data sets are shown in Fig 4 with different p-value thresholds. A p-value of p<10^−5^ was used to explore each disease biomarker dataset. Additionally, FDR adjusted p-values were also calculated to incorporate a multiple-testing correction into the analysis. An FDR p-value threshold of 0.3 generally identified a similar number of DMRs as an edgeR p-value at p<10^−6^, but this is less stringent than the standard FDR of 0.1. Therefore, the data has variability which appears to be due in part due to the low number of animals without disease and to the presence of multiple diseases in most animals, Table 1 and S3 Table. Although DMRs at all p-values for the different disease states are potentially important, the selected DMR data sets at p<10^−5^ were further investigated and are presented to demonstrate the phenomenon discussed. The DMR lists for each of the disease signatures are presented in S4–S7 Tables.

**Fig 5:**
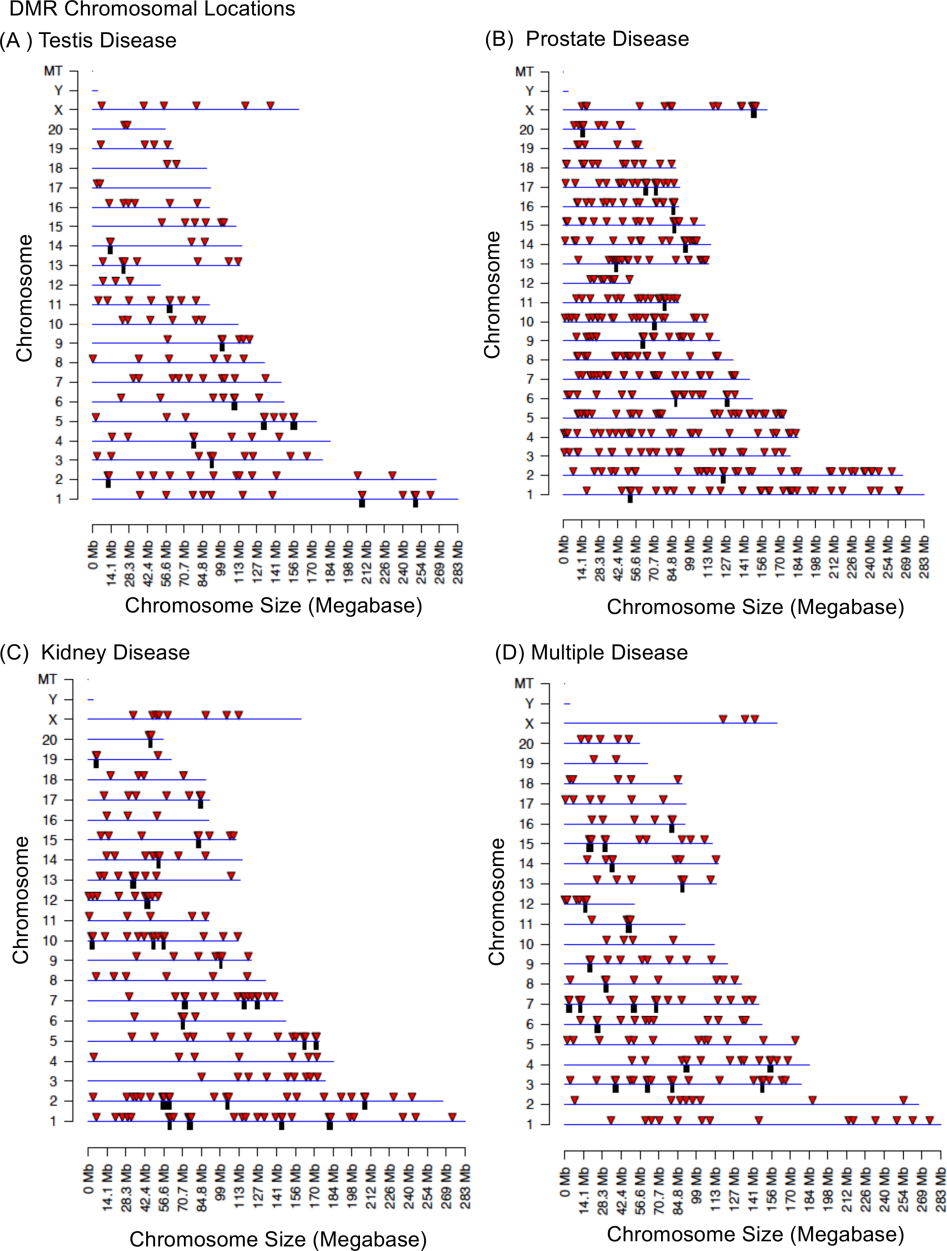
Chromosomal locations of DMR on the individual chromosomes. All DMRs at a p-value threshold of p<1e-05 are shown for (A) Testis disease (B) Prostate disease (C) Kidney disease (D) Multiple diseases. The red arrowheads indicate the location of a DMR and black box the location of a cluster of DMR.

**Fig 6:**
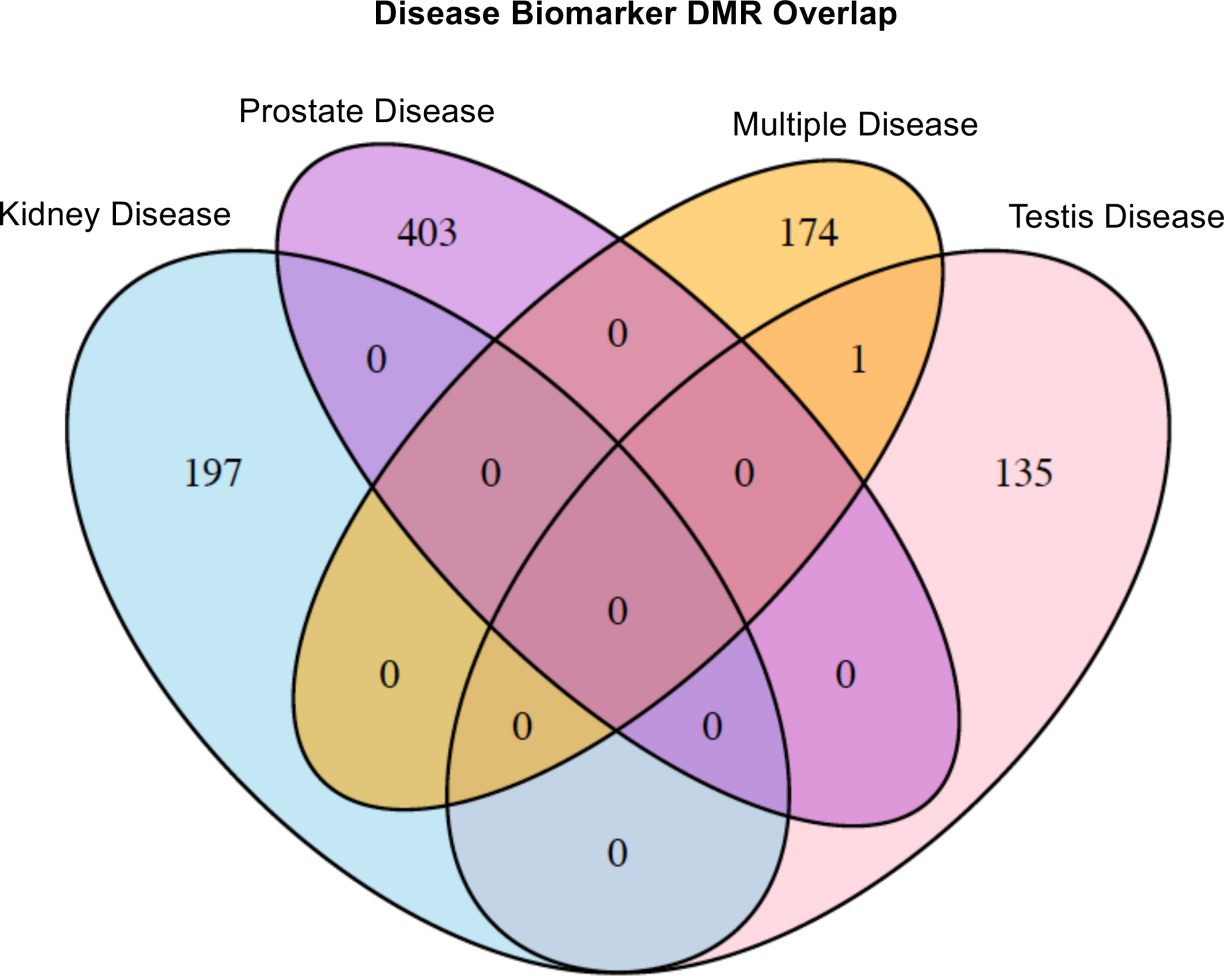
Overlap of different disease group DMRs.

The chromosomal locations of the vinclozolin testis disease DMRs or epimutation signature are presented in Fig 5A showing all chromosomes except Y are involved. This testis epimutation signature provides a potential biomarker for the transgenerational testis disease phenotype. A similar analysis for prostate disease (Fig 5B), kidney disease (Fig 5C) and multiple diseases (Fig 5D) in the F3 generation vinclozolin lineage males was performed. For each of the different disease epimutation biomarkers (i.e. sets of DMRs) the epimutations were present on all chromosomes except the Y chromosome. The epimutation disease signatures all contained clusters of DMRs (black boxes). The lists of each disease DMRs are presented in S4–S7 Tables. Interestingly, each disease had a unique set of epimutations with negligible overlap, Fig 6. Therefore, the disease biomarkers appear to have disease specificity.

**Fig 7:**
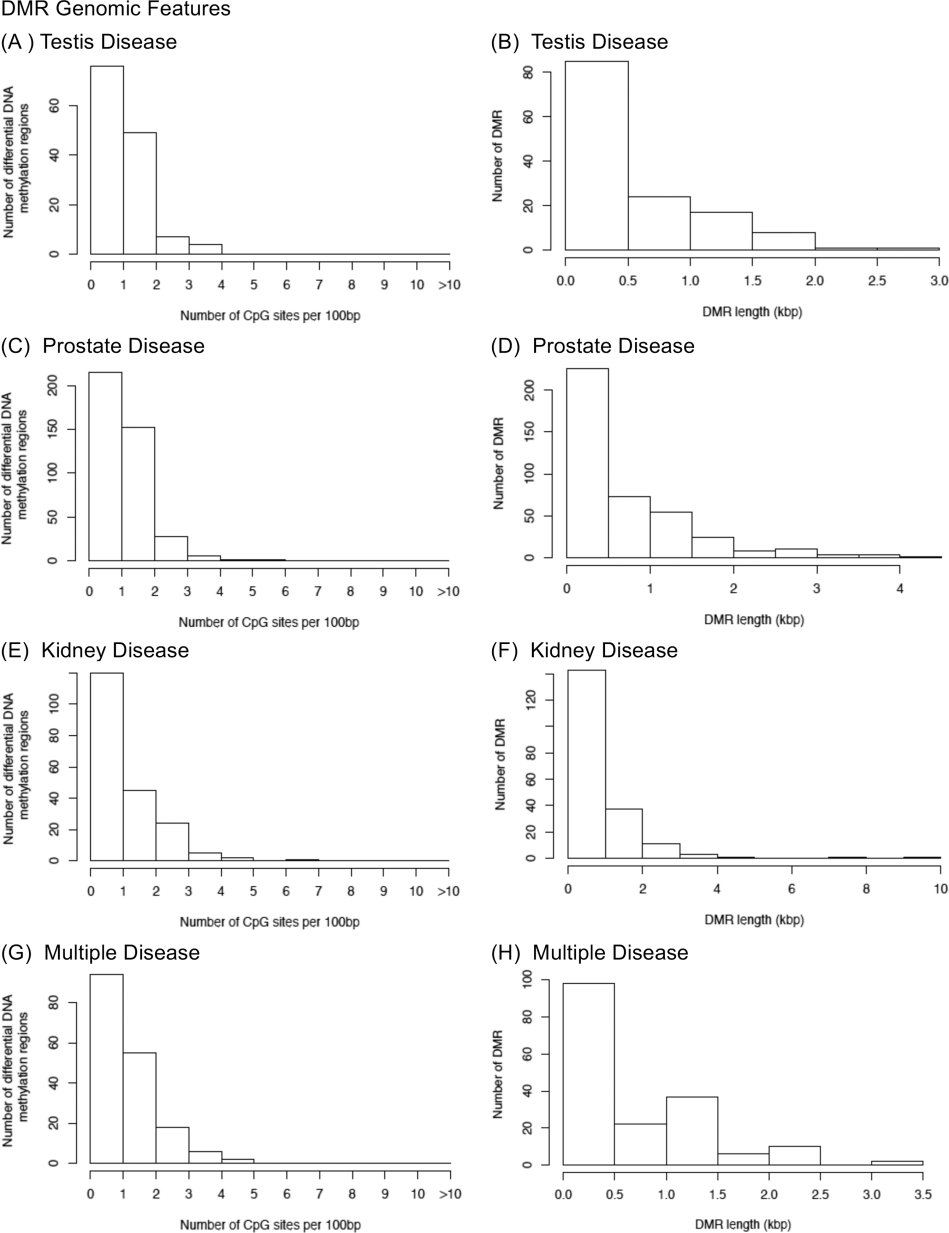
Genomic features of F3 generation DMRs. The number of DMRs at different CpG densities at a p-value threshold of 1e-05 are shown; (A) Testis disease (C) Prostate disease (E) Kidney disease (G) Multiple diseases. The DMR lengths at a p-value threshold of 1e-05 are shown; (B) Testis disease (D) Prostate disease (F) Kidney disease (H) Multiple diseases.

The genomic features of the DMRs were examined for each disease epimutation signature. The CpG density was found for all disease biomarkers to be predominantly 1–2 CpG/100bp, Fig 7. Those low density CpG regions associated with the DMRs are called CpG deserts [46]. The length of the DMRs for all the disease epimutation signatures was predominantly 0.5 to 1 kb in size, Fig 7. Therefore, 5–10 CpGs are present in the DMRs associated with the disease epimutation signatures, S4–S7 Tables. The increase or decrease in DNA methylation is presented as the maximum fold (i.e. ratio) change (disease/non-disease) column in the DMR lists for each disease, S4–S7 Tables. Increases and decreases in DNA methylation are observed with most involving over four-fold change.

**Fig 8:**
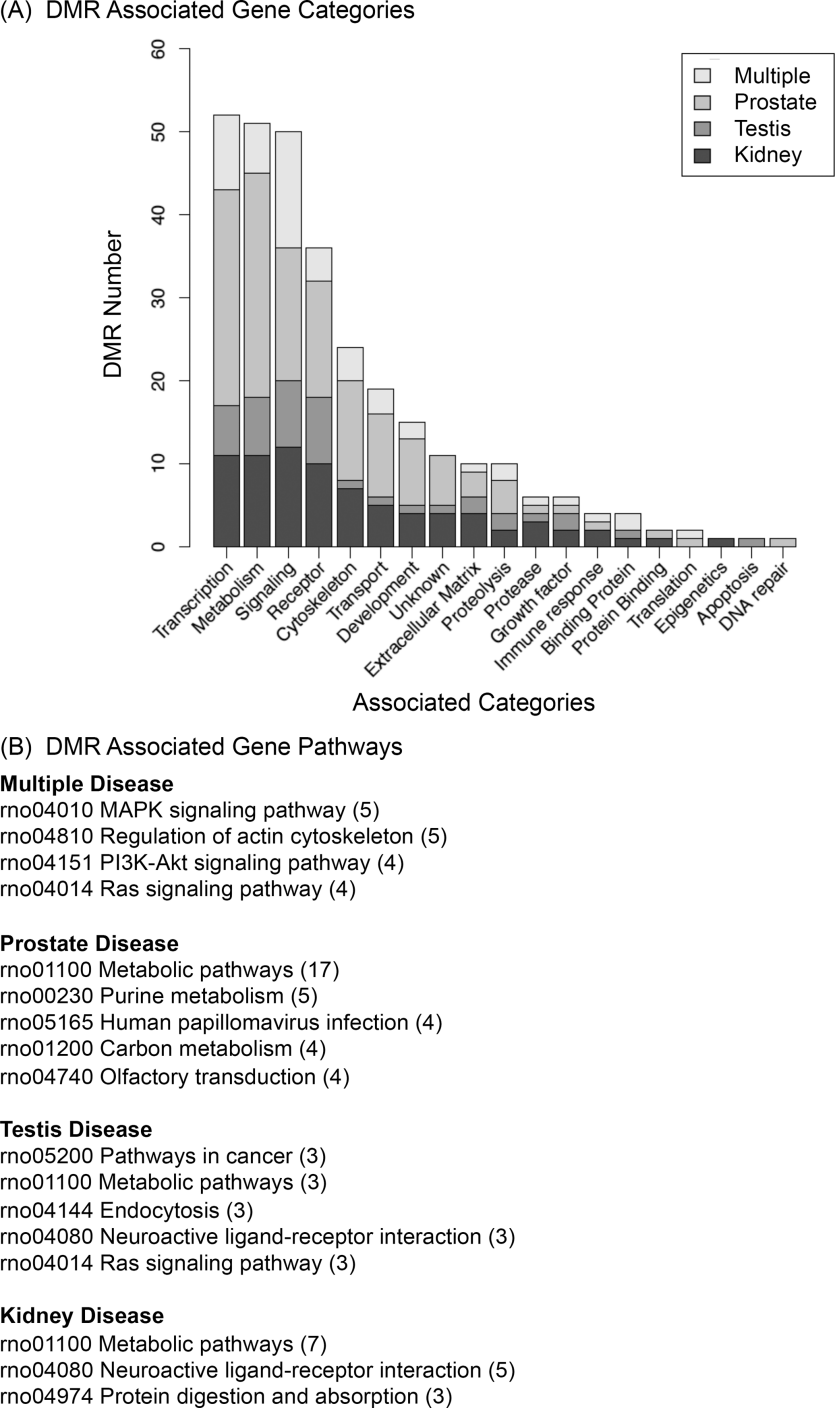
DMR associated gene function categories. (A) DMR associated gene categories for each disease DMR data set. Color code inset for disease type. (B) DMR associated gene pathways (KEGG) with top five pathways with the most DMR genes (bracket) involved.

The DMR associated genes are presented in S4–S7 Tables for each disease. The majority of DMRs do not have associated genes within 10 kb, but many DMRs do and the gene symbol and functional gene categories are listed. The gene functional categories are shown in Fig 8A for each set of disease DMR biomarker associated genes, S4–S7 Tables. Similar major functional categories include transcription, metabolism, signaling and cytoskeleton for each disease, Fig 8. A pathway analysis was performed and pathways with a minimum of three genes involved are presented for each disease DMR data set, Fig 8B. The top five pathways with the highest number of DMR associated genes are listed, with the number of genes given in the brackets. Minimal overlap in the specific pathways are observed.

**Fig 9:**
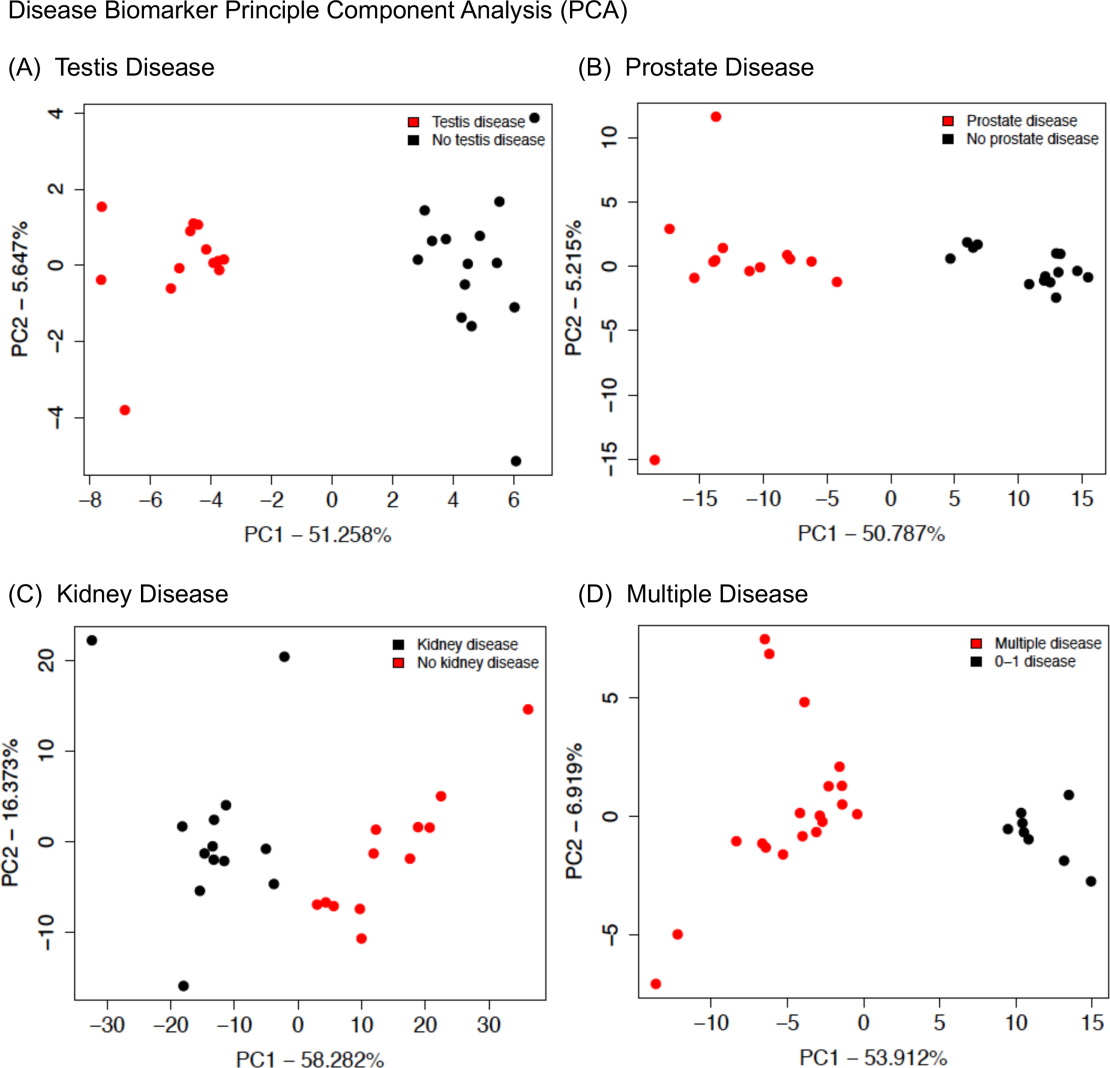
A principle component analysis with samples plotted by the first two principal components. The underlying data is the RPKM read depth for all DMR genomic windows with an edgeR p-value < 1e-5. (A) Testis disease (B) Prostate disease (C) Kidney disease (D) Multiple diseases.

**Fig 10:**
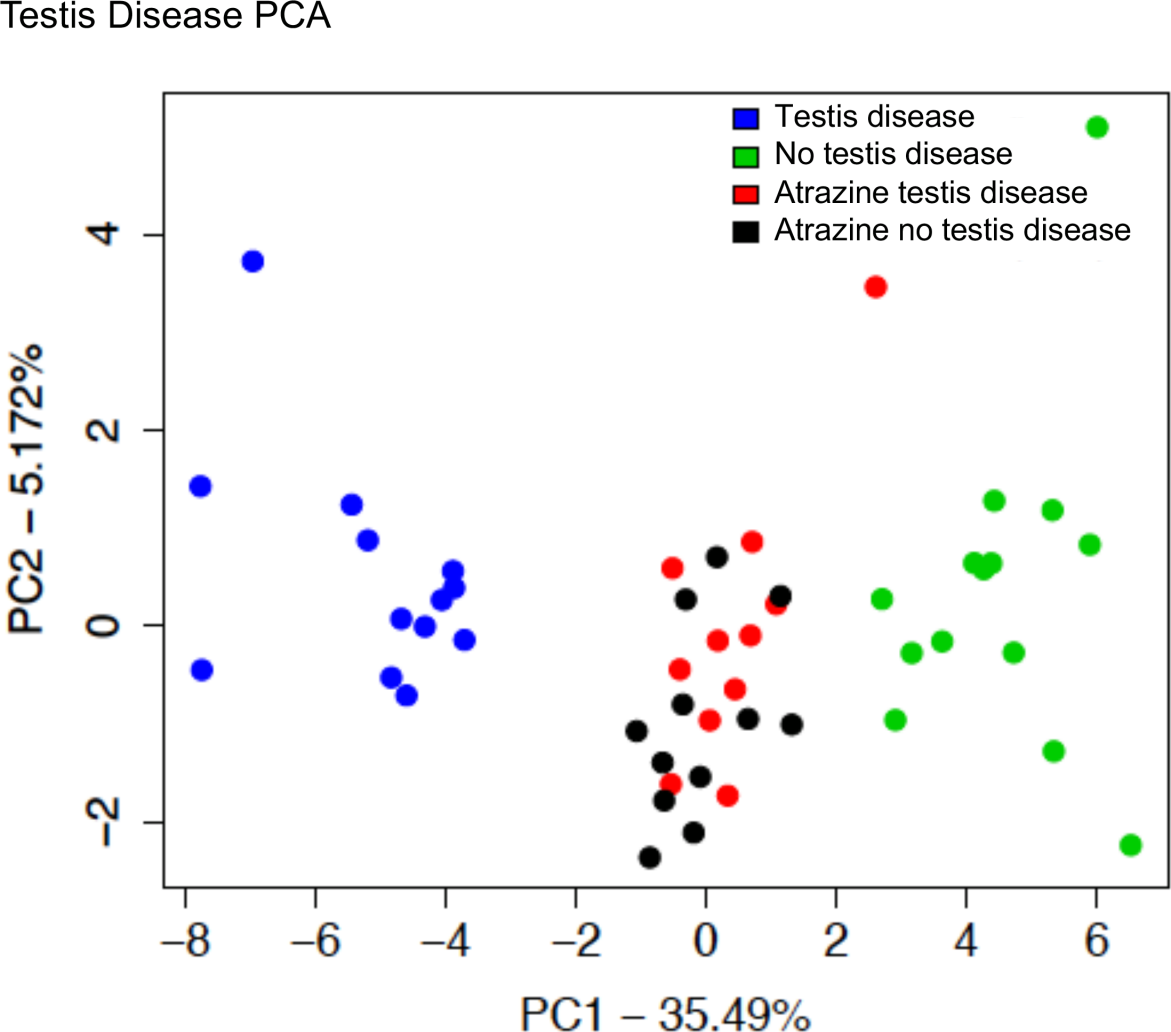
A principle component analysis with samples plotted by the first two principal components. The underlying data is the RPKM read depth for all genomic windows with an edgeR p-value < 1e-5. Samples from an independent atrazine 1yr project have been included as testis disease or no testis disease for comparison.

A principle component analysis (PCA) of the DMRs in each of the specific disease epimutation signatures was performed, Fig 9. Generally, the DMRs associated with the individuals with disease clustered separately from those associated with individuals without disease. As expected, distinct clustering for testis disease (Fig 9A), prostate disease (Fig 9B), kidney disease (Fig 9C) and multiple diseases (Fig 9D) was observed. A test set of disease samples not involved in the DMR analysis was not available for the prostate, kidney or multiple biomarker validation, but a previous testis disease set of analyzed samples was available for testis disease in an atrazine induced epigenetic transgenerational inheritance study [20]. The specific testis disease sequencing data from this atrazine lineage F3 generation sperm study was used in the same PCA analysis with the vinclozolin F3 generation sperm analysis, Fig 10. Although the vinclozolin F3 generation testis disease epimutation DMRs clustered separately from the non-disease individuals, the atrazine DMRs did not associate with either the testis disease or non-disease clusters. Therefore, the specific exposure lineage may impact the epimutation signature found for the specific disease epimutation biomarkers.

## Discussion

The objectives of the current study were to investigate the epigenetic transgenerational inheritance of disease and abnormalities after ancestral exposure of gestating rats to vinclozolin, and to characterize the associated molecular epigenetic changes that were found in F3 generation sperm associated with specific diseases. It is only through germ cells such as sperm that information is transmitted from one generation to the next. Results from the current study help describe the epigenetic mechanisms that are a part of that disease inheritance process. Gestating female rats were exposed to vinclozolin during days 8–14 of fetal development. Since this is the time of fetal gonadal sex determination a direct exposure of the developing F1 generation fetus occurs, as does exposure of the developing germ cells within the fetus that will produce the F2 generation [1]. Therefore, the F1 generation had direct toxicant exposure to both their somatic cells and germ cells. Pathologies seen in F1 generation adult rats and epigenetic changes seen in F1 generation sperm are the result of direct vinclozolin exposure. The F2 generation rats were derived from directly exposed sperm and eggs, such that abnormalities are the result of a combination of direct exposure and generational transmission. The transgenerational F3 generation rats had no direct toxicant exposure and any abnormalities present in vinclozolin lineage animals are the result of germline transmission of epigenetic alterations. The altered germline epigenome will change the embryonic stem cell epigenome and transcriptome which impacts all somatic cell epigenomes and transcriptomes in the subsequent generation [2]. Given these different modes of exposure and epigenetic inheritance in each generation it is expected that the epigenetic changes present in the F1, F2 and F3 generations will differ [47, 48]. To assess the potential for transgenerational epigenetic biomarkers for disease the focus of the current study was on the F3 generation sperm epigenetics.

Certain pathologies and disease states were identified by histological examination of organs. It was found that F3 generation vinclozolin lineage rats had increased disease rates in the testis, prostate, kidney and ovary, demonstrating transgenerational inheritance of increased disease susceptibility, Figs 1 & 2 and Table 1. This epigenetic transgenerational inheritance of disease after exposure to vinclozolin is similar to what has been observed in previous research [4, 29–32]. However, in some of these studies increased rates of disease were also seen in F1 generation vinclozolin lineage testis, prostate, kidney and ovary [29, 30, 32]. Determining why previous [4, 29–32] F1 generation disease findings were different than those in the current study will require further investigation, but the transgenerational findings are confirmatory. In the current study, there was a significant transgenerational increase in the proportion of vinclozolin lineage animals that had a disease and an increase in the proportion of animals that have more than one disease or abnormality, Fig 3.

Female F3 generation vinclozolin lineage rats were found to have a significant transgenerational increase in obesity rates. Previous studies with vinclozolin in rats have not noted this phenotype. However, it has been reported to occur transgenerationally after ancestral DDT exposure [5]. In the current study obesity was more rigorously evaluated, making use of adipocyte size in the gonadal fat pad, as well as of body-mass index. Overall, observations confirm that the epigenetic transgenerational inheritance of disease susceptibility occurs with ancestral vinclozolin exposure.

The individual male sperm epigenomes were analyzed in the F3 generation vinclozolin lineage to potentially identify epimutations that may correlate with specific pathologies. The pathologies in the F3 generation vinclozolin lineage males that were the most predominant were testis, prostate and kidney disease, as well as multiple diseases individuals. Epimutation signatures for each of these disease conditions were identified, Figs 4 and 5. The testis disease, prostate disease, kidney disease and multiple diseases epimutation signatures were all distinct, Fig 6. Although an epimutation signature was found for each, there was greater variation than expected as seen with the higher FDR values. However, the pathology observed in Figs 1–3, Table 1 and S3 Table for the vinclozolin lineage individuals demonstrate the vast majority of animals had multiple diseases. Many of the males concurrently had testis, prostate and kidney disease. Therefore, the magnitude of the multiple diseases was high and a confounder to consider in the analysis. The higher variation was suggested by the FDR analysis. Further studies for epigenetic biomarkers for disease should consider larger populations of animals without disease. It is anticipated from the data presented that disease specific epigenetic biomarkers for disease will be feasible to identify, but the experimental design will need to reduce the multiple diseases confounder.

Interestingly, when an atrazine lineage F3 generation testis disease DMR data set [20] was compared to the vinclozolin lineage F3 generation testis disease DMR data set of the current study, negligible clustering was observed in the PCA analysis. In addition, the testis epimutation biomarker signatures for each were distinct. Although the variation in the current disease data sets need to be considered, the exposure lineage and impacts of the different toxicants appear to be a predominant aspect of the epimutation biomarker for disease observed. Those epimutations associated with the specific disease appear to be unique to the exposure. This needs to be considered in future studies of epimutations associated with specific disease. Perhaps a control population will be required to then compare to the toxicant induced individual transgenerational disease studied.

These data suggest a transgenerational disease or pathology specific epigenetic biomarker or diagnostic potentially could be developed and associated with the majority of the animals having that pathology. Future studies will need to consider the impacts of co-morbidities and multiple diseases in the identification and use of disease specific biomarkers. The observation that the epimutation signatures were distinct between all the disease conditions suggests epigenetic diagnostics or biomarkers may be disease specific and unique. Therefore, epigenetic biomarkers may provide a useful molecular diagnostic for disease and could have a significant impact on disease diagnosis and management. In addition, the current identification of the epimutation signature in sperm suggests the possibility of a preconception diagnostic that might predict future disease susceptibility in offspring.

## Methods

### Animal studies and breeding

Female and male rats of an outbred strain Hsd:Sprague Dawley®™SD®™ obtained from Harlan/Envigo (Indianapolis, IN) at about 70 to 100 days of age were maintained in ventilated (up to 50 air exchanges/hour) isolator cages (cages with dimensions of 10 ¾″ W×19 ¼″ D×10 ¾″ H, 143 square inch floor space, fitted in Micro-vent 36-cage rat racks; Allentown Inc., Allentown, NJ) containing Aspen Sani chips (pinewood shavings from Harlan) as bedding, and a 14 h light: 10 h dark regimen, at a temperature of 70 F and humidity of 25% to 35%. The mean light intensity in the animal rooms ranged from 22 to 26 ft-candles. Rats were fed ad lib with standard rat diet (8640 Teklad 22/5 Rodent Diet; Harlan) and ad lib tap water for drinking. During the procedures, rats were held in an animal transfer station (AniGard 6VF, The Baker Company, Sanford, ME) that provided an air velocity of about 0.5 inch.

To obtain time-pregnant females, the female rats in proestrus were pair-mated with male rats. The sperm-positive (day 0) rats were monitored for diestrus and changes in body weight. When pregnancy was confirmed, on days 8 through 14 of gestation [31] the females were administered daily intraperitoneal injections of vinclozolin (100 mg/kg BW/day, Chem Services, Westchester PA, USA) or dimethyl sulfoxide (vehicle) as previously described [7]. Treatment groups were designated ‘vinclozolin’ and ‘control’ lineages. The gestating female rats (n = 12 control, n = 10 vinclozolin) treated were considered to be the F0 generation. The offspring of the F0 generation rats were the F1 generation. Non-littermate females and males aged 70–90 days from F1 generation control or vinclozolin lineages were bred (n = 14 control, n = 13 vinclozolin) to obtain F2 generation offspring. The F2 generation rats were bred (n = 10 control, n = 12 vinclozolin) to obtain F3 generation offspring. No sibling or cousin breeding was used in later generations to avoid inbreeding artifacts. Only the pregnant F0 generation rats were treated directly with vinclozolin. The control and vinclozolin lineages were housed in the same rooms as previously described [1, 7, 29]. All experimental protocols for the procedures with rats were pre-approved by the Washington State University Animal Care and Use Committee (IACUC approval # 6252).

### Tissue harvest and histology processing

Rats at 12 months of age were euthanized by CO_2_ inhalation and cervical dislocation for tissue harvest. Body weight and length were measured at dissection. Testis, prostate, ovary, kidney, and gonadal fat pad were fixed in Bouin’s solution for 24 hours (Sigma) followed by 70% ethanol, then processed for paraffin embedding by standard procedures for histopathological examination. Tissue sections (5 μm) were made and were left either unstained and used for TUNEL analysis or stained with H & E stain as previously described [29] and examined for histopathologies.

### Histopathology examination and disease classification

Histology for each slide was examined by three independent observers blinded to the treatment groups. The compiled results were used to establish a cut-off to declare a tissue ‘diseased’ based on the mean number of histopathological abnormalities plus two standard deviations from the mean of control tissues by each of the three individual observers. This number was used to classify rats into those with and without testis, ovary, prostate or kidney disease in each lineage as previously described [5]. A rat tissue section was finally declared ‘diseased’ only when at least two of the three observers marked the same tissue section ‘diseased’.

Testis histopathology criteria included the presence of vacuoles in the seminiferous tubules, azoospermic atretic seminiferous tubules and ‘other’ abnormalities including sloughed spermatogenic cells in center of the tubule and a lack of a tubule lumen, as previously described [4, 5, 29]. Testis sections were also examined by Terminal deoxynucleotidyl transferase-mediated dUTP nick end labeling (TUNEL) assay (In situ cell death detection kit, Fluorescein, Sigma, St. Louis, MO) as per manufacturer’s instructions. An increase in TUNEL-positive germ cells was considered an abnormality. Prostate histopathology criteria included the presence of missing cells that create vacuoles in the glandular epithelium, atrophic epithelial layer of ducts, and hyperplasia of prostatic duct epithelium as previously described [30, 49]. Kidney histopathology criteria included reduced size of glomerulus, thickened Bowman’s capsule and the presence of proteinaceous fluid-filled cysts, as previously described [9, 29].

Ovary sections were stained with hematoxylin and eosin and three stained sections (150 μm apart) through the central portion of the ovary with the largest cross section were evaluated. Ovary sections were assessed for primordial follicle loss and polycystic ovary disease, as previously described [32]. Primordial follicle loss was determined by counting the number of primordial follicles per ovary section and averaging across three sections. An animal was scored as having primordial follicle loss if the primordial follicle number in a section was less than that of the control mean minus two standard deviations. Primordial follicles have an oocyte surrounded by a single layer of either squamous or both squamous and fewer than three cuboidal granulosa cells [50, 51]. Follicles had to be non-atretic and showing an oocyte nucleus in order to be counted. Polycystic ovaries were determined by microscopically counting the number of small and large cystic structures per section averaged across three sections. A polycystic ovary was defined as having a number of small and / or large cysts that was more than the control mean plus two standard deviations. Cysts were defined as fluid-filled structures of a specified size that were not filled with red blood cells and which were not follicular antra. Small cysts were 50 to 250 μm in diameter measured from the inner cellular boundary across the longest axis, while large cysts were >250 μm in diameter. Percentages of females with primordial follicle loss or polycystic ovarian disease were computed.

Obesity and the lean phenotype were assessed with an increase in adipocyte size (area), increased body weight and abdominal adiposity, as previously described [20]. The obesity classification has been previously defined as these abnormalities and the presence of associated pathologies [52–56]. Body mass index (BMI) was calculated at one year of age with weight (g) / length (cm)^2^. Length was measured from the animals nose to the base of tail. Gonadal fat pad slides were imaged using a Nikon Eclipse E800 microscope (10x) with an AVT Prosilica GE1050C Color GigE camera. Five field of view images were captured per slide in varying parts of the fat pad. Adipocyte size was measured converting pixels into microns using Adiposoft [57]. Measurements of the 20 largest cells from each image for a total of 100 were averaged as hypertrophic cells are the most metabolically relevant and susceptible to cell death [58]. Obesity and lean phenotypes were determined utilizing the mean of the control population males and females and a cut off of 1.5 standard deviations above and below the mean.

### Behavior analysis

Behavior analysis was performed with both elevated plus maze and open field tests as previously described [59, 60]. F3 generation male and female Sprague-Dawley rats from control and vinclozolin lineages were used for the behavioral studies at 11 months of age. Elevated Plus-Maze tests were carried out between 9–10 am, and the same rats were always tested the following day at the same time for the Open Field test. Elevated plus maze data were obtained from 27 vinclozolin lineage males, 28 vinclozolin lineage females, 17 control males, and 27 control females. Open field data were obtained from 25 vinclozolin lineage males, 27 vinclozolin females, 18 control males, and 34 control females.

The elevated plus-maze consisted of a ‘‘plus”-shaped platform made of black opaque Plexiglas, with each platform 10 cm in width and 50 cm in length, creating a 10x10 cm neutral zone in the center. The plus-maze was elevated 50 cm from the floor. Two of the arms were enclosed with black Plexiglas walls 40 cm high, with no ceiling. The elevated plus-maze relies on the animal’s natural fear of open spaces, and the percent time spent on the open arms and percent of open arm entries comprises a general analysis of anxiety [61]. For this task, rats were placed individually into the center (neutral) zone of the maze, facing an open arm. Rats were allowed to explore for a 5 min period, and the number of open and closed arm entries and time spent on the open and closed arms were recorded. Entries were documented when a rat’s snout crossed into an open or closed arm. Animals were considered to be in the open or closed arms only when all four paws crossed out of the neutral zone.

The Open Field test consisted of a transparent Plexiglas 58x58 cm base and four 39.5 cm tall walls. The 58x58 cm base was divided up into a 4x4 grid with 14.7x14.7 cm sized squares made using red tape. The Open Field test has been validated for measuring motor behavior, ambulation and anxiety [62]. Rats were individually placed in the central 2x2 square and allowed to explore for a 5 min period. The duration spent in the central 2x2 area, duration spent in the surrounding (outside of the 2x2) area, and number of line crossings were recorded. Animals were considered to be in the central or surrounding area when all four paws were in those areas. A line crossing was counted when a rat’s snout crossed a tape line. Video recordings of the behavior were scored by two independent readers that were blinded to the animal identification.

### Statistical analyses for histopathological, obese/lean and behavioral data

For results that yielded continuous data (age at puberty, weight at euthanization, behavioral parameters) treatment groups were analyzed using a two-tailed Student’s t-test. Continuous data sets were confirmed to be normally distributed. For results expressed as the proportion of affected animals that exceeded a pre-determined threshold (testis, prostate, kidney or ovary disease frequency, tumor frequency, lean/obese frequency) groups were analyzed using Fisher’s exact test.

### Epididymal sperm collection and DNA isolation

The epididymis from each rat was dissected free of fat and connective tissue, a small cut made to the cauda and the tissue placed in 6 ml of phosphate buffered saline (PBS) for 20 minutes at room temperature and then kept at 4°C to immobilize the sperm. The epididymal tissue was coarsely minced and the released sperm centrifuged at 4,000 x *g* for 5 min and pellet resuspended in fresh NIM buffer and stored at -20°C until processed further. Fifty to 100 μl of rat sperm suspension were used for DNA extraction. The suspension was sonicated for five seconds (Fisher Sonic Dismembrator, model 300, power 25), then centrifuged 5 min at 6000 x g, supernatant discarded and sperm washed again to remove somatic cells and debris. Then the pellet was resuspended in 820 μL DNA extraction buffer and 80 μl 0.1M DTT added. The sample was incubated at 65°C for 15 minutes. Following this incubation 80 μl proteinase K (20 mg/ml) was added and the sample was incubated at 55°C for at least 2 hours under constant rotation. Then 300 μl of protein precipitation solution (Promega Genomic DNA Purification Kit, A795A) were added, the sample mixed thoroughly and incubated for 15 min on ice. The sample was centrifuged at 13,500 rpm for 30 minutes at 4°C. One ml of the supernatant was transferred to a 2 ml tube and 2 μl of glycoblue (Invitrogen, AM9516) and 1 ml of cold 100% isopropanol were added. The sample was mixed well by inverting the tube several times then left in -20°C freezer for at least one hour. After precipitation, the sample was centrifuged at 13,500 rpm for 20 min at 4°C. The supernatant was taken off and discarded without disturbing the (blue) pellet. The pellet was washed with 70% cold ethanol by adding 500μl of 70% ethanol, centrifuged for 10 min at 4°C at 20,000 xg and the supernatant discarded. The tube was spun again briefly to collect residual ethanol at bottom of tube and then as much liquid as possible was removed with gel loading tip. Pellet was air-dried at RT until it looked dry (about 5 minutes). Pellet was then resuspended in 100 μl of nuclease free water. For F3 generation each individual’s sperm DNA sample was analyzed separately for MeDIP-Seq.

### Methylated DNA immunoprecipitation (MeDIP)

Methylated DNA Immunoprecipitation (MeDIP) with genomic DNA was performed as follows. The F3 generation DNA from each rat was processed for MeDIP separately. The genomic DNA was sonicated using the Covaris M220, involving up to 6μg of individual animal sperm genomic DNA which was diluted to 130 μl with TE buffer (10mM Tris HCl, pH7.5; 1mM EDTA) in the appropriate Covaris tube. Covaris was set to 300 bp program and the program was run for each tube in the experiment. 10 μl of each sonicated DNA was run on 1.5% agarose gel to verify fragment size. The sonicated DNA was transferred from the Covaris tube to a 1.7 ml microfuge tube and the volume measured. The sonicated DNA was then diluted with TE buffer to 400 μl, heat-denatured for 10min at 95°C, then immediately cooled on ice for 10 min. Then 100μl of 5X IP buffer and 5μg of antibody (monoclonal mouse anti 5-methyl cytidine; Diagenode #C15200006) were added to the denatured (single-stranded) sonicated DNA. The DNA-antibody mixture was incubated overnight on a rotator at 4°C.

The following day magnetic beads (Dynabeads M-280 Sheep anti-Mouse IgG; Life Technologies 11201D) were pre-washed as follows: The beads were resuspended in the vial, then the appropriate volume (50 μl per sample) was transferred to a microfuge tube and put into a magnetic rack. Supernatant was removed and discarded. The same volume of washing buffer (PBS with 0.1% BSA and 2mM EDTA) (at least 1 ml) was added and the bead sample was resuspended. The tube was then placed into the magnetic rack for 1–2 minutes and the supernatant discarded. The tube was removed from the magnetic rack and the washed beads were resuspended in the original bead volume using 1xIP buffer (50 mM sodium phosphate pH 7.0, 700 mM NaCI and 0.25% triton-X-100) as the initial volume of beads. The 50μl of beads were added to 500μl of DNA-antibody mixture from the overnight incubation, then incubated for 2h on a rotator at 4°C.

After the incubation, the bead-DNA-antibody samples were washed three times with 1X IP buffer. The tube was placed into magnetic rack for 1–2 minutes and the supernatant discarded, then washed with 1xIP buffer 3 times. The washed samples were then resuspended in 250μl digestion buffer (5mM Tris PH8, 10.mM EDT4, 0.5% SDS) with 3.5μl Proteinase K (20mg/ml) and incubated for 2–3 hours on a rotator at 55°C. Then 250μl of buffered Phenol-Chloroform-Isoamylalcohol solution were added to the sample and the tube vortexed for 30 sec then centrifuged at 14,000rpm for 5min at room temperature. The aqueous supernatant was carefully removed and transferred to a fresh microfuge tube. Then 250μl chloroform were added to the supernatant from the previous step, vortexed for 30sec and centrifuged at 14,000rpm for 5min at room temperature. The aqueous supernatant was removed and transferred to a fresh microfuge tube. To the supernatant 2μl of glycoblue (20mg/ml), 20μl of 5M NaCl and 500μl ethanol were added and mixed well, then precipitated in -20°C freezer for >1 hour to overnight.

The DNA precipitate was centrifuged at 14,000rpm for 20min at 4°C and the supernatant removed, while not disturbing the pellet. The pellet was washed with 500μl cold 70% ethanol in -20°C freezer for 15 min. then centrifuged again at 14,000rpm for 5min at 4°C and the supernatant discarded. The tube was spun again briefly to collect residual ethanol at bottom of tube and then as much liquid as possible was removed with gel loading tip. Pellet was air-dried at RT until it looked dry (about 5 minutes) then resuspended in 20μl H_2_O or TE. DNA concentration was measured in Qubit (Life Technologies) with ssDNA kit (Molecular Probes Q10212).

### MeDIP-Seq analysis

The MeDIP DNA was used to create libraries for next generation sequencing (NGS) using the NEBNext® Ultra™ RNA Library Prep Kit for Illumina® (San Diego, CA) starting at step 1.4 of the manufacturer’s protocol to generate double stranded DNA from the single-stranded DNA resulting from MeDIP. After this step the manufacturer’s protocol was followed. Each individual sample received a separate index primer. NGS was performed at WSU Spokane Genomics Core using the Illumina HiSeq 2500 with a PE50 application, with a read size of approximately 50 bp and approximately 30 million reads per sample. Eight libraries were run in one lane.

### Statistics and bioinformatics

The basic read quality was verified using summaries produced by the FastQC program. The new data was cleaned and filtered to remove adapters and low quality bases using Trimmomatic [63]. The reads for each MeDIP sample were mapped to the Rnor 6.0 rat genome using Bowtie2 [64] with default parameter options. The mapped read files were then converted to sorted BAM files using SAMtools [65]. To identify DMRs, the reference genome was broken into 100 bp windows. The MEDIPS R package [66] was used to calculate differential coverage between control and exposure sample groups. The edgeR p-value [67] was used to determine the relative difference between the two groups for each genomic window. Windows with an edgeR p-value less than an arbitrarily selected threshold were considered DMRs. The DMR edges were extended until no genomic window with an edgeR p-value less than 0.1 remained within 1000 bp of the DMR. CpG density and other information was then calculated for the DMR based on the reference genome.

DMRs were annotated using the biomaRt R package [68] to access the Ensembl database [69]. The genes that associated with DMR were then input into the KEGG pathway search [70, 71] to identify associated pathways. The DMR associated genes were manually then sorted into functional groups by consulting information provided by the DAVID [72], Panther [73], and Uniprot databases incorporated into an internal curated database (www.skinner.wsu.edu under genomic data). All molecular data has been deposited into the public database at NCBI (GEO # GSE113785) and R code computational tools available at GitHub (https://github.com/skinnerlab/MeDIP-seq) and www.skinner.wsu.edu.

## Supporting information

S1 Fig Behavioral analysis using the Elevated Plus Maze (EPM) (A–E), or the open field apparatus (F). Asterisks indicate statistical significance by two-tailed Student’s t-test, *p < .05 and **p<0.01.
(PDF)

S1 Table F1 control and F1 vinclozolin lineage males and females. Rat ID, puberty (early or late), testis, ovary, prostate, kidney, tumor, lean, obese, multiple diseases, and total disease presented.
(PDF)

S2 Table F2 control and F2 vinclozolin lineage males and females. Rat ID, puberty (early or late), testis, ovary, prostate, kidney, tumor, lean, obese, multiple diseases, and total disease presented.
(PDF)

S3 Table F3 control and F3 vinclozolin lineage males and females. Rat ID, puberty (early or late), testis, ovary, prostate, kidney, tumor, lean, obese, multiple diseases, and total disease presented.
(PDF)

S4 Table Testis disease DMR signature list. DMR name, chromosome, start, length, number of signature windows, minimum p-value, CpG number, CpG density, maximum log fold change, annotation, gene and functional category presented.
(PDF)

S5 Table Prostate disease DMR signature list. DMR name, chromosome, start, length, number of signature windows, minimum p-value, CpG number, CpG density, maximum log fold change, annotation, gene and functional category presented.
(PDF)

S6 Table Kidney disease DMR signature list. DMR name, chromosome, start, length, number of signature windows, minimum p-value, CpG number, CpG density, maximum log fold change, annotation, gene and functional category presented.
(PDF)

S7 Table Multiple diseases DMR signature list. DMR name, chromosome, start, length, number of signature windows, minimum p-value, CpG number, CpG density, maximum log fold change, annotation, gene and functional category presented.
(PDF)
