# Supplementary figures and images for: Vinclozolin induced epigenetic transgenerational inheritance of pathologies and sperm epimutation biomarkers for specific diseases
Source: PLoS One. 2018 Aug 29;13(8):e0202662. doi: 10.1371/journal.pone.0202662 (PMC6114855; doi:10.1371/journal.pone.0202662)

Supplemental Figure S1

A

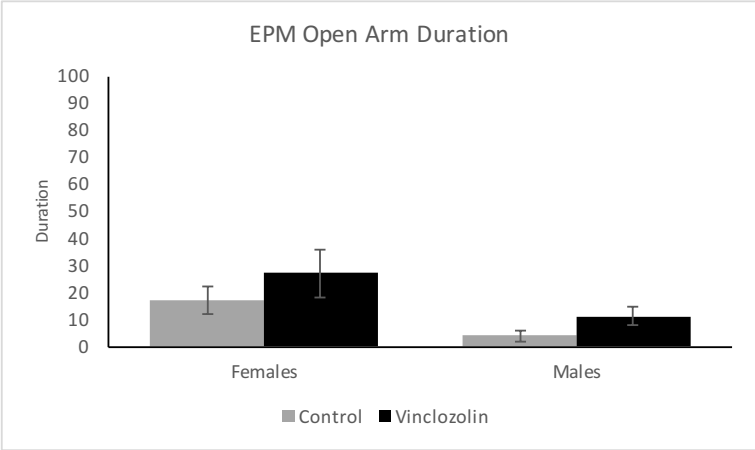

B

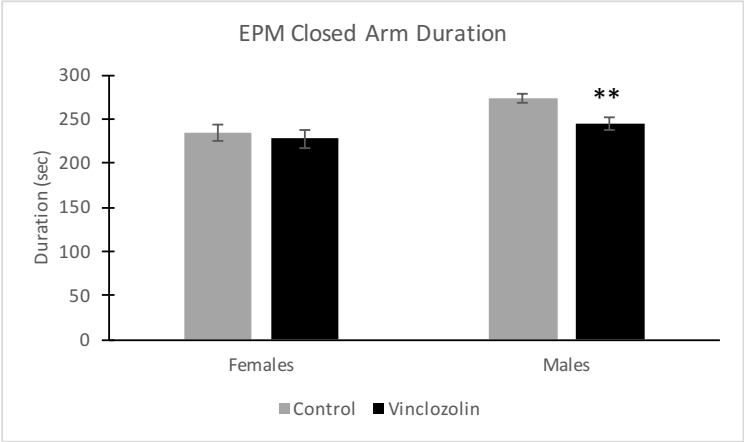

C

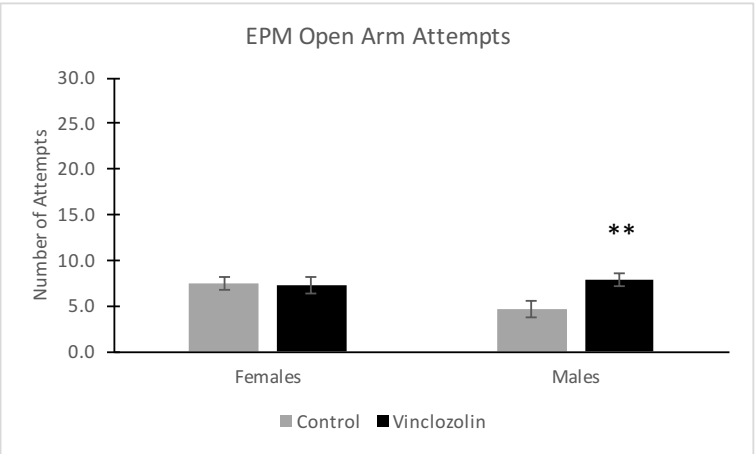

D

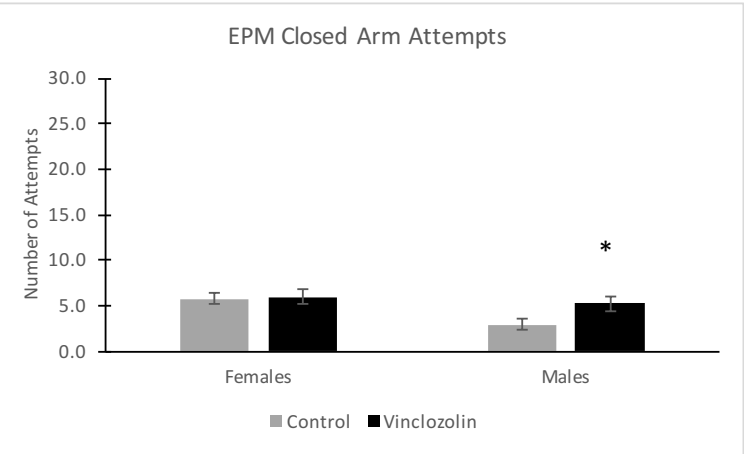

E

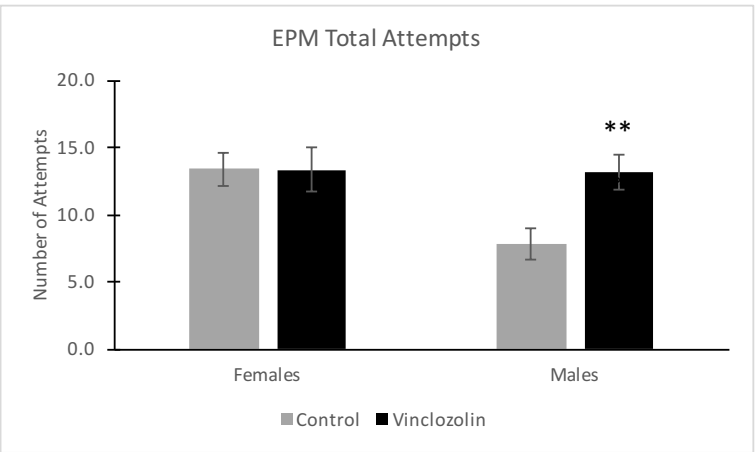

F

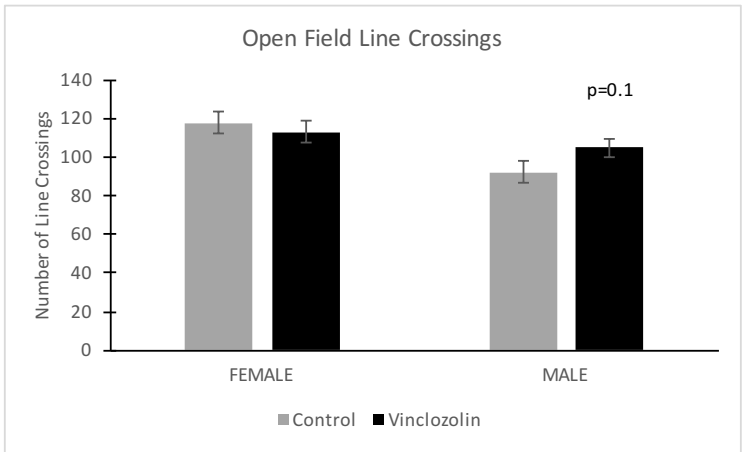

Supplement: S1 Fig — Behavioral analysis using the Elevated Plus Maze (EPM) (A–E), or the open field apparatus (F). Asterisks indicate statistical significance by two-tailed Student’s t-test, *p < .05 and **p<0.01. (PDF) [file pone.0202662.s001.pdf]
