# Supplementary material for: Vinclozolin induced epigenetic transgenerational inheritance of pathologies and sperm epimutation biomarkers for specific diseases
Source: PLoS One. 2018 Aug 29;13(8):e0202662. doi: 10.1371/journal.pone.0202662 (PMC6114855; doi:10.1371/journal.pone.0202662)
Supplement: S1 Table — Rat ID, puberty (early or late), testis, ovary, prostate, kidney, tumor, lean, obese, multiple diseases, and total disease presented. (PDF) [file pone.0202662.s002.pdf]

Supplemental Table S1

F1 Control Males										
	Puberty		Testis	Prostate	Kidney	Tumor	Lean	Obese	Multiple	Total
Rat ID	Early	Late							Disease	Disease
14C10-1-10-7	-	-	-	-	-	-	-	+	-	1
14C10-1-10-8	-	-	-	-	-	-	-	+	-	1
14C10-1-10-9	-	-	+	-	-	-	-	+	+	2
14C10-1-10-10	-	-	-	-	-	-	-	-	-	
14C12-1-3-5	-	-				-	-	+	-	1
14C16-1-6-6	-	-				-	-	-	-	
14C17-1-17-4	-	-				-	-	-	-	
14C17-1-17-5	-	-				-	-	-	-	
14C18-1-18-3	-	-				-	-	-	-	
14C19-1-19-8	-	-				-	-	-	-	
14C19-1-19-9	-	-				-	-	-	-	
14C19-1-19-10	-	-				-	+	-	-	1
14C19-1-19-11	-	-				-	-	-	-	
14C20-1-20-4						-	-	-	-	
14C20-1-20-5						-	-	-	-	
14C20-1-20-6						-	-	-	-	
AC2-1-2-1	+	-	+	-	+	-	-	-	+	3
AC2-1-2-2	-	-	-	-	-	-	-	-	-	
AC2-1-2-3	-	-	-	-	+	-	-	-	-	1
AC2-1-2-4	-	-	-		-	-	-	-	-	
AC2-1-2-5	-	-	-	-	-	-	-	-	-	
AC3-1-3-1						-	-	+	-	1
AC4-1-4-1	-	-				-	-	+	-	1
AC4-1-4-2	-	-				-	-	-	-	
AC4-1-4-3	-	-	-			-	-	-	-	
AC4-1-4-4	-	-				-	-	-	-	
AC6-1-6-1	-	-	-	-	-	-	-	-	-	
AC6-1-6-2	-	-		+	+	-	-	-	+	2
AC6-1-6-3	-	-	-	-	-	-	-	+	-	1
AC6-1-6-4	-	-	-	+	+	-	+	-	+	3
AC9-1-4-7	-	-	-	-	-	-	-	-	-	
AC9-1-4-8	-	-	-		-	-	-	-	-	
AC9-1-4-9	-	-				-			-	
**Affected**	1	0	2	2	4	0	1	7	4	
**Population**	29	29	15	13	15	33	32	32	33	

F1 Vinclozolin Males										
	Puberty		Testis	Prostate	Kidney	Tumor	Lean	Obese	Multiple	Total
Rat ID	Early	Late							Disease	Disease
14V10-1-10-10	-	-				-			-	
14V10-1-10-11	-	-				-			-	
14V10-1-10-9	-	-				-			-	
14V11-1-11-1						-			-	
14V11-1-11-4	-	-	-	-	-	-	-	+	-	1
14V1-1-1-5	-	-	-	-	+	-	+	-	+	2
14V16-1-16-9						-	-	+	-	1
14V2-1-2-8	-	-	+	-	-	-	-	-	-	1
14V3-1-3-4	-	-	-	-	+	-	-	+	+	2
14V4-1-4-1	-	-			-	-	-	-	-	
14V4-1-4-2	-	-		+	-	-	-	-	-	1
14V4-1-4-3				-						
14V5-1-5-8	-	-				+			-	1
14V5-1-5-9	-	-	-	-	+	-			-	1
14V5-1-5-10	-	-	-	-	-	-	-	-	-	
14V6-1-6-4	-	-				-	+	-	-	1
14V6-1-6-5	-	-				-	-	-	-	
14V6-1-6-6	-	-	-	-	-	-	-	+	-	1
14V6-1-6-7	-	-	+	+	-	-	-	-	+	2
14V6-1-6-8	-	-	+	-	-	-	-	-	-	1
15T1-1-1-8	-	-	+	+	+	-			+	3
15T2-1-2-9	-	-	+	-	-	-			-	1
15T2-1-2-10	-	-	-	+	-	-			-	1
15T6-1-6-7	-	-	-	-	-	-			-	
15T7-1-7-4	-	-	-	-	+	-			-	1
15T7-1-7-5	-	-	-	-	-	-			-	
**Affected**	**0**	**0**	**5**	**4**	**5**	**1**	**3**	**4**	**4**	
**Population**	**24**	**24**	**15**	**16**	**17**	**25**	**13**	**13**	**25**	

F1 Control Females									
	**Puberty**		**Ovary**	**Kidney**	**Tumor**	**Lean**	**Obese**	**Multiple Disease**	**Total Disease**
**Rat ID**	**Early**	**Late**							
14C10-1-10-1	-	-	-	-	-	+	-	-	**1**
14C10-1-10-2	-	-	-	-	-	-	+	-	**1**
14C12-1-3-1	-	-		-	-	+	-	-	**1**
14C12-1-3-2	-	-	-	+	-	-	-	-	**1**
14C12-1-3-3	-	-	-	-	-	-	-	-	
14C14-1-4-1			+		-	-	-	-	**1**
14C14-1-4-2	-	-	-		-	-	-	-	
14C16-1-6-1	-	-	-		-	-	-	-	
14C16-1-6-2	-	-	-		-	-	-	-	
14C16-1-6-3	-	-	+		-	-	-	-	
14C16-1-6-4	-	-	-		-	-	+	-	**1**
14C17-1-17-1	-	-	-		-	-	-	-	
14C17-1-17-2	-	-	-		-	-	+	-	**1**
14C17-1-17-3	-	-	-		-	-	+	-	**1**
14C18-1-18-1	-	-	-		-	-	+	-	**1**
14C18-1-18-2	-	-	-		-	-	+	-	**1**
14C19-1-19-1					-	+	-	-	**1**
14C19-1-19-2					-	-	+	-	**1**
14C19-1-19-3					-	-	-	-	
14C19-1-19-4					-	-	+	-	**1**
14C19-1-19-5					-	-	-	-	
14C19-1-19-6					-	-	+	-	**1**
14C19-1-19-7					-	+	-	-	**1**
14C20-1-20-1					-	-	-	-	
14C20-1-20-2					-	-	-	-	
14C20-1-20-3					-	-	-	-	
AC2-1-2-6	-	-	+	-	-	-	-	-	**1**
AC2-1-2-7	-	-	-	-	-	+	-	-	**1**
AC2-1-2-8	-	-							
AC2-1-2-9	-	-			-	-	-	-	
AC2-1-2-10	-	-	-	-	-	-	+	-	**1**
AC3-1-3-1	-	+			-			-	**1**
AC3-1-3-2	-	-	-	-	-	-	+	-	**1**
AC3-1-3-3	-	-	-		-	-	+	-	**1**
AC3-1-3-4	-	-	-	-	-	-	+	-	**1**
AC3-1-3-5	-	-			-	-		-	
AC3-1-3-6	-	-	-	-	-	-	-	-	**1**
AC6-1-6-5			+	-	+	-	-	+	**2**
AC9-1-4-1	-	-	-	-	+	-	-	-	**1**
AC9-1-4-2			+	-	-	-	-	-	
AC9-1-4-3			-	-	-	-	-	-	
AC9-1-4-4			-	-	-	-	-	-	
**Affected**	**0**	**1**	**5**	**1**	**2**	**5**	**13**	**1**	
**Population**	**27**	**27**	**27**	**16**	**41**	**39**	**39**	**41**	

F1 Vinclozolin Females									
	**Puberty**		**Ovary**	**Kidney**	**Tumor**	**Lean**	**Obese**	**Multiple Disease**	**Total Disease**
**Rat ID**	**Early**	**Late**							
14V10-1-10-1	-	-			-				
14V10-1-10-2	-	-			-				
14V10-1-10-3	-	-			-				
14V10-1-10-4	-	-			-				
14V10-1-10-5	-	-			-				
14V10-1-10-6	-	-			-				
14V10-1-10-7	-	-			-				
14V10-1-10-8	-	-			-				
14V1-1-1-1	-	-	-	-	-	+	-	-	**1**
14V11-1-11-1	-	-			-	-	+	-	**1**
14V11-1-11-2	-	-			-	-	-	-	
14V11-1-11-3	-	-			-	-	-	-	
14V1-1-1-2	-	-	-	-	-	-	+	-	**1**
14V1-1-1-3	-	-	-	+	-	-	-	-	**1**
14V1-1-1-4	-	-	-	-	-	-	-	-	
14V13-1-13-1	-	-			-			-	
14V16-1-16-1	-	+			-	-	-	-	**1**
14V16-1-16-2	-	-			-	+	-	-	**1**
14V16-1-16-3	-	+			-	-	-	-	**1**
14V16-1-16-4	-	-			-	-	-	-	
14V16-1-16-5	-	-			-	-	-	-	
14V16-1-16-6	-	-			-	-	-	-	
14V2-1-2-2	-	-	-	-	-			-	
14V2-1-2-3	-	-	+	-	-			-	**1**
14V2-1-2-4	-	-	-	-	-			-	
14V2-1-2-5	-	-	-	-	-	-	-	-	
14V2-1-2-6	-	-			-	-	-	-	
14V2-1-2-7	-	-			-	-	-	-	
14V3-1-3-1	-	-	-	+	-	-	+	+	**2**
14V3-1-3-2	-	-	-	-	-	-	-	-	
14V3-1-3-3	-	-	-	-	-	-	+	-	**1**
14V5-1-5-1	-	-	-	-	-	-	-	-	
14V5-1-5-2	-	-	-	-	-	-	-	-	
14V5-1-5-3	-	-	-	-	-	-	-	-	
14V5-1-5-4	-	-	-	-	+	-	-	-	**1**
14V5-1-5-7	-	-			-	+	-	-	**1**
14V6-1-6-1	-	-			-	-	-	-	
14V6-1-6-2	-	-			-	-	-	-	
14V6-1-6-3	-	-			-	-	-	-	
14V7-1-7-1	-	-			-	-	-	-	
14V7-1-7-2	-	-			-	-	-	-	
14V7-1-7-4	-	-			-	-	+	-	**1**
14V7-1-7-5	-	-			-	+	-	-	**1**
**Affected**	**0**	**2**	**1**	**2**	**1**	**4**	**5**	**1**	
**Population**	**43**	**43**	**15**	**15**	**43**	**31**	**31**	**35**	
