# Supplementary material for: Vinclozolin induced epigenetic transgenerational inheritance of pathologies and sperm epimutation biomarkers for specific diseases
Source: PLoS One. 2018 Aug 29;13(8):e0202662. doi: 10.1371/journal.pone.0202662 (PMC6114855; doi:10.1371/journal.pone.0202662)
Supplement: S2 Table — Rat ID, puberty (early or late), testis, ovary, prostate, kidney, tumor, lean, obese, multiple diseases, and total disease presented. (PDF) [file pone.0202662.s003.pdf]

Supplemental Table S2

F2 Control Males										
	Puberty		Testis	Prostate	Kidney	Tumor	Lean	Obese	Multiple	Total
Rat ID	Early	Late							Disease	Disease
14C10-2-7-4	-	-	-	-		-	-	-	-	
14C10-2-7-5	-	-	+	-		-	-	-	-	
14C10-2-7-6	-	-								
14C10-2-7-7	-	-								
14C17-2-13-6	-	-	-	-	-	-	+	-	-	1
14C17-2-13-7	-	-				-	+	-	-	1
14C17-2-13-8			-	-	-	-	+	-	-	1
14C17-2-13-11	-	-								
14C17-2-13-12	-	-								
14C18-2-16-3						-	-	-	-	
14C18-2-16-4						-	-	-	-	
14C18-2-16-5						-	+	-	-	1
14C18-2-17-4						-	+	-	-	1
14C18-2-17-5						-	-	-	-	
14C19-2-17-6						-	-	-	-	
14C19-2-17-7						-	-	-	-	
14C19-2-17-8						-	-	-	-	
14C19-2-17-9						-	-	-	-	
14C19-2-17-10						-	-	-	-	
14C19-2-18-7						-	-	-	-	
14C19-2-18-8						-	-	-	-	
14C19-2-18-9						-	-	-	-	
14C19-2-19-3	-	-				-	-	-	-	
14C19-2-19-4	-	-				-	-	+	-	1
14C19-2-19-5						-	-	+	-	1
14C19-2-19-6						-	-	-	-	
14C20-2-20-10						-	+	-	-	1
14C20-2-20-11						-	+	-	-	1
14C20-2-20-12						-	-	-	-	
14C20-2-21-4	-	+				-	-	-	-	1
14C20-2-21-5						-	-	-	-	
14C20-2-21-6						-	-	-	-	
14C20-2-21-7	-	+				-	-	-	-	1
14C20-2-21-8						-	-	-	-	
14C20-2-21-9	-	+				-			-	1
14C20-2-22-7	-	-				-	-	-	-	
14C20-2-22-8	-	-				-	-	-	-	
14C20-2-22-9						-	-	-	-	
14C20-2-22-10	-	-				-	-	-	-	
14C20-2-22-11	-	-				-	-	+	-	1
14C20-2-22-12						-	-	-	-	
AC2-2-1-6			+	+	-	-	-	+	+	3
AC2-2-1-7			+	-	-	-	+	-	+	2
AC2-2-3-3	-	-	-	-	-	-	-	-	-	
AC2-2-10-5	-	-	-	-	-	-	-	-	-	
AC2-2-10-6	-	-	-	-	-	-	+	-	-	1
AC2-2-10-7	-	-								
AC2-2-10-8	-	-								
AC6-2-2-4			-	-	-	-	-	-	-	
AC6-2-2-5			-	+	-	-	-	-	-	1
AC6-2-2-6	-	-	-	+	-	-	-	+	+	2
AC6-2-12-6	-	-	-	-	-	+	-	-	-	1
AC6-2-12-7	-	-	-	-	-	-	-	-	-	
AC6-2-12-8	-	-	-	+	-	-	-	-	-	1
AC9-2-6-4	-	-	-	-	-	-	-	-	-	
AC9-2-6-5	-	-	-	-	+	-	-	-	-	
AC9-2-6-6	-	-								
AC9-2-6-7	-	-								
AC9-2-6-8	-	-								
AC9-2-11-3	-	-	-	-	-	-	-	+	-	1
**Affected**	0	3	3	4	1	1	9	6	3	
**Population**	32	32	18	18	16	51	50	50	51	

F2 Vinclozolin Males										
	Puberty		Testis	Prostate	Kidney	Tumor	Lean	Obese	Multiple Disease	Total Disease
Rat ID	Early	Late								
14V1-2-2-5	-	-	-	-	-	-	-	-	-	
14V1-2-2-6	-	-	+	-	+	-	-	-	+	2
14V1-2-2-7	-	-	-	-	-	-	-	-	-	
14V2-2-3-2	-	-	-	+	-	-	-	+	+	2
14V2-2-3-3	-	-	-	-	+	-	-	-	-	1
14V3-2-4-5	+	-	-		-	-	-	-	-	1
14V3-2-4-6	+	-				-			-	1
14V6-2-5-10	-	-				-			-	
14V6-2-5-11	-	-	-	-	-	-	-	-	-	
14V6-2-5-12	-	-	-	-	-	-	-	-	-	
14V6-2-5-13	-	-	+	-	-	-	-	-	-	1
14V6-2-7-2	-	-	+	-	-	-	-	-	-	1
14V7-2-6-1	-	-	+	-	+	-	+	-	+	3
15V1-2-1-10	-	-	+	-	-	-	-	-	-	1
15V1-2-1-5	-	-	+	-	-	-	-	-	-	1
15V1-2-1-6	-	-	-	-	-	-	-	-	-	
15V1-2-1-7	-	-	+	-	-	-	-	-	-	1
15V1-2-1-8	-	-	-	+	-	-	-	-	-	1
15V1-2-1-9	-	-	+	-	-	-	-	-	-	1
15V1-2-2-6	-	-	+	-	-	-	-	-	-	1
15V1-2-2-7	-	-	+	-	-	-	-	-	-	1
15V1-2-2-9	-	-	+	+	-	-	-	-	+	2
15V1-2-3-10	-	-	-	-	-	-	-	-	-	
15V1-2-3-9	-	-	-	+	-	-	-	-	-	1
15V14-2-3-10						-	-	+	-	1
15V14-2-3-6						-	-	+	-	1
15V14-2-3-7						-	-	-	-	
15V14-2-3-8						-	-	-	-	
15V14-2-3-9						-	-	+	-	1
15V15-2-4-10						-	-	+	-	1
15V15-2-4-5						-	+	-	-	1
15V15-2-4-6						-	-	-	-	
15V15-2-4-7						-	-	-	-	
15V15-2-4-8						-	-	-	-	
15V15-2-4-9						-	-	-	-	
15V15-2-5-10						-	-	+	-	1
15V15-2-5-5						-	-	+	-	1
15V15-2-5-6						-	-	-	-	
15V15-2-5-7						-	-	-	-	
15V15-2-5-8						-	-	-	-	
15V15-2-5-9						-	-	-	-	
15V25-2-9-10						-	-	+	-	1
15V25-2-9-6						-	-	+	-	1
15V25-2-9-7						-	+	-	-	1
15V25-2-9-8						-	-	+	-	1
15V25-2-9-9						-	-	-	-	
**Affected**	**2**	**0**	**11**	**4**	**3**	**0**	**3**	**10**	**4**	
**Population**	**33**	**33**	**22**	**21**	**22**	**46**	**43**	**43**	**46**	

F2 Control Females									
	**Puberty**		**Ovary**	**Kidney**	**Tumor**	**Lean**	**Obese**	**Multiple**	**Total**
**Rat ID**	**Early**	**Late**						**Disease**	**Disease**
14C10-2-7-1	-	-	-	-	-	-	-	-	
14C10-2-7-2	-	-	+	-	-	-	-	-	
14C10-2-7-3	-	-	-	-	-	-	-	-	
14C10-2-8-1	-	-		-	-	-	-	-	
14C10-2-8-2	-	-	+	-	-	-	-	-	1
14C10-2-8-3	-	-	-	-	-	-	+	-	1
14C10-2-8-4	-	-			-	-	-	-	
14C17-2-13-1	-	-							
14C17-2-13-2	-	-			-	-	-	-	
14C17-2-13-3	-	-			-	-	-	-	
14C17-2-13-4	-	-			-	+	-	-	1
14C17-2-13-5	-	-			-	+	-	-	1
14C18-2-16-1					-	-	-	-	
14C18-2-17-1	-	-			-	-	-	-	
14C18-2-17-2	-	-			-	+	-	-	1
14C18-2-17-3	-	-			-	-	-	-	
14C18-2-17-4	-	-							
14C18-2-17-5	-	-							
14C19-2-19-2	-	-							
14C20-2-20-1	-	-			-	-	-	-	
14C20-2-20-3	-	-							
14C20-2-20-4					-	-	-	-	
14C20-2-20-5	-	-			-	-	-	-	
14C20-2-20-6	-	-			-	-	-	-	
14C20-2-20-7	-	-							
14C20-2-20-9	-	-			-	-	-	-	
14C20-2-21-1	-	-			-	-	-	-	
14C20-2-21-3	-	-			-	-	-	-	
14C20-2-22-1	-	-			-	-	-	-	
14C20-2-22-2	-	-							
14C20-2-22-3	-	-			-	-	-	-	
14C20-2-22-4					-	-	-	-	
14C20-2-22-6					-	-	-	-	
AC2-2-1-1			-	+	-	-	-	-	1
AC2-2-1-2	-	-			-	-	-	-	
AC2-2-1-3	-	-	-	-	-	-	-	-	
AC2-2-1-4	-	-	-	-	-	-	+	-	1
AC2-2-1-5	-	-			-	-	+	-	1
AC2-2-3-1	-	-	-	-	-	-	+	-	1
AC2-2-3-2	-	-	-	-	-	-	-	-	
AC2-2-5-1	-	-			-	-	-	-	
AC2-2-10-1					-	-	+	-	1
AC2-2-10-2	-	-			-	-	+	-	1
AC2-2-10-3	-	-			-	-	-	-	
AC2-2-10-4					-	-	-	-	
AC6-2-2-1	-	-	+	-	-	-	-	-	1
AC6-2-2-2	-	-	-	-	-	-	-	-	
AC6-2-2-3	-	-	-	+	-	-	-	+	1
AC6-2-12-1	-	-	-	-	-	-	-	-	
AC6-2-12-2	-	-	-	-	-	-	-	-	
AC6-2-12-3			+	-	-	-	-	-	1
AC6-2-12-4			-		-	-	-	-	
AC6-2-12-5					+			-	1
AC9-2-6-1			+	+	-	-	-	+	2
AC9-2-6-2	-	-	-	-	-	+	-	-	1
AC9-2-6-3	-	-	-	-	-	-	+	-	1
AC9-2-11-1	-	-	+	-	-	-	-	-	1
AC9-2-11-2	-	-	-	-	-	-	-	-	
**Affected**	**0**	**0**	**6**	**3**	**1**	**4**	**7**	**1**	
**Population**	**47**	**47**	**22**	**22**	**51**	**50**	**50**	**51**	

F2 Vinclozolin Females									
	Puberty		Ovary	Kidney	Tumor	Lean	Obese	Multiple	Total
Rat ID	Early	Late						Disease	Disease
14V1-2-2-1	-	-	-	+	-	-	-	-	1
14V1-2-2-2	-	-	+	-	-	-	-	-	1
14V1-2-2-3	-	-	-	-	-	-	-	-	
14V1-2-2-4	-	-			-	-	-	-	
14V2-2-3-1	-	-	-	-	-	-	+	-	1
14V3-2-4-1	-	-	-	-	-	-	+	-	1
14V3-2-4-2	-	-	-	+	-	-	+	+	2
14V3-2-4-3	-	-	-	-	-	-	-	-	
14V6-2-5-1	-	-	-	-	+	+	-	+	2
14V6-2-5-2	-	-	-	-	+	-	-	-	1
14V6-2-5-3	-	-	-	-	-	-	-	-	1
14V6-2-5-4	-	-	-	-	-	+	-	-	1
14V6-2-5-5	-	-			-	+	-	-	1
14V6-2-5-6	-	-			-	+	-	-	1
14V6-2-5-7	-	-			-				
14V6-2-5-8	-	-			-				
14V6-2-7-1	-	-	-	-	-	-	-	-	
15V1-2-1-1	-	-	-	-	-	-	-	-	
15V1-2-1-2	-	-	-	-	-	-	-	-	1
15V1-2-1-3	-	-	-	-	-	-	-	-	1
15V1-2-1-4	-	-	-	-	-	-	-	-	
15V1-2-2-1	-	-			+			-	1
15V1-2-2-2	-	-	-	-	-	-	-	-	
15V1-2-2-3	-	-	-	-	-	-	+	-	1
15V1-2-2-4	-	-	-	-	-	-	-	-	
15V1-2-2-5	-	-	-	-	-	-	-	-	
15V1-2-3-1	-	-	+	-	-	-	-	-	1
15V1-2-3-2	-	-	+	-	-	-	-	-	1
15V1-2-3-3	-	-	+	-	-	-	-	-	1
15V1-2-3-4	-	-	-	-	-	-	-	-	1
15V1-2-3-5	-	-	-	-	-	-	-	-	
15V1-2-3-6	-	-			-				
15V1-2-3-7	-	-			-				
15V14-2-3-2					-	-	+	-	1
15V14-2-3-3					-	-	-	-	
15V14-2-3-4					-	-	-	-	
15V14-2-3-5					-	-	+	-	1
15V15-2-4-2					-	+	-	-	1
15V15-2-4-4					-	-	-	-	
15V15-2-5-2					-	-	-	-	
15V15-2-5-4					-	-	+	-	1
15V2-2-4-1	-	-			-	-	-	-	
15V2-2-4-2	-	-			-	-	-	-	
15V2-2-4-3	-	-			-	+	-	-	1
15V2-2-4-4	+	-			-	+	-	+	2
15V2-2-4-5	-	-			-	+	-	-	1
15V2-2-4-6	-	-			-	-	-	-	
15V25-2-9-1					-	-	+	-	1
15V25-2-9-2					-	-	+	-	1
15V25-2-9-3					-	-	+	-	1
15V25-2-9-4					-	-	+	-	1
15V25-2-9-5					-	-	+	-	1
**Affected**	1	0	4	2	3	8	13	3	
**Population**	39	39	25	25	52	48	48	48	
