# Supplementary material for: Vinclozolin induced epigenetic transgenerational inheritance of pathologies and sperm epimutation biomarkers for specific diseases
Source: PLoS One. 2018 Aug 29;13(8):e0202662. doi: 10.1371/journal.pone.0202662 (PMC6114855; doi:10.1371/journal.pone.0202662)
Supplement: S3 Table — Rat ID, puberty (early or late), testis, ovary, prostate, kidney, tumor, lean, obese, multiple diseases, and total disease presented. (PDF) [file pone.0202662.s004.pdf]

Supplemental Table S3

F3 Control Males										
	Puberty		Testis	Prostate	Kidney	Tumor	Lean	Obese	Multiple	Total
Rat ID	Early	Late							Disease	Disease
14C10-3-2-5	-	-							-	
14C10-3-2-6	-	-							-	
14C10-3-2-7	-	-							-	
14C10-3-2-8	-	-	-	-	-	-	-	-	-	
14C10-3-2-9	-	-	-	-	-	-	-	-	-	
14C10-3-2-10	-	-	-	-	-	-	-	-	-	
14C10-3-3-6	-	-							-	
14C10-3-3-7	-	-							-	
14C10-3-3-8	-	-	-	-	-	-	-	-	-	
14C10-3-3-9	-	-	-	-	+	-	+	-	+	2
14C10-3-3-10	-	-	-	-	-	-	-	-	-	
14C17-3-4-7			-	-	-	-	+	-	-	1
14C17-3-4-8			-	-	-	-	+	-	-	1
14C17-3-4-9			-	-	-	-	+	-	-	1
14C17-3-4-10						-	-	-	-	
14C17-3-5-5			-	-	-	-	+	-	-	1
14C17-3-5-6			-	-	-	-	-	-	-	
14C17-3-5-7			+	+	-	-	+	-	+	3
14C17-3-5-9						-	-	+	-	1
14C17-3-5-13						-	-	-	-	
14C17-3-5-14						-	-	-	-	
14C17-3-6-9			-	-	-	-	-	-	-	
14C17-3-6-10			+	-	-	-	-	-	-	1
AC2-3-3-7	-	-							-	
AC2-3-3-8	-	-							-	
AC2-3-3-9	-	-	-	-	-	-	-	-	-	
AC2-3-3-10	-	-	-	-	-	-	-	-	-	
AC6-3-1-2	-	+							-	1
AC6-3-1-3	-	+							-	1
AC6-3-1-4	-	+	-	-	-	-	-	-	-	1
AC6-3-1-5	-	+	-	-	-	-	-	-	-	1
AC6-3-1-6	-	-	-	-	-	-	-	+	-	1
AC6-3-6-6	-	-							-	
AC6-3-6-7	-	-				-			-	
AC6-3-6-8	-	-	-	-	-	-	-	-	-	
AC6-3-6-9	-	-	-	-	+	-	-	-	-	1
AC9-3-4-6	-	-							-	
AC9-3-4-7	-	-							-	
AC9-3-4-8	-	-	-	-	-	-	-	-	-	
AC9-3-4-9	-	-	-	-	-	-	-	-	-	
AC9-3-4-10			-	-	-	-	-	-	-	
AC9-3-5-5	-	-							-	
AC9-3-5-6	-	-							-	
AC9-3-5-7	-	-							-	
AC9-3-5-8	-	-	-	-	-	-	-	+	-	1
AC9-3-5-9	-	-	-	-	-	-	-	-	-	
**Affected**	**0**	**4**	**2**	**1**	**2**	**0**	**6**	**3**	**2**	
**Population**	**33**	**33**	**26**	**26**	**26**	**31**	**30**	**30**	**46**	

F3 Vinclozolin Males										
	**Puberty**		**Testis**	**Prostate**	**Kidney**	**Tumor**	**Lean**	**Obese**	**Multiple**	**Total**
**Rat ID**	**Early**	**Late**							**Disease**	**Disease**
14V1-3-1-3	-	-	+	+	-	-	-	+	+	3
14V1-3-1-4	-	-	-	-	+	-	-	-	-	1
14V1-3-4-5	-	-	-	+	+	-	-	-	+	2
14V1-3-4-6	-	-	-	-	+	-	+	-	+	2
14V1-3-4-7	-	-	+	-	-	-	-	-	-	1
14V1-3-4-8	-	-				-			-	
14V1-3-4-9	-	-				-			-	
14V1-3-4-10	-	-				-			-	
14V1-3-4-11	-	-				-			-	
14V1-3-4-12	-	-				-			-	
14V2-3-3-7	-	-	-	-	+	-	-	+	+	2
14V2-3-3-8	-	-	+	+	-	-	-	-	+	2
14V2-3-3-9	-	-				-			-	
14V2-3-3-10	-	-				-			-	
14V3-3-2-5	-	-	-	+	-	-	-	+	+	2
14V3-3-2-7	-	-				-			-	
14V3-3-2-6	-	-	-	-	-	-	-	-	-	
14V3-3-2-8	-	-				-			-	
14V3-3-8-6	-	-	-	-	+	-	+	-	+	2
14V3-3-8-7	-	-	+	-	+	-	-	-	+	2
14V3-3-8-8	-	-				-			-	
14V6-3-6-6	-	-	-	-	+	-	-	-	-	1
14V6-3-6-7	-	-	+	-	+	-	-	-	+	2
14V6-3-6-8	-	-				-			-	
14V6-3-7-2	-	-	+	-	+	-	+	-	+	3
14V6-3-7-3	-	-				-			-	
15V14-3-14-6	-	-	-	+	-	-	-	-	-	1
15V14-3-14-7	-	-	+	-	+	-	-	-	+	2
15V14-3-14-8	-	-	+	-	-	-	-	+	+	2
15V14-3-14-9	-	-	-	+	-	-	-	+	+	2
15V14-3-14-10	-	-				-			-	
15V14-3-14-11	-	-				-			-	
15V14-3-14-12	-	-				-			-	
15V14-3-14-13	-	-				-			-	
15V14-3-15-10	-	-	+	-	-	-	-	-	-	1
15V14-3-15-11	-	-	+	+	+	-	-	-	+	3
15V14-3-15-7	-	-	+	+	-	-	-	+	+	3
15V14-3-15-8			+	+	-	-	-	-	+	2
15V14-3-15-9	-	-	-	+	-	-	-	-	-	1
15V15-3-17-9			+	+		-	-	-	+	2
15V15-3-19-8	-	-				-			-	
15V15-3-19-7	-	-				-			-	
15V25-3-20-5	-	-	-	-	-	-	-	-	-	
15V25-3-20-6	-	-	-	+	+	-	-	+	+	3
15V25-3-20-7	-	-	-	+	+	-	-	-	+	2
15V25-3-20-9	-	+				+			+	2
**Affected**	0	1	13	13	13	1	3	7	20	
**Population**	44	44	27	27	26	46	27	27	46	

F3 Control Females									
	Puberty		Ovary	Kidney	Tumor	Lean	Obese	Multiple Disease	Total Disease
**Rat ID**	**Early**	**Late**							
14C10-3-2-1	-	-	-	+	-	-	-	-	1
14C10-3-2-2	-	-	-	-	-	-	-	-	
14C10-3-2-3	-	-	-	-	-	-	-	-	
14C10-3-2-4	-	-	-	-	-	-	-	-	
14C10-3-3-1	-	-	+	-	-	-	-	-	1
14C10-3-3-2	-	-	-	-	-	-	-	-	
14C10-3-3-3	-	-	-	-	-	-	-	-	
14C10-3-3-4	-	-	+	+	-	-	-	+	2
14C10-3-3-5	-	-			-	+	-	-	1
14C17-3-4-1					-	-	-	-	
14C17-3-4-2					-	-	-	-	
14C17-3-4-3					-	+	-	-	1
14C17-3-4-4					-	-	-	-	
14C17-3-4-5					-	-	-	-	
14C17-3-4-6					-	+	-	-	1
14C17-3-5-1					-	-	-	-	
14C17-3-5-2					-	-	-	-	
14C17-3-5-3					-	-	-	-	
14C17-3-5-4					-	-	-	-	
14C17-3-6-1					-	-	-	-	
14C17-3-6-2					-	-	-	-	
14C17-3-6-3					-	-	-	-	
14C17-3-6-4					-	-	-	-	
14C17-3-6-5					-	-	+	-	1
14C17-3-6-6					-	-	+	-	1
14C17-3-6-7					-	-	+	-	1
AC2-3-3-1	-	-	+	-	-	-	-	-	1
AC2-3-3-2	-	-	-	-	-	-	-	-	
AC2-3-3-3	-	-	+	-	-	-	-	-	1
AC2-3-3-4	-	-	-	-	-	-	-	-	
AC2-3-3-5	-	-	-	-	+	-	-	-	
AC2-3-3-6	-	-			-	-	-	-	
AC2-3-7-1	-	-	-	-	-	-	-	-	
AC2-3-7-2			-	-	-	-	-	-	
AC6-3-1-1	-	+						-	1
AC6-3-1-7	-	-	-	-	-	-	-	-	
AC6-3-1-8	-	-	-	-	-	-	-	-	
AC6-3-6-1			-	-	-	-	-	-	
AC6-3-6-2			+	-	-	-	-	-	1
AC6-3-6-3			-	-	-	-	-	-	
AC6-3-6-4			-	-	-	-	-	-	
AC6-3-6-5			-	-	-	-	-	-	
AC9-3-4-1	-	-	-	-	-	-	-	-	
AC9-3-4-2	-	-	-	-	-	-	-	-	
AC9-3-4-3	-	-	-	-	-	-	-	-	
AC9-3-4-4			-	-	-	+	-	-	1
AC9-3-4-5	-	-			-	-	-	-	
AC9-3-5-1	-	-	-	-	-	-	-	-	
AC9-3-5-2	-	-	-	-	-	-	-	-	
AC9-3-5-3	-	-	-	-	-	-	-	-	
AC9-3-5-4	-	-	-	-	-	-	-	-	
**Affected**	0	1	5	2	1	4	3	1	
**Population**	27	27	30	30	50	50	50	50	

F3 Vinclozolin Females									
	Puberty		Ovary	Kidney	Tumor	Lean	Obese	Multiple	Total
Rat ID	Early	Late						Disease	Disease
14V1-3-1-1	-	-	-	-	-	-	-	-	
14V1-3-1-2	-	-	-	+	-	-	+	+	2
14V1-3-4-1	-	-	-	-	-	-	-	-	
14V1-3-4-2	-	-	-	-	+	+	-	+	2
14V1-3-4-3	-	-	-	-	+	-	+	+	2
14V1-3-4-4	-	-	+	-	-	-	-	-	1
14V2-3-3-1	-	-	+	-	-	-	-	-	1
14V2-3-3-2	-	-	-	-	-	-	-	-	
14V2-3-3-3	-	-	-	-	-	-	+	-	1
14V2-3-3-4	-	-	-	+	-	-	+	+	2
14V2-3-3-5	-	-			-	-	-	-	
14V2-3-3-6	-	-			-	-	-	-	
14V3-3-2-1	-	-	-	-	-	-	-	-	
14V3-3-2-2	-	-	-	-	-	-	-	-	
14V3-3-2-3	-	-	-	-	-	-	-	-	
14V3-3-2-4	-	-	-	-	-	-	-	-	
14V3-3-8-1	-	-	+	+	-	-	-	+	2
14V3-3-8-2	-	-	+	+	-	+	-	+	3
14V3-3-8-3	-	-	+	-	-	+	-	+	2
14V3-3-8-4	-	-	+	-	-	+	-	+	2
14V3-3-8-5	-	-	+	+	-	+	-	+	3
14V6-3-6-1	-	-	-	+	-	-	-	-	1
14V6-3-6-2	-	-	-	+	-	-	-	-	1
14V6-3-6-3	-	-	+	-	-	-	-	-	1
14V6-3-6-4	-	-	+	-	+			+	2
14V6-3-6-5	-	-			-	-	-	-	
14V6-3-7-1	-	-	-	-	-	-	+	-	1
15V1-3-13-1	-	-			-	-	-	-	
15V1-3-13-2	-	-			-	-	-	-	
15V1-3-13-3	-	-			-	-	-	-	
15V14-3-14-1	-	-			-	-	+	-	1
15V14-3-14-2	-	-			-	-	+	-	1
15V14-3-14-3	-	-			-	-	-	-	
15V14-3-14-4	-	-			-	-	+	-	1
15V14-3-14-5	-	-			-	-	+	-	1
15V14-3-15-1	-	-			-	-	+	-	1
15V14-3-15-2	-	-			-	-	+	-	1
15V14-3-15-3	-	-			-	-	+	-	1
15V14-3-15-4	-	-			-	-	+	-	1
15V14-3-15-5	-	-			-	-	-	-	
15V14-3-15-6	-	-			-	-	-	-	
15V15-3-17-1	-	-			-	-	+	-	1
15V15-3-17-2	-	-			-	-	-	-	
15V15-3-17-3	-	-			-	-	+	-	1
15V15-3-17-4	-	-			-	+	-	-	1
15V15-3-17-5	-	-			-	+	-	-	1
15V15-3-17-6	-	-			-	-	-	-	
15V15-3-17-7	-	-			-	-	-	-	
15V15-3-17-8	-	-			-	-	-	-	
15V15-3-19-1	-	-			-	-	-	-	
15V15-3-19-2	-	-			-	+	-	-	1
15V15-3-19-3	-	-			-	+	-	-	1
15V15-3-19-4	-	-			-	-	-	-	
15V15-3-19-5	-	-			-	-	-	-	
15V15-3-19-6	-	-			-	-	-	-	

15V2-3-10-1					+			-	**1**
15V2-3-10-2					-	-	-	-	
15V2-3-10-3					-	+	-	-	**1**
15V2-3-10-4					-	-	-	-	
15V2-3-10-5					-	-	-	-	
15V2-3-9-1					-	-	-	-	
15V2-3-9-2					-	-	-	-	
15V25-3-20-1	-	-			-	-	-	-	
15V25-3-20-2	-	-			-	-	+	-	**1**
15V25-3-20-3	-	-			-	-	-	-	
15V25-3-20-4	-	-			-	-	-	-	
**Affected**	**0**	**0**	**9**	**7**	**4**	**10**	**16**	**10**	
**Population**	**59**	**59**	**24**	**24**	**66**	**64**	**64**	**66**	
