# Supplementary material for: Vinclozolin induced epigenetic transgenerational inheritance of pathologies and sperm epimutation biomarkers for specific diseases
Source: PLoS One. 2018 Aug 29;13(8):e0202662. doi: 10.1371/journal.pone.0202662 (PMC6114855; doi:10.1371/journal.pone.0202662)
Supplement: S4 Table — DMR name, chromosome, start, length, number of signature windows, minimum p-value, CpG number, CpG density, maximum log fold change, annotation, gene and functional category presented. (PDF) [file pone.0202662.s005.pdf]

Supplemental Table S4
Testis Disease DMR Signature List

DMR Name	Chr	Start	Length	# Sig Win	minP	Log Fold Change	CpG #	CpG Density	Gene Annotation	Gene Category
DMR1:36596901	1	36596901	300	1	8.38E-06	-1.14	5	1.666666667	AABR07001061.1	
DMR1:56920701	1	56920701	1200	1	4.89E-06	-1.91	12	1	Wdr27	
DMR1:77593601	1	77593601	200	1	8.72E-06	1.78	2	1		
DMR1:85699401	1	85699401	100	1	7.10E-06	-2.23	1	1		
DMR1:91600001	1	91600001	300	1	7.30E-07	-1.4	6	2	Rhpn2	Signaling
DMR1:116281301	1	116281301	1500	1	1.25E-06	-1.48	14	0.933333333		
DMR1:139286401	1	139286401	400	1	2.26E-06	-1.29	2	0.5		
DMR1:208812201	1	208812201	100	1	8.75E-06	-1.48	3	3		
DMR1:208961201	1	208961201	200	1	8.39E-06	1.39	0	0		
DMR1:240443901	1	240443901	200	2	3.17E-06	-1.86	1	0.5	Trpm3	Receptor
DMR1:249518201	1	249518201	500	1	5.38E-06	-1.44	5	1		
DMR1:250494901	1	250494901	900	1	6.19E-06	-0.76	10	1.111111111	A1cf	
DMR1:261805701	1	261805701	200	1	5.89E-07	-2.17	0	0	AABR07006873.1	
DMR2:11533701	2	11533701	700	1	5.37E-06	-1.66	2	0.285714286		
DMR2:12476301	2	12476301	600	1	8.95E-06	-1.75	6	1		
DMR2:36610601	2	36610601	1000	1	4.40E-06	-2	7	0.7		
DMR2:47039301	2	47039301	200	1	4.28E-06	-2.23	2	1	Itga2	Signaling
DMR2:59777601	2	59777601	300	1	9.35E-06	-1.63	2	0.666666667		
DMR2:75746901	2	75746901	100	1	6.58E-06	1.68	0	0		
DMR2:93558001	2	93558001	300	1	5.19E-06	2.04	9	3		
DMR2:111433501	2	111433501	200	1	7.51E-06	-0.86	0	0	Nlgn1	Signaling
DMR2:113738301	2	113738301	800	1	7.70E-06	-1.72	9	1.125	Pld1	Metabolism
DMR2:123564701	2	123564701	200	1	5.00E-07	-1.45	1	0.5	RGD1307100	
DMR2:142431001	2	142431001	200	1	2.01E-06	-1.75	3	1.5	Lhfpl6	
DMR2:205445101	2	205445101	300	1	1.73E-06	-1.31	5	1.666666667	Nr1h5;RF00003	
DMR2:232297401	2	232297401	1200	1	6.57E-06	-1.04	18	1.5		
DMR3:3379601	3	3379601	1400	1	4.50E-06	1.49	11	0.785714286	Camsap1;LOC108348142	
DMR3:14306001	3	14306001	200	2	8.76E-07	-2.51	2	1	Cntrl;AABR07051508.2	
DMR3:82286801	3	82286801	600	1	5.70E-06	1.42	8	1.333333333	RF00001	
DMR3:91502301	3	91502301	200	1	3.37E-06	-1.93	2	1		
DMR3:93137901	3	93137901	1200	1	9.45E-06	-1.18	7	0.583333333		
DMR3:117883201	3	117883201	1100	1	9.84E-06	-2	16	1.454545455		
DMR3:124203301	3	124203301	500	1	1.93E-06	1.87	2	0.4		
DMR3:153369001	3	153369001	800	1	1.84E-06	-2.39	13	1.625	Mroh8	
DMR3:166123501	3	166123501	300	1	1.38E-07	-1.82	11	3.666666667		
DMR4:14860501	4	14860501	1300	1	9.05E-06	-1.14	4	0.307692308		
DMR4:27302301	4	27302301	200	1	6.68E-06	-1.97	0	0	Akap9	Signaling
DMR4:77943301	4	77943301	1800	1	6.67E-06	-1.83	10	0.555555556		
DMR4:78635601	4	78635601	900	1	1.98E-06	-1.39	7	0.777777778	Svs1	
DMR4:107700501	4	107700501	1000	1	5.76E-06	-1.42	4	0.4		
DMR4:123349601	4	123349601	200	1	3.59E-07	-1.41	2	1	Iqsec1	Signaling
DMR4:144603601	4	144603601	300	1	4.77E-06	-1.71	4	1.333333333	Rad18	Transcription
DMR5:2353201	5	2353201	200	1	9.84E-06	-2	3	1.5	AABR07046707.1	
DMR5:57262501	5	57262501	300	1	4.81E-07	-1.57	6	2	Bag1;Chmp5	Apoptosis;Binding Protein
DMR5:72038201	5	72038201	300	1	5.45E-06	-0.98	4	1.333333333		
DMR5:132977601	5	132977601	200	1	7.10E-06	-2.23	0	0		
DMR5:133063601	5	133063601	200	1	7.29E-06	-1.61	1	0.5	Skint10	
DMR5:140528201	5	140528201	500	1	1.00E-05	-1.24	10	2	Ppt1	Metabolism
DMR5:147555701	5	147555701	1900	1	2.51E-06	-2.01	24	1.263157895	Zbtb8os;Zbtb8a	
DMR5:155861801	5	155861801	1100	1	6.11E-06	1.62	23	2.090909091	AABR07073181.1	
DMR5:155917701	5	155917701	900	1	7.72E-06	-1.88	14	1.555555556	Ldlrad2	Receptor
DMR6:21927001	6	21927001	200	1	8.77E-08	-1.55	3	1.5		
DMR6:52406301	6	52406301	300	1	3.49E-07	-1.66	5	1.666666667	Cdhr3	Extracellular Matrix
DMR6:93333801	6	93333801	200	1	6.67E-06	-1.78	5	2.5	Frmd6	Signaling
DMR6:101225901	6	101225901	2000	1	2.73E-06	-1.08	21	1.05		
DMR6:109509901	6	109509901	1500	1	4.07E-06	-1.14	29	1.933333333	Jdp2	Transcription
DMR6:110109201	6	110109201	600	1	8.78E-06	-1.1	8	1.333333333	Gpatch2l	
DMR6:128963001	6	128963001	600	1	2.73E-06	2.22	11	1.833333333		
DMR7:31507801	7	31507801	2300	1	5.03E-06	-1.23	37	1.608695652	Anks1b	Transcription
DMR7:35761101	7	35761101	200	1	5.15E-06	-2.02	7	3.5		

DMR7:61932101	7	61932101	1400	2	1.11E-07	-1.74	22	1.571428571		
DMR7:66665001	7	66665001	800	2	2.59E-07	-1.64	3	0.375	Usp15;Snora19	Proteolysis
DMR7:74600601	7	74600601	200	1	2.87E-06	1.09	1	0.5		
DMR7:85581701	7	85581701	400	1	9.53E-06	-1.08	2	0.5		
DMR7:100241601	7	100241601	1000	1	5.11E-06	1.64	12	1.2		
DMR7:102200001	7	102200001	200	1	7.17E-06	-1.77	1	0.5		
DMR7:110170001	7	110170001	1300	1	2.09E-08	-2.4	10	0.769230769	Khdrbs3	Transcription
DMR7:133934801	7	133934801	100	1	2.15E-06	-3.07	3	3	Pdzrn4	
DMR8:58901	8	58901	1000	1	1.65E-06	-1.54	5	0.5		
DMR8:35720601	8	35720601	200	1	1.18E-06	1.49	2	1		
DMR8:59142801	8	59142801	200	1	7.53E-06	-1.78	0	0	Cib2	Signaling
DMR8:94047101	8	94047101	200	1	3.17E-06	-2.47	4	2	Ube3d	Proteolysis
DMR8:104398301	8	104398301	1900	1	4.73E-06	-1.78	30	1.578947368	AABR07071199.2	
DMR8:117063901	8	117063901	100	1	5.46E-06	-2.4	2	2	Nicn1;Amt	Metabolism
DMR9:58051601	9	58051601	200	1	5.86E-06	-1.86	1	0.5		
DMR9:99856701	9	99856701	700	1	1.42E-07	-1.64	13	1.857142857		
DMR9:100630401	9	100630401	400	1	2.02E-06	-1.59	1	0.25	Sept2;Hdlbp	Growth factor;Metabolism
DMR9:113577801	9	113577801	100	1	5.49E-06	1.34	1	1	Ppp4r1;Ralbp1	Signaling
DMR9:116684201	9	116684201	800	2	4.77E-07	-2.17	10	1.25	AABR07068728.1	
DMR9:121519801	9	121519801	1400	1	4.10E-06	-1.78	6	0.428571429		
DMR10:23139801	10	23139801	400	1	1.46E-06	-2.71	4	1		
DMR10:27158001	10	27158001	500	1	7.29E-06	-1.36	7	1.4	Gabrg2	Receptor
DMR10:44622301	10	44622301	500	1	7.61E-06	-1.73	1	0.2	Olr1448	Receptor
DMR10:61838901	10	61838901	1100	1	3.40E-07	-1.64	5	0.454545455	Smg6	Transcription
DMR10:79953601	10	79953601	200	1	8.20E-06	1.97	0	0		
DMR10:84717401	10	84717401	600	1	1.45E-06	-2.33	10	1.666666667	AABR07072169.1;Copz2;Mir152	Cytoskeleton
DMR11:4294701	11	4294701	1700	1	5.43E-06	-1.41	15	0.882352941	Cadm2	Receptor
DMR11:11403101	11	11403101	200	1	2.16E-07	-2.63	2	1	Robo2	Receptor
DMR11:28047601	11	28047601	100	1	2.77E-06	-1.94	1	1	AABR07033570.2	
DMR11:45179501	11	45179501	300	1	1.02E-06	2.26	5	1.666666667		
DMR11:59664201	11	59664201	200	1	7.96E-07	-2.11	2	1		
DMR11:59857201	11	59857201	200	1	5.14E-06	-1.72	3	1.5		
DMR11:68598201	11	68598201	200	1	9.00E-06	-1.55	2	1	RF00003	
DMR11:80106501	11	80106501	200	1	4.73E-06	-1.81	1	0.5		
DMR12:7911901	12	7911901	300	1	9.33E-06	-1.01	4	1.333333333	Ubl3	Metabolism
DMR12:17745501	12	17745501	2700	1	4.96E-06	-1.55	45	1.666666667	Pdgfa	Growth factor
DMR12:28827201	12	28827201	900	1	3.30E-06	-2.21	9	1	AABR07035946.1	
DMR13:7796101	13	7796101	400	1	6.89E-06	-2.64	0	0		
DMR13:23129601	13	23129601	200	1	8.98E-06	1.47	2	1		
DMR13:24705301	13	24705301	100	1	2.99E-06	2.06	0	0		
DMR13:34339901	13	34339901	800	1	4.17E-06	-1.41	4	0.5		
DMR13:81371901	13	81371901	700	1	6.86E-06	2.14	9	1.285714286		
DMR13:105439601	13	105439601	1000	1	8.91E-06	-1.22	18	1.8		
DMR13:112772901	13	112772901	100	1	9.61E-06	-2.84	1	1		
DMR14:13482401	14	13482401	1500	1	3.16E-07	-1.27	5	0.333333333		
DMR14:13682601	14	13682601	400	1	4.68E-07	-1.28	0	0		
DMR14:76584001	14	76584001	200	1	1.12E-06	-2.72	3	1.5		
DMR14:86847801	14	86847801	200	1	4.50E-06	-1.09	5	2.5	Ccm2	Development
DMR15:53531701	15	53531701	300	1	3.83E-08	-1.85	5	1.666666667		
DMR15:71587501	15	71587501	300	1	5.59E-06	-1.34	1	0.333333333		
DMR15:79183301	15	79183301	300	1	4.20E-06	-1.8	0	0		
DMR15:87576201	15	87576201	100	1	5.95E-06	-2.05	0	0	Mycbp2	Metabolism
DMR15:99604601	15	99604601	200	1	8.89E-06	-0.93	2	1		
DMR15:101664001	15	101664001	200	1	5.08E-06	0.99	4	2		
DMR16:12727301	16	12727301	300	1	1.91E-06	-1.6	2	0.666666667	LOC100911649	Unknown
DMR16:23876101	16	23876101	300	1	7.62E-07	-1.49	4	1.333333333		
DMR16:27540301	16	27540301	200	1	2.47E-06	-1.58	2	1	Tll1	Protease
DMR16:32635201	16	32635201	500	1	2.55E-06	-1.93	18	3.6		
DMR16:59771901	16	59771901	1300	1	5.40E-06	-0.96	20	1.538461538		
DMR16:81133501	16	81133501	300	1	6.32E-06	-1.74	2	0.666666667	Tfdp1;Atp4b	Transcription;Transport
DMR17:3264101	17	3264101	600	1	7.74E-06	-0.86	3	0.5		
DMR17:5657701	17	5657701	1600	1	5.24E-06	-1.4	21	1.3125	AABR07026936.3	
DMR18:57662901	18	57662901	100	1	5.10E-06	2.18	2	2	Htr4	Receptor
DMR18:64867701	18	64867701	300	1	6.23E-06	1.75	6	2		

DMR19:6201401	19	6201401	600	1	7.53E-06	-1.73	1	0.166666667	Cdh8	Extracellular Matrix
DMR19:40304601	19	40304601	300	1	4.67E-06	-1.17	3	1		
DMR19:47603501	19	47603501	1200	2	1.83E-06	-2.54	23	1.916666667		
DMR19:57987501	19	57987501	500	1	3.48E-06	-1.69	13	2.6	Disc1	
DMR20:24565801	20	24565801	400	1	4.19E-06	-0.93	1	0.25		
DMR20:26805301	20	26805301	100	1	8.58E-06	-2.05	0	0	Herc4	Metabolism
DMRX:6835301	X	6835301	1300	1	1.83E-07	-1.2	11	0.846153846		
DMRX:39511001	X	39511001	2000	1	5.82E-06	-1.09	18	0.9		
DMRX:55150401	X	55150401	200	1	4.97E-06	-1.79	2	1		
DMRX:80396301	X	80396301	1700	1	8.48E-06	-1.45	21	1.235294118		
DMRX:118280701	X	118280701	200	1	6.95E-06	-1.66	2	1	Mir448;Htr2c	Receptor
DMRX:137758901	X	137758901	200	1	1.03E-06	-1.62	8	4		
