# Supplementary material for: Vinclozolin induced epigenetic transgenerational inheritance of pathologies and sperm epimutation biomarkers for specific diseases
Source: PLoS One. 2018 Aug 29;13(8):e0202662. doi: 10.1371/journal.pone.0202662 (PMC6114855; doi:10.1371/journal.pone.0202662)
Supplement: S5 Table — DMR name, chromosome, start, length, number of signature windows, minimum p-value, CpG number, CpG density, maximum log fold change, annotation, gene and functional category presented. (PDF) [file pone.0202662.s006.pdf]

Supplemental Table S5
Prostate Disease DMR Signature List

DMR Name	Chr	Start	Length	# Sig Win	minP	Log Fold Change	CpG #	CpG Density	Gene Annotation	Gene Category
DMR1:18191401	1	18191401	600	1	4.72E-06	-1.9	2	0.333333333		
DMR1:45719301	1	45719301	200	1	9.02E-06	2	3	1.5		
DMR1:51934101	1	51934101	1700	1	1.48E-06	-2.05	19	1.117647059		
DMR1:52097501	1	52097501	200	1	4.46E-06	-1.65	1	0.5		
DMR1:53191901	1	53191901	1800	1	9.64E-06	1.2	21	1.166666667	Rnaset2	Translation
DMR1:59254901	1	59254901	400	1	7.36E-06	-0.86	2	0.5		
DMR1:72617001	1	72617001	400	1	6.26E-07	-1.21	1	0.25	Rpl28;AABR07071876.1	Transcription
DMR1:81097101	1	81097101	2000	1	9.83E-06	1.95	35	1.75	AC118165.1	
DMR1:86584201	1	86584201	300	1	4.38E-06	-0.9	4	1.333333333		
DMR1:100540501	1	100540501	800	1	8.53E-07	1.11	14	1.75	Spib;Pold1	Transcription
DMR1:114757701	1	114757701	600	1	9.16E-06	-0.97	2	0.333333333	Oca2	Transport
DMR1:123245201	1	123245201	200	1	1.42E-06	-1.86	2	1		
DMR1:140130301	1	140130301	1300	1	1.48E-06	2.19	10	0.769230769	Ntrk3	Receptor
DMR1:140506501	1	140506501	1500	1	1.39E-06	-0.89	27	1.8		
DMR1:154446601	1	154446601	200	1	2.04E-06	-1.61	0	0	Picalm	Transport
DMR1:155532701	1	155532701	1500	1	8.97E-06	-1.28	3	0.2		
DMR1:160809501	1	160809501	100	1	3.88E-06	-2.36	1	1		
DMR1:161748001	1	161748001	400	1	4.60E-06	-0.91	6	1.5	Tenm4	Receptor
DMR1:172507701	1	172507701	2500	1	2.76E-06	-1.64	8	0.32	AC127217.1	
DMR1:177476401	1	177476401	300	1	8.37E-06	-1.35	8	2.666666667		
DMR1:178653201	1	178653201	400	1	6.44E-07	-1.1	3	0.75	Spon1	Growth factor
DMR1:179664501	1	179664501	2300	1	6.92E-06	-0.77	18	0.782608696		
DMR1:183452701	1	183452701	300	1	4.38E-06	-0.98	3	1		
DMR1:194047301	1	194047301	500	1	3.18E-06	-1.3	4	0.8		
DMR1:197371501	1	197371501	700	1	2.17E-07	-1.22	12	1.714285714		
DMR1:210215101	1	210215101	300	1	4.49E-07	1.53	1	0.333333333		
DMR1:218931901	1	218931901	100	1	1.22E-07	-2.66	1	1		
DMR1:241357601	1	241357601	3000	1	7.10E-06	-1.16	25	0.833333333	RGD1560242	
DMR1:247842701	1	247842701	200	1	7.71E-06	-1.53	2	1		
DMR1:263138401	1	263138401	300	1	7.99E-06	-1.54	5	1.666666667		
DMR1:263551801	1	263551801	300	1	8.90E-06	1.17	9	3	Abcc2	Transport
DMR2:7601701	2	7601701	300	1	7.21E-07	-1.24	1	0.333333333		
DMR2:24206101	2	24206101	200	1	5.46E-06	-1.73	3	1.5	Ap3b1;AABR07007690.1	Transport
DMR2:29833601	2	29833601	300	1	7.22E-06	-1.35	2	0.666666667		
DMR2:37567901	2	37567901	700	1	6.03E-06	-1	3	0.428571429		
DMR2:40711501	2	40711501	300	1	9.10E-06	-1.24	3	1	Pde4d	Metabolism
DMR2:50738801	2	50738801	400	1	6.27E-06	1.12	2	0.5		
DMR2:55980901	2	55980901	300	1	2.58E-07	-1.08	4	1.333333333	Fyb1	
DMR2:56528801	2	56528801	200	1	3.42E-06	-2.52	5	2.5	Egflam	
DMR2:90057501	2	90057501	800	1	1.34E-06	-0.55	2	0.25		
DMR2:105238101	2	105238101	400	1	8.64E-06	-0.8	4	1		
DMR2:108235501	2	108235501	300	1	6.92E-06	-1.26	4	1.333333333		
DMR2:112021301	2	112021301	400	1	2.49E-07	-1.17	1	0.25	AABR07009775.1	
DMR2:112783301	2	112783301	1800	1	8.30E-06	-0.88	18	1	Ect2;RF00560	Signaling
DMR2:116671201	2	116671201	1200	1	8.73E-06	-1.39	12	1		
DMR2:125172601	2	125172601	400	1	1.32E-06	-1.18	2	0.5		
DMR2:125906701	2	125906701	600	1	6.99E-06	-0.66	4	0.666666667		
DMR2:126061201	2	126061201	1300	1	6.12E-06	-0.88	7	0.538461538		
DMR2:135058701	2	135058701	200	1	9.89E-06	-1.15	0	0		
DMR2:137172501	2	137172501	200	1	3.56E-06	-1.19	2	1		
DMR2:145808101	2	145808101	500	1	1.39E-07	-0.7	2	0.4		
DMR2:148189401	2	148189401	200	1	6.22E-06	-0.82	2	1		
DMR2:170767401	2	170767401	200	1	2.62E-06	-1.21	3	1.5		
DMR2:181351301	2	181351301	200	1	7.16E-06	-1.24	2	1	Map9	
DMR2:184373201	2	184373201	400	1	5.26E-06	-0.97	5	1.25		
DMR2:209038101	2	209038101	1300	1	4.72E-06	-1.94	5	0.384615385		
DMR2:210587301	2	210587301	500	1	2.70E-06	-1.3	9	1.8		
DMR2:219230601	2	219230601	500	1	4.74E-06	-1.1	4	0.8		
DMR2:225886501	2	225886501	1000	1	7.69E-06	-1.31	10	1		

DMR2:228921901	2	228921901	400	1	6.82E-06	-1.19	1	0.25		
DMR2:235992301	2	235992301	600	1	6.71E-06	-0.61	14	2.333333333		
DMR2:236801901	2	236801901	300	1	5.30E-06	-0.98	6	2		
DMR2:241690501	2	241690501	800	1	4.61E-06	-0.93	6	0.75		
DMR2:242350601	2	242350601	200	1	5.86E-06	-1.37	2	1		
DMR2:247488901	2	247488901	200	1	1.75E-06	-2.32	5	2.5		
DMR2:257748901	2	257748901	1000	1	6.26E-06	-0.8	31	3.1	Ak5	Signaling
DMR3:586501	3	586501	300	1	9.54E-06	-2.03	2	0.666666667		
DMR3:4437701	3	4437701	300	1	9.30E-06	-1.4	2	0.666666667		
DMR3:10986901	3	10986901	1000	1	5.46E-06	-2.24	17	1.7		
DMR3:11496501	3	11496501	200	1	3.19E-06	-1.32	3	1.5		
DMR3:27814301	3	27814301	400	1	9.13E-06	-1.15	4	1		
DMR3:37809101	3	37809101	300	1	1.10E-06	1.32	9	3	Neb	Cytoskeleton
DMR3:38208201	3	38208201	300	1	3.60E-06	-2.32	3	1		
DMR3:51331801	3	51331801	400	1	3.64E-06	1.21	8	2		
DMR3:60927201	3	60927201	800	1	2.43E-06	-0.87	7	0.875		
DMR3:67683301	3	67683301	1400	1	9.80E-06	-1.21	18	1.285714286	AABR07052643.1	
DMR3:72799801	3	72799801	1300	1	9.78E-06	1.716	12	0.923076923	Olr441	
DMR3:83052201	3	83052201	1100	1	1.31E-06	-0.84	13	1.181818182	Hsd17b12;AABR0705289	Metabolism
DMR3:89871701	3	89871701	600	1	7.48E-06	-1.35	4	0.666666667		
DMR3:91919601	3	91919601	300	1	3.70E-06	-0.97	7	2.333333333		
DMR3:117758801	3	117758801	200	1	1.78E-07	-1.46	6	3	Fbn1;AABR07053635.1	Development
DMR3:124050701	3	124050701	600	1	3.72E-06	-0.68	2	0.333333333		
DMR3:124979001	3	124979001	1400	1	3.23E-06	-1.28	20	1.428571429		
DMR3:138848401	3	138848401	200	1	4.82E-06	-1.37	3	1.5	Dtd1	Transcription
DMR3:141217601	3	141217601	500	1	8.19E-06	-0.71	6	1.2	Xrn2	Transcription
DMR3:157687501	3	157687501	300	1	1.94E-06	-1.15	5	1.666666667	Ptprt	Receptor
DMR3:166298501	3	166298501	400	1	3.79E-06	-0.68	13	3.25	AABR07054662.2	
DMR3:171931601	3	171931601	1000	1	1.52E-06	1.38	12	1.2	AABR07054850.1	
DMR4:355401	4	355401	1200	1	1.47E-06	-0.56	10	0.833333333	Insig1	Signaling
DMR4:1902701	4	1902701	300	1	1.81E-07	-1.24	0	0		
DMR4:3300801	4	3300801	400	1	1.43E-06	-1.2	3	0.75		
DMR4:10715801	4	10715801	400	1	6.00E-06	-2.07	5	1.25		
DMR4:23807301	4	23807301	100	1	6.11E-06	1.71	2	2		
DMR4:29218101	4	29218101	1500	1	6.33E-06	2.04	6	0.4		
DMR4:36234401	4	36234401	300	1	4.46E-06	-1.73	1	0.333333333		
DMR4:40894001	4	40894001	400	1	8.88E-06	-0.67	5	1.25	RF00100	
DMR4:45715201	4	45715201	200	1	4.49E-06	-1.13	4	2		
DMR4:51231201	4	51231201	400	1	5.40E-06	-1.13	3	0.75		
DMR4:53101901	4	53101901	700	1	5.10E-06	-0.99	8	1.142857143		
DMR4:53847501	4	53847501	1300	1	1.35E-06	-0.85	7	0.538461538		
DMR4:61293201	4	61293201	400	1	2.01E-06	-1.6	2	0.5	Exoc4	Transport
DMR4:68874901	4	68874901	200	1	1.83E-06	-2.14	3	1.5	Mgam	Metabolism
DMR4:83554301	4	83554301	1600	1	7.93E-06	-1.78	13	0.8125	Creb5	Transcription
DMR4:86329401	4	86329401	1100	1	6.22E-06	-1.2	39	3.545454545		
DMR4:99664301	4	99664301	400	1	9.05E-06	-0.55	5	1.25	Reep1	Receptor
DMR4:101323901	4	101323901	600	2	5.05E-07	-1.04	9	1.5	AABR07060930.1	
DMR4:105976501	4	105976501	700	1	1.32E-06	1.75	2	0.285714286		
DMR4:130358601	4	130358601	500	1	1.55E-06	-0.99	5	1	Mitf	Transcription
DMR4:152691501	4	152691501	500	1	6.55E-06	-0.9	10	2	Ninj2	Extracellular Matrix
DMR4:164655901	4	164655901	1600	1	5.98E-06	-1.7	7	0.4375	Ly49s4;Ly49i4	Receptor
DMR4:167153701	4	167153701	300	1	4.71E-06	-1.44	1	0.333333333	Tas2r116;Tas2r123	
DMR4:176910501	4	176910501	1200	1	5.77E-06	-1.16	11	0.916666667	Abcc9	Receptor
DMR4:178696801	4	178696801	200	1	6.33E-06	1.31	3	1.5		
DMR4:182368201	4	182368201	500	1	5.20E-07	-0.74	2	0.4		
DMR5:11104501	5	11104501	200	1	5.24E-07	-0.9	11	5.5		
DMR5:11940801	5	11940801	400	1	4.74E-06	-0.9	1	0.25		
DMR5:14360301	5	14360301	300	1	2.67E-06	-1.21	2	0.666666667	Atp6v1h	Transport
DMR5:17194701	5	17194701	200	1	5.48E-06	1.76	1	0.5		
DMR5:18790301	5	18790301	1400	1	8.01E-06	-1.13	19	1.357142857		
DMR5:22263201	5	22263201	100	1	6.66E-06	-1.64	1	1	AABR07047174.3;AABR07047174.1	
DMR5:41454501	5	41454501	200	1	1.50E-06	-1.09	0	0		
DMR5:52380401	5	52380401	200	1	2.88E-06	-1.13	1	0.5		

DMR5:54544401	5	54544401	300	1	9.80E-06	-1.63	3	1		
DMR5:59352001	5	59352001	400	1	1.71E-06	-1.48	5	1.25	Reck	Signaling
DMR5:72853301	5	72853301	900	1	5.05E-06	-0.59	11	1.222222222		
DMR5:74677201	5	74677201	1200	1	1.32E-07	-2.13	16	1.333333333	Palm2	
DMR5:76060901	5	76060901	300	1	5.91E-06	-0.73	4	1.333333333		
DMR5:77388201	5	77388201	1200	1	4.72E-06	-1.22	6	0.5	LOC259244;Mup4;AABR0	Immune response
DMR5:118499401	5	118499401	300	1	2.88E-06	-1.1	2	0.666666667	Itgb3bp;RF00026	Receptor
DMR5:118717701	5	118717701	600	1	1.04E-06	-1.99	3	0.5	Efcab7	Metabolism
DMR5:122580201	5	122580201	100	1	1.35E-06	-2.35	1	1	Wdr78	Cytoskeleton
DMR5:131648601	5	131648601	1100	1	3.48E-06	1.82	8	0.727272727		
DMR5:135058801	5	135058801	1200	1	2.43E-06	-1.51	9	0.75		
DMR5:137685601	5	137685601	3100	1	8.43E-06	-1.59	33	1.064516129		
DMR5:146188901	5	146188901	1000	1	1.25E-07	-0.51	12	1.2		
DMR5:146771001	5	146771001	2100	1	9.32E-06	-0.68	29	1.380952381	Zscan20	Transcription
DMR5:150848901	5	150848901	300	1	1.73E-06	0.98	7	2.333333333	Eya3	Metabolism
DMR5:156474701	5	156474701	300	1	4.83E-06	-0.93	3	1	Eif4g3	Transcription
DMR5:158699401	5	158699401	700	1	8.68E-06	1.19	21	3	AABR07050265.1	
DMR5:166967901	5	166967901	600	1	2.40E-06	-1.11	9	1.5		
DMR5:167010301	5	167010301	700	1	2.98E-06	-1.19	13	1.857142857	H6pd	Metabolism
DMR5:170058001	5	170058001	1000	1	5.80E-06	-0.45	5	0.5		
DMR5:170857901	5	170857901	400	2	2.03E-06	-0.52	6	1.5		
DMR5:172398201	5	172398201	3200	1	1.19E-06	-0.67	51	1.59375	Plch2	Metabolism
DMR6:3008001	6	3008001	300	1	1.72E-07	-1.39	3	1	Arhgef33	
DMR6:4852601	6	4852601	300	1	7.51E-06	-0.78	3	1		
DMR6:10809101	6	10809101	1900	1	6.33E-06	-0.75	84	4.421052632		
DMR6:25884201	6	25884201	400	2	2.40E-07	-1.29	5	1.25	Babam2	
DMR6:45791701	6	45791701	2700	1	9.98E-06	-1.08	22	0.814814815		
DMR6:53581301	6	53581301	300	1	8.95E-06	-1.64	5	1.666666667	Hdac9	
DMR6:54022801	6	54022801	300	1	7.99E-06	-1.21	6	2	Hdac9	
DMR6:87207501	6	87207501	3300	1	3.62E-06	-1	30	0.909090909	RF00560	
DMR6:87641901	6	87641901	300	1	5.86E-06	-1.23	4	1.333333333		
DMR6:89070201	6	89070201	200	1	5.99E-06	-1.45	2	1		
DMR6:93651701	6	93651701	1200	1	3.09E-06	-1.5	16	1.333333333	AC128303.1;LOC690035	
DMR6:94796001	6	94796001	800	1	5.13E-06	-0.84	13	1.625		
DMR6:101081701	6	101081701	200	1	9.18E-06	-0.98	3	1.5		
DMR6:106690301	6	106690301	1200	1	3.74E-06	-1.25	15	1.25		
DMR6:109368001	6	109368001	1200	1	3.00E-06	1.72	17	1.416666667		
DMR6:128080301	6	128080301	1200	1	8.78E-06	1.29	20	1.666666667	LOC500712;RF00560	
DMR6:128236601	6	128236601	900	1	3.62E-06	-1.19	15	1.666666667		
DMR6:128867901	6	128867901	200	1	9.39E-07	-1	3	1.5		
DMR6:134522301	6	134522301	700	1	4.79E-06	1.69	14	2		
DMR7:11891601	7	11891601	400	1	4.78E-06	-1.03	3	0.75	Mob3a	
DMR7:14936001	7	14936001	200	1	6.88E-06	-1.28	1	0.5		
DMR7:20859201	7	20859201	700	1	5.67E-06	-0.53	5	0.714285714	AABR07056183.1	
DMR7:21176901	7	21176901	400	1	9.21E-06	-0.38	3	0.75		
DMR7:23583401	7	23583401	800	1	7.07E-06	1.48	15	1.875	Syn3;Timp3	Development;Proteolysis
DMR7:26035401	7	26035401	200	1	1.90E-06	-1.09	3	1.5		
DMR7:29010401	7	29010401	400	1	2.51E-06	-0.75	3	0.75	Gnptab	Transcription
DMR7:33760701	7	33760701	1100	1	6.99E-06	-0.81	26	2.363636364	AABR07056633.1	
DMR7:34870501	7	34870501	1400	1	9.77E-06	-1	16	1.142857143	AABR07073295.1;Mir331	
DMR7:47310201	7	47310201	300	1	5.83E-06	-0.85	3	1	Tmtc2	Unknown
DMR7:47403201	7	47403201	100	1	8.58E-06	-2.21	2	2	Tmtc2	Unknown
DMR7:59550801	7	59550801	800	1	3.92E-06	-1.31	7	0.875	Cnot2	Transcription
DMR7:70837701	7	70837701	2800	1	1.16E-06	-0.51	59	2.107142857	Shmt2;Nxph4;Lrp1	Metabolism;Signaling;Rec
DMR7:71675201	7	71675201	500	1	9.82E-07	-1.11	8	1.6	Sdc2	Extracellular Matrix
DMR7:73124301	7	73124301	900	1	2.26E-07	-1.32	4	0.444444444	Matn2;AABR07057475.1	Cytoskeleton
DMR7:74646401	7	74646401	700	1	1.98E-06	-0.82	13	1.857142857		
DMR7:87841901	7	87841901	400	1	1.00E-06	-1.33	7	1.75	AABR07057765.1	
DMR7:95378401	7	95378401	100	1	4.50E-06	1.84	0	0	Mtbp	
DMR7:107150801	7	107150801	400	1	5.16E-06	-1.41	2	0.5	Lrrc6	Development
DMR7:108127401	7	108127401	400	1	4.39E-06	-1.13	6	1.5		
DMR7:114772501	7	114772501	200	1	8.96E-06	1.51	3	1.5	Dennd3	Signaling
DMR7:132198001	7	132198001	600	1	5.99E-07	-1.31	6	1		

DMR7:133776901	7	133776901	1600	1	2.34E-06	-1.71	40	2.5		
DMR7:134680001	7	134680001	500	1	3.14E-06	-0.76	9	1.8	Pphln1	
DMR8:10654701	8	10654701	600	1	9.03E-06	2.26	2	0.333333333		
DMR8:11516101	8	11516101	200	1	7.19E-06	-1.29	1	0.5		
DMR8:16602601	8	16602601	1300	1	2.76E-06	-1.58	6	0.461538462		
DMR8:18578701	8	18578701	500	1	2.01E-06	-0.84	0	0		
DMR8:18685801	8	18685801	300	1	6.30E-06	-0.55	1	0.333333333		
DMR8:40496001	8	40496001	400	1	3.30E-06	-1.43	5	1.25	Olr1204;AC114252.1	
DMR8:42050701	8	42050701	500	1	9.98E-06	-1.52	5	1	Olr1229	
DMR8:47080601	8	47080601	300	1	5.18E-07	1.23	2	0.666666667	Grik4	Signaling
DMR8:50468901	8	50468901	3800	1	7.86E-06	-0.97	80	2.105263158	Sik3	Receptor
DMR8:51294001	8	51294001	300	1	1.84E-06	2.34	4	1.333333333		
DMR8:52749901	8	52749901	300	1	3.84E-06	-1.6	2	0.666666667	Nxpe1	
DMR8:58164301	8	58164301	1900	1	7.98E-06	-1	24	1.263157895	Npat;Acat1	Transcription;Metabolism
DMR8:65190501	8	65190501	4400	1	8.46E-06	-0.47	41	0.931818182	AABR07070312.1	
DMR8:65821401	8	65821401	200	1	4.89E-06	5.03	7	3.5		
DMR8:77171001	8	77171001	200	1	9.13E-07	-1.73	2	1	Adam10	Proteolysis
DMR8:99305701	8	99305701	100	1	1.62E-06	-1.22	0	0		
DMR8:119708301	8	119708301	1900	1	5.83E-06	-1.32	32	1.684210526	Dclk3	Cytoskeleton
DMR8:121151501	8	121151501	200	1	4.89E-06	-1.66	1	0.5		
DMR9:10111501	9	10111501	1100	1	2.81E-06	-1.29	6	0.545454545	Mllt1	Development
DMR9:18081301	9	18081301	400	1	9.32E-06	-1.06	11	2.75	AABR07066828.1	
DMR9:20268501	9	20268501	1000	1	3.90E-08	-1.51	18	1.8	LOC100911625;Atn1	Metabolism;Unknown
DMR9:22665101	9	22665101	200	1	5.08E-06	-0.97	3	1.5		
DMR9:25600501	9	25600501	2000	1	4.33E-06	-0.85	19	0.95		
DMR9:25839301	9	25839301	300	1	4.31E-06	-0.74	2	0.666666667	LOC108351902	
DMR9:62076001	9	62076001	2700	1	9.35E-06	-0.94	25	0.925925926		
DMR9:62351801	9	62351801	200	1	1.49E-09	-2.3	4	2	Plcl1	Metabolism
DMR9:63032001	9	63032001	300	2	2.75E-06	-1.97	0	0		
DMR9:70066301	9	70066301	300	1	1.10E-06	-1.51	3	1	Zdbf2	Transcription
DMR9:71210101	9	71210101	800	2	6.17E-07	-2.07	9	1.125		
DMR9:82924301	9	82924301	400	1	7.19E-06	1.27	2	0.5		
DMR9:90869001	9	90869001	300	1	1.84E-06	-0.84	4	1.333333333	LOC100911572	
DMR9:92956401	9	92956401	300	1	3.92E-06	-1.31	5	1.666666667	Gpr55	Receptor
DMR9:94169601	9	94169601	300	1	5.09E-06	-0.8	3	1	Dis3l2;Alppl2	Transcription;Signaling
DMR9:100106001	9	100106001	1200	1	8.91E-06	-0.89	7	0.583333333	RF00026;Capn10	Protease
DMR9:114589601	9	114589601	100	1	2.25E-07	-2.29	3	3	Mtcl1	
DMR10:1115201	10	1115201	300	1	4.57E-06	-1.73	0	0		
DMR10:4015701	10	4015701	700	1	4.52E-06	-0.9	7	1	Snx29	Cytoskeleton
DMR10:6477401	10	6477401	2300	1	1.76E-06	2.01	53	2.304347826		
DMR10:9967401	10	9967401	2000	1	1.95E-06	-1.35	18	0.9		
DMR10:19970501	10	19970501	3600	1	8.20E-06	2.09	52	1.444444444		
DMR10:22873601	10	22873601	1000	1	8.65E-06	-1.59	7	0.7		
DMR10:26640501	10	26640501	1200	1	4.53E-06	-0.68	10	0.833333333		
DMR10:27086801	10	27086801	300	1	5.73E-06	-0.97	4	1.333333333	Gabrg2	Receptor
DMR10:30221801	10	30221801	700	1	9.23E-06	-1.33	4	0.571428571		
DMR10:37911801	10	37911801	1600	1	9.77E-06	2.11	24	1.5		
DMR10:39562801	10	39562801	1200	1	9.88E-06	1.87	15	1.25		
DMR10:53705301	10	53705301	300	1	4.33E-06	-0.86	3	1	Myh1	Cytoskeleton
DMR10:57175901	10	57175901	900	1	1.53E-06	-1.1	11	1.222222222	Pld2;Mink1	Metabolism;Signaling
DMR10:59836601	10	59836601	400	1	6.16E-06	-0.6	3	0.75	Trpv3;Aspa	Transport;Metabolism
DMR10:64492701	10	64492701	1200	1	5.49E-06	-1.02	19	1.583333333	Nxn	Signaling
DMR10:70989201	10	70989201	300	1	3.92E-06	-0.54	5	1.666666667	AABR07030143.1	
DMR10:71214101	10	71214101	1000	1	4.52E-06	-0.92	11	1.1	Hnf1b	Transcription
DMR10:71802001	10	71802001	400	1	4.23E-06	-1.06	3	0.75	Aatf	Transcription
DMR10:77295101	10	77295101	300	1	2.19E-06	-0.93	4	1.333333333		
DMR10:79689201	10	79689201	300	1	8.61E-07	-1.42	0	0		
DMR10:102417701	10	102417701	400	1	4.54E-06	-0.58	4	1	Sdk2	Development
DMR10:104380801	10	104380801	1100	1	6.66E-06	-2.23	19	1.727272727	Llgl2	Development
DMR10:110967601	10	110967601	300	1	5.35E-07	-2.29	3	1		
DMR11:10012701	11	10012701	1300	1	2.33E-07	-1.96	18	1.384615385	Robo1	Development
DMR11:14472901	11	14472901	300	1	6.30E-06	1.8	6	2		
DMR11:20977101	11	20977101	400	1	8.18E-06	-1.09	1	0.25		

DMR11:34928601	11	34928601	1200	1	7.76E-06	-1.59	16	1.333333333	Dyrk1a	Signaling
DMR11:41278201	11	41278201	1200	1	8.03E-07	-1.85	6	0.5		
DMR11:44983901	11	44983901	200	1	6.03E-06	-0.94	4	2	Col8a1	Cytoskeleton
DMR11:58334901	11	58334901	300	1	7.16E-06	-1.34	0	0		
DMR11:60769601	11	60769601	200	1	4.19E-06	-2	0	0		
DMR11:64330301	11	64330301	900	1	5.68E-06	-0.88	17	1.888888889	Igsf11	Extracellular Matrix
DMR11:68415801	11	68415801	800	1	1.99E-06	-1.02	10	1.25		
DMR11:74535601	11	74535601	800	1	7.60E-06	-1.18	8	1		
DMR11:75882401	11	75882401	1400	1	7.64E-07	-1.66	15	1.071428571		
DMR11:78752201	11	78752201	300	1	5.86E-06	-1.53	2	0.666666667	Tprg1	
DMR11:79429601	11	79429601	200	1	5.26E-06	-1.08	5	2.5	Lpp	Cytoskeleton
DMR11:80223901	11	80223901	400	1	1.75E-06	-1.09	3	0.75		
DMR11:83549501	11	83549501	300	1	7.24E-06	-0.76	4	1.333333333	Ephb3	Receptor
DMR11:86826201	11	86826201	300	1	1.41E-06	-1.47	1	0.333333333	Tango2	
DMR11:89163301	11	89163301	200	1	6.85E-06	-1.59	2	1	Spidr	
DMR12:21074501	12	21074501	1000	1	9.77E-06	-0.93	4	0.4	Vom2r64	
DMR12:23783301	12	23783301	1200	1	7.58E-06	1.53	16	1.333333333	Ywhag	Protein Binding
DMR12:23945501	12	23945501	200	1	3.16E-06	-1.66	3	1.5	Mdh2;Styxl1	Metabolism;Signaling
DMR12:29587501	12	29587501	1900	1	8.12E-07	-1.45	22	1.157894737	Caln1	Signaling
DMR12:31601401	12	31601401	800	1	5.18E-06	-1.83	16	2	Rimbp2;Piwil1	Unknown
DMR12:31913301	12	31913301	1200	1	6.37E-06	-0.63	11	0.916666667		
DMR12:36906801	12	36906801	2800	1	2.23E-08	-1.22	53	1.892857143	Ncor2	Transcription
DMR12:38798601	12	38798601	800	1	6.87E-06	-1.51	13	1.625	Psmd9	Proteolysis
DMR12:40161701	12	40161701	200	1	5.38E-06	1.16	4	2	Cux2	Development
DMR12:51789301	12	51789301	1300	1	2.27E-06	-1.09	9	0.692307692	AC095390.2	
DMR13:11598201	13	11598201	1800	1	2.58E-06	-0.59	10	0.555555556		
DMR13:35613901	13	35613901	400	1	7.28E-06	-1.61	4	1	Epb41l5	
DMR13:38547001	13	38547001	300	1	9.83E-06	-2.1	1	0.333333333		
DMR13:40167201	13	40167201	700	1	5.05E-06	-1.35	3	0.428571429		
DMR13:40639601	13	40639601	300	1	6.72E-06	-0.78	2	0.666666667		
DMR13:42052601	13	42052601	200	1	7.44E-08	-2.04	4	2		
DMR13:43671101	13	43671101	900	1	1.04E-06	-0.79	5	0.555555556		
DMR13:46037301	13	46037301	300	1	9.98E-06	-1.38	4	1.333333333		
DMR13:50032501	13	50032501	1300	1	8.25E-07	1.89	36	2.769230769		
DMR13:51010101	13	51010101	600	1	7.12E-06	-0.5	2	0.333333333		
DMR13:58214201	13	58214201	1100	1	3.43E-07	-1.72	3	0.272727273		
DMR13:67435601	13	67435601	300	1	4.04E-06	-1.28	1	0.333333333	AABR07021355.1	
DMR13:88252701	13	88252701	1200	1	5.63E-07	-0.71	6	0.5		
DMR13:98027501	13	98027501	1800	2	2.54E-07	-1.16	20	1.111111111	Kif28p;Ahctf1	Transcription
DMR13:98097901	13	98097901	600	1	3.62E-06	-1.19	7	1.166666667		
DMR13:107477201	13	107477201	1300	1	5.46E-06	-1.39	15	1.153846154	Kctd3	Metabolism
DMR13:108667601	13	108667601	400	1	2.91E-06	-1.05	8	2	Smyd2	Transcription
DMR13:110704401	13	110704401	600	1	3.79E-06	-0.69	6	1		
DMR13:111760501	13	111760501	300	1	1.82E-06	-2.5	4	1.333333333	Syt14	Transport
DMR14:1235001	14	1235001	100	1	4.50E-06	1.98	0	0		
DMR14:8688301	14	8688301	200	1	8.92E-06	-0.88	0	0		
DMR14:9826701	14	9826701	300	1	1.12E-06	-0.94	2	0.666666667		
DMR14:20534801	14	20534801	700	1	9.61E-06	-0.86	11	1.571428571	Slc4a4	Metabolism
DMR14:39021301	14	39021301	2000	1	7.59E-06	-1.44	13	0.65	Gabrb1	Receptor
DMR14:53148401	14	53148401	300	1	5.29E-06	-0.8	2	0.666666667		
DMR14:59791201	14	59791201	400	1	7.95E-07	-0.77	6	1.5		
DMR14:60822001	14	60822001	200	1	2.71E-07	-1.43	2	1		
DMR14:77476901	14	77476901	700	1	3.83E-06	-1.73	5	0.714285714	Stx18	Transport
DMR14:83207201	14	83207201	200	1	1.55E-06	-1.38	2	1	Depdc5;AC105515.2	Unknown
DMR14:95040101	14	95040101	1700	1	1.21E-06	-0.99	38	2.235294118		
DMR14:95144001	14	95144001	2400	1	4.64E-08	-0.85	44	1.833333333		
DMR14:95897201	14	95897201	300	1	1.81E-06	-1.16	4	1.333333333		
DMR14:99448701	14	99448701	200	1	6.40E-06	-0.99	1	0.5		
DMR14:101694601	14	101694601	1600	1	6.72E-06	-0.75	42	2.625		
DMR14:103207401	14	103207401	1300	1	9.35E-06	-1	12	0.923076923	Meis1	Transcription
DMR14:104593201	14	104593201	2300	1	4.57E-06	-0.86	40	1.739130435	Slc1a4	Metabolism
DMR15:2095101	15	2095101	1000	1	2.34E-06	-0.86	16	1.6		
DMR15:3065301	15	3065301	2600	1	2.28E-09	2.41	39	1.5	Adk	Signaling

DMR15:14444101	15	14444101	300	1	1.41E-07	-1.43	1	0.333333333		
DMR15:15292601	15	15292601	900	1	6.29E-06	2.45	10	1.111111111	Cadps	Metabolism
DMR15:23720101	15	23720101	400	1	1.26E-06	-0.92	2	0.5	Samd4a	Signaling
DMR15:33888301	15	33888301	400	1	5.75E-06	-1.16	2	0.5	Dhrs2	Metabolism
DMR15:46426801	15	46426801	200	1	2.32E-08	2.82	2	1	Gata4	Transcription
DMR15:56905101	15	56905101	300	1	5.71E-06	1.66	5	1.666666667	Lrch1	Unknown
DMR15:60066501	15	60066501	300	1	7.00E-06	-0.58	3	1	Dnajc15	Transcription
DMR15:67929001	15	67929001	300	1	1.02E-06	-1.36	3	1		
DMR15:86533701	15	86533701	2300	1	4.53E-06	-2.26	24	1.043478261		
DMR15:86538001	15	86538001	400	1	4.93E-06	-0.9	3	0.75		
DMR15:87925201	15	87925201	200	1	4.39E-06	-1.44	1	0.5	Mycbp2	Metabolism
DMR15:90381901	15	90381901	600	1	8.33E-06	-0.98	8	1.333333333	Mycbp2	Metabolism
DMR15:90831201	15	90831201	300	1	3.29E-06	-1.53	4	1.333333333	Mycbp2;AABR07019140.1	Metabolism
DMR15:102374101	15	102374101	600	1	9.20E-06	1.23	12	2	Gpc6	Cytoskeleton
DMR16:10521901	16	10521901	2700	1	7.39E-06	-0.9	48	1.777777778	Gprin2	
DMR16:11274601	16	11274601	900	1	4.34E-06	-1.1	8	0.888888889		
DMR16:17910601	16	17910601	200	1	2.04E-06	-1.61	1	0.5		
DMR16:22021701	16	22021701	1000	1	3.15E-06	1.51	23	2.3	Potem	
DMR16:31201101	16	31201101	400	1	5.92E-06	-0.94	2	0.5		
DMR16:33952401	16	33952401	300	1	2.86E-06	-1.36	2	0.666666667	AABR07025328.1	
DMR16:38461201	16	38461201	1200	1	3.27E-08	-1.27	9	0.75		
DMR16:47162501	16	47162501	500	1	5.26E-06	-1.07	15	3	LOC100911140	
DMR16:57152001	16	57152001	200	1	7.28E-06	-1.5	1	0.5		
DMR16:59903901	16	59903901	1000	1	2.26E-06	-0.73	11	1.1		
DMR16:72541501	16	72541501	1500	1	7.79E-06	-1.19	18	1.2		
DMR16:73283101	16	73283101	1800	1	7.65E-06	-0.96	20	1.111111111		
DMR16:77958401	16	77958401	500	1	1.81E-06	-1.29	2	0.4		
DMR16:78360801	16	78360801	1300	1	4.75E-06	-1.3	15	1.153846154	AABR07026483.1	
DMR16:85731501	16	85731501	300	1	1.62E-06	-1.38	2	0.666666667		
DMR16:86948101	16	86948101	1000	1	8.57E-06	-1.43	7	0.7		
DMR16:86969701	16	86969701	400	1	4.04E-06	-0.49	4	1		
DMR17:1591101	17	1591101	2300	1	2.47E-06	-1.31	27	1.173913043	Hsd17b3	Metabolism
DMR17:9192101	17	9192101	200	1	8.37E-06	-1.93	2	1		
DMR17:28013101	17	28013101	200	1	1.09E-06	-2.35	3	1.5	AABR07027394.2;RF00003	
DMR17:33592601	17	33592601	1900	1	2.04E-06	-0.7	22	1.157894737	Gmds	Signaling
DMR17:43537101	17	43537101	100	1	4.65E-06	-2.59	1	1	Slc17a3	Metabolism
DMR17:44954601	17	44954601	1000	1	4.52E-07	-1.49	9	0.9	Olr1657	Receptor
DMR17:47885401	17	47885401	1100	1	5.10E-06	1.26	14	1.272727273	AABR07027872.1	
DMR17:54179401	17	54179401	300	1	1.60E-06	-1.2	0	0	LOC108348568;Kif5b	Cytoskeleton
DMR17:58239701	17	58239701	200	1	5.63E-06	2.31	3	1.5	Adarb2	Transcription
DMR17:64194601	17	64194601	1200	1	6.66E-06	-1.64	13	1.083333333		
DMR17:65242301	17	65242301	1000	1	3.10E-06	-1.17	8	0.8		
DMR17:65288301	17	65288301	600	1	5.39E-06	1.81	5	0.833333333		
DMR17:72026901	17	72026901	1300	1	5.43E-06	1.25	26	2	AABR07028488.1	
DMR17:72274601	17	72274601	200	1	3.56E-07	-1.59	6	3	Atp5f1c	
DMR17:73557601	17	73557601	1700	1	6.59E-06	-1.83	20	1.176470588		
DMR17:78516701	17	78516701	400	1	2.70E-06	-1.243	14	3.5	Fam107b	
DMR17:81987301	17	81987301	1300	1	2.17E-06	-0.95	9	0.692307692	Cacnb2	Transport
DMR17:86729301	17	86729301	400	1	6.08E-06	-0.88	2	0.5		
DMR18:1991201	18	1991201	400	1	3.29E-06	-1.01	3	0.75	Mib1	Metabolism
DMR18:2445001	18	2445001	300	1	9.73E-06	-0.84	3	1	Gata6	Transcription
DMR18:14734001	18	14734001	300	1	7.08E-06	-1.65	4	1.333333333	RGD1563861	Transcription
DMR18:16304701	18	16304701	200	1	9.96E-07	-2.87	2	1		
DMR18:22678601	18	22678601	100	1	5.98E-06	-1.83	1	1		
DMR18:26986201	18	26986201	200	1	1.97E-06	-1.5	3	1.5	RF00066;AABR07031689.1	
DMR18:46684701	18	46684701	300	1	9.39E-06	-2.03	4	1.333333333		
DMR18:48835001	18	48835001	300	1	3.09E-06	-1.92	0	0	AABR07032135.1	
DMR18:55805601	18	55805601	100	1	2.82E-06	-1.29	1	1	Dctn4	Cytoskeleton
DMR18:62341901	18	62341901	200	1	4.87E-06	-1.73	1	0.5		
DMR18:75812101	18	75812101	2900	1	3.27E-06	-0.86	24	0.827586207		
DMR18:84870501	18	84870501	700	1	1.50E-06	-2.26	2	0.285714286		
DMR19:10838301	19	10838301	300	1	4.56E-06	-1.27	6	2	Fam192a;RF00392	
DMR19:12602301	19	12602301	900	1	1.48E-06	-1.4	11	1.222222222	Large1	

DMR19:16697501	19	16697501	300	1	9.24E-07	-1.06	8	2.666666667		
DMR19:42275101	19	42275101	1000	1	7.33E-06	-1.04	16	1.6		
DMR19:56379601	19	56379601	900	1	9.94E-06	-1.45	11	1.222222222		
DMR19:58574701	19	58574701	600	1	4.13E-07	-0.74	8	1.333333333	Ntpcr;Pcnx2	
DMR20:8304801	20	8304801	200	1	6.17E-06	-0.57	8	4	Rnf8;Cmtr1	Proteolysis
DMR20:11885701	20	11885701	1100	1	6.83E-07	1.49	24	2.181818182	Fam207a	
DMR20:14464301	20	14464301	1600	1	1.76E-06	-0.67	26	1.625	Bcr	DNA repair
DMR20:14724401	20	14724401	3400	1	7.36E-07	-1.26	77	2.264705882		
DMR20:15134301	20	15134301	2800	2	6.35E-06	-0.87	28	1	Pcdh15	Cytoskeleton
DMR20:15626201	20	15626201	1100	1	9.90E-06	-1.38	11	1		
DMR20:27397601	20	27397601	200	1	8.01E-06	-1.15	2	1	Tet1	
DMR20:31989801	20	31989801	300	1	4.59E-06	-1.69	1	0.333333333		
DMR20:44493401	20	44493401	1900	1	7.73E-06	-1.06	21	1.105263158	Fyn	Transcription
DMR20:44521601	20	44521601	2700	1	6.78E-06	1.1	67	2.481481481	Fyn	Transcription
DMRX:14333201	X	14333201	500	1	6.60E-06	-0.89	5	1	Rpgr	Metabolism
DMRX:17463801	X	17463801	700	1	9.37E-06	-1.33	4	0.571428571		
DMRX:18239401	X	18239401	300	1	8.05E-06	-1.33	2	0.666666667		
DMRX:59801301	X	59801301	200	1	4.05E-06	-1.67	2	1		
DMRX:79387501	X	79387501	200	1	4.32E-08	-2.02	2	1		
DMRX:83687801	X	83687801	200	1	4.19E-06	-2	1	0.5	Apool	Metabolism
DMRX:85113501	X	85113501	100	1	1.19E-08	-2.55	0	0	RF00322	
DMRX:117534201	X	117534201	200	1	8.20E-06	1.76	1	0.5		
DMRX:121320301	X	121320301	300	1	7.21E-06	-0.62	3	1		
DMRX:139292501	X	139292501	300	1	1.83E-06	-1.43	1	0.333333333		
DMRX:140094901	X	140094901	300	1	5.04E-06	-0.63	4	1.333333333		
DMRX:148066201	X	148066201	200	1	3.16E-06	-1.57	2	1		
DMRX:149274301	X	149274301	3800	1	6.93E-06	-0.87	13	0.342105263	AABR07042135.1	
DMRX:149572401	X	149572401	300	1	4.75E-06	-1.3	2	0.666666667		
DMRX:150981001	X	150981001	300	1	1.74E-06	-1.61	4	1.333333333		
