# Supplementary material for: Vinclozolin induced epigenetic transgenerational inheritance of pathologies and sperm epimutation biomarkers for specific diseases
Source: PLoS One. 2018 Aug 29;13(8):e0202662. doi: 10.1371/journal.pone.0202662 (PMC6114855; doi:10.1371/journal.pone.0202662)
Supplement: S6 Table — DMR name, chromosome, start, length, number of signature windows, minimum p-value, CpG number, CpG density, maximum log fold change, annotation, gene and functional category presented. (PDF) [file pone.0202662.s007.pdf]

Supplemental Table S6
Kidney Disease DMR Signature List

DMR Name	Chr	Start	Length	# Sig Win	minP	Log Fold Change	CpG #	CpG Density	Gene Annotation	Gene Category
DMR1:6043801	1	6043801	400	1	5.17E-06	1.14	4	1	RF00322	
DMR1:20416801	1	20416801	400	1	9.33E-06	1.17	3	0.75	Tmem200a;RGD1559962	Unknown;Epigenetics
DMR1:25572201	1	25572201	500	1	3.99E-06	-2.1	0	0	Trdn	Cytoskeleton
DMR1:29501501	1	29501501	200	1	6.65E-06	1.268	0	0		
DMR1:32225401	1	32225401	1000	1	6.52E-06	1.09	11	1.1	Slc6a19;Slc6a18	Transport
DMR1:60618601	1	60618601	400	1	3.35E-07	1.33	9	2.25		
DMR1:61793601	1	61793601	1400	1	2.77E-06	1.47	33	2.357142857	AABR07001905.1	
DMR1:63969901	1	63969901	200	1	4.77E-06	1.55	1	0.5	Lilrb3a;AABR07002001.1	
DMR1:75108101	1	75108101	2100	1	6.56E-06	2.22	28	1.333333333		
DMR1:76225401	1	76225401	800	1	7.18E-06	1.88	32	4		
DMR1:76490201	1	76490201	500	1	3.19E-06	1.47	6	1.2	Sult2a2	Metabolism
DMR1:76504201	1	76504201	200	1	7.92E-06	1.74	0	0	Sult2a2	Metabolism
DMR1:76602401	1	76602401	200	1	5.77E-06	1.48	0	0	Sult2a2	Metabolism
DMR1:77295201	1	77295201	900	2	1.92E-06	0.57	2	0.222222222		
DMR1:109469501	1	109469501	300	1	1.12E-07	1.14	0	0	AABR07003418.1	
DMR1:115686101	1	115686101	200	1	6.56E-06	1.94	1	0.5		
DMR1:118206301	1	118206301	1000	1	1.65E-07	2.13	7	0.7		
DMR1:127013301	1	127013301	200	1	3.10E-06	1.26	2	1	Chsy1	Metabolism
DMR1:132164801	1	132164801	100	1	6.24E-06	-0.88	7	7		
DMR1:140705801	1	140705801	300	1	5.72E-06	0.96	1	0.333333333		
DMR1:145128801	1	145128801	200	1	2.42E-06	2.25	1	0.5		
DMR1:145506101	1	145506101	700	1	3.44E-06	2.76	4	0.571428571		
DMR1:152849601	1	152849601	300	1	4.79E-07	1.56	3	1		
DMR1:180800801	1	180800801	10000	1	8.52E-06	-0.55	261	2.61		
DMR1:181215001	1	181215001	800	1	5.47E-06	-0.39	22	2.75		
DMR1:182008401	1	182008401	5000	1	6.41E-06	-0.48	120	2.4		
DMR1:196608601	1	196608601	300	1	9.84E-06	2.27	2	0.666666667		
DMR1:199546201	1	199546201	1700	1	1.84E-06	-2.22	17	1	Itgad;Itgax	Extracellular Matrix
DMR1:236275001	1	236275001	300	1	2.27E-06	1.51	0	0	Pcsk5	Protease
DMR1:245850801	1	245850801	700	1	6.74E-06	-1.23	13	1.857142857	Rfx3	Transcription
DMR1:273437101	1	273437101	300	1	3.06E-06	2.06	1	0.333333333		
DMR2:3628901	2	3628901	1000	1	3.64E-06	1.71	5	0.5	Mctp1;RF00560	Unknown
DMR2:28888301	2	28888301	200	1	2.13E-06	2.48	0	0	AABR07007802.1	
DMR2:33675901	2	33675901	100	1	1.29E-06	2.76	0	0		
DMR2:36137401	2	36137401	1400	1	8.88E-06	0.91	11	0.785714286		
DMR2:39517401	2	39517401	800	1	2.98E-08	2.65	13	1.625	AABR07008025.1	
DMR2:46539101	2	46539101	400	1	7.70E-06	-1.51	5	1.25	Fst	Growth factor
DMR2:55660801	2	55660801	100	1	1.35E-06	-2.09	1	1		
DMR2:56390701	2	56390701	1000	1	3.53E-08	1.9	5	0.5		
DMR2:56732901	2	56732901	300	1	1.66E-10	3.33	0	0		
DMR2:60202701	2	60202701	200	1	1.90E-06	-1.44	2	1	Prlr	Receptor
DMR2:61120701	2	61120701	400	2	4.75E-06	5.61	3	0.75	Adamts12	Protease
DMR2:94480801	2	94480801	1400	1	6.12E-06	1.5	21	1.5	Zfp704	Transcription
DMR2:103722701	2	103722701	1300	1	5.53E-06	1.69	9	0.692307692		
DMR2:105433301	2	105433301	200	1	4.23E-06	-1.45	6	3		
DMR2:149305601	2	149305601	100	1	8.41E-06	1.61	1	1	Med12l	Transcription
DMR2:156575501	2	156575501	1100	1	6.07E-06	-0.543	27	2.454545455		
DMR2:176994901	2	176994901	300	1	1.36E-06	0.81	3	1		
DMR2:189953301	2	189953301	300	1	8.29E-06	-1.97	4	1.333333333	S100a3;RF00003	Receptor
DMR2:197677901	2	197677901	100	1	3.34E-06	-1.68	4	4	Ctss;Hormad1	Proteolysis;Development
DMR2:207388501	2	207388501	200	1	3.74E-07	-1.41	2	1	St7l	Unknown
DMR2:207940601	2	207940601	400	1	3.44E-06	2.46	1	0.25	Kcnd3	Transport
DMR2:229236801	2	229236801	500	1	5.42E-06	1.42	2	0.4	Ndst4	Metabolism
DMR2:243143501	2	243143501	200	1	9.47E-06	-1.62	3	1.5	Dnajb14	Protein Binding
DMR3:85077601	3	85077601	300	1	8.42E-07	1.24	1	0.333333333		
DMR3:112411201	3	112411201	1100	1	6.43E-06	-1.82	20	1.818181818		
DMR3:120330001	3	120330001	300	1	5.86E-07	-1.86	2	0.666666667	AABR07053687.1	
DMR3:134020001	3	134020001	1700	1	2.87E-06	-0.41	38	2.235294118		
DMR3:149639501	3	149639501	200	1	5.63E-06	2.09	1	0.5	Bpifa1;Bpifa5	
DMR3:154662501	3	154662501	300	1	7.57E-06	1.04	0	0		

DMR3:163659401	3	163659401	400	1	2.85E-06	0.94	9	2.25	Arfgef2;AC130053.1;Cse1l	Signaling;Transport
DMR3:166861601	3	166861601	300	1	8.39E-06	-1.28	7	2.333333333		
DMR3:172079601	3	172079601	700	1	7.90E-06	0.6	1	0.142857143		
DMR4:4028801	4	4028801	200	1	1.88E-06	1.59	0	0	Dpp6	Proteolysis
DMR4:68148201	4	68148201	200	1	5.48E-06	1.58	0	0		
DMR4:79830501	4	79830501	900	1	4.97E-06	1.46	4	0.444444444		
DMR4:113244601	4	113244601	800	1	8.06E-06	1.24	18	2.25	Tacr1	Receptor
DMR4:153168301	4	153168301	2500	1	4.22E-06	1.55	34	1.36		
DMR4:165432701	4	165432701	100	1	4.92E-06	2.36	1	1	Klra2	Immune response
DMR4:171653301	4	171653301	1300	1	1.12E-06	0.67	11	0.846153846		
DMR5:32751801	5	32751801	900	1	7.55E-06	0.81	7	0.777777778	Cnbd1	
DMR5:49760901	5	49760901	300	1	2.39E-06	2.69	6	2		
DMR5:74341701	5	74341701	1400	2	1.06E-07	1.38	25	1.785714286	Ptpn3	Signaling
DMR5:78777301	5	78777301	900	1	6.00E-07	1.31	6	0.666666667		
DMR5:107158001	5	107158001	300	1	2.97E-06	1.02	1	0.333333333		
DMR5:119829901	5	119829901	400	1	4.83E-06	2.76	4	1	Cachd1	Unknown
DMR5:142345801	5	142345801	200	1	5.63E-06	2.37	2	1	LOC100909856	
DMR5:153530401	5	153530401	200	1	4.51E-06	1.8	4	2	Runx3	Transcription
DMR5:156076201	5	156076201	300	1	3.67E-06	0.85	0	0	Rap1gap;Alpl	Signaling;Metabolism
DMR5:161615601	5	161615601	700	1	7.76E-07	-2.3	21	3		
DMR5:163078801	5	163078801	2400	1	6.64E-06	1.37	11	0.458333333	Vps13d	
DMR5:170757601	5	170757601	300	1	2.14E-06	1.74	2	0.666666667		
DMR5:171389001	5	171389001	3600	1	6.53E-08	1.12	63	1.75	Tp73	Transcription
DMR6:34727101	6	34727101	3000	1	7.30E-06	2.38	28	0.933333333		
DMR6:70222401	6	70222401	600	1	8.62E-06	1.19	1	0.166666667	RF00100;AABR07064230.1	
DMR6:71791801	6	71791801	400	1	3.09E-06	1.22	4	1		
DMR6:80428901	6	80428901	200	1	9.74E-06	-1.86	0	0		
DMR7:30720401	7	30720401	300	1	3.11E-06	-1.77	7	2.333333333	Anks1b	Transcription
DMR7:65319601	7	65319601	200	1	2.57E-06	1.28	8	4		
DMR7:72356601	7	72356601	300	1	9.46E-06	1.37	0	0		
DMR7:72498401	7	72498401	300	1	6.71E-06	1.21	2	0.666666667		
DMR7:72750001	7	72750001	1500	1	8.47E-06	2.6	15	1		
DMR7:86101001	7	86101001	1300	1	4.37E-06	1.15	8	0.615384615		
DMR7:95075301	7	95075301	1200	1	1.38E-06	1.56	20	1.666666667	Col14a1	Cytoskeleton
DMR7:112287601	7	112287601	300	1	9.88E-06	1.15	0	0		
DMR7:116425601	7	116425601	900	1	6.74E-06	0.94	8	0.888888889	AABR07058441.1;Ly6i	Immune response
DMR7:117273801	7	117273801	200	1	6.46E-06	1.3	5	2.5	Plec	Cytoskeleton
DMR7:120115101	7	120115101	200	1	7.78E-06	2.51	1	0.5	Gga1;AC096473.1	Transport
DMR7:122952501	7	122952501	2400	1	2.26E-07	1.89	32	1.333333333	Rangap1	Signaling
DMR7:126916401	7	126916401	300	2	8.76E-09	2.02	5	1.666666667	Celsr1	Receptor
DMR7:127520101	7	127520101	300	1	1.85E-06	1.32	7	2.333333333		
DMR7:134233301	7	134233301	400	2	9.83E-07	0.91	2	0.5		
DMR7:139592501	7	139592501	500	1	7.87E-06	0.97	4	0.8		
DMR8:5810301	8	5810301	1400	1	8.16E-06	1.58	13	0.928571429	Mmp27	Protease
DMR8:19176801	8	19176801	100	1	5.40E-06	-1.79	1	1	Olr1138;LOC100911988	
DMR8:28392701	8	28392701	500	1	7.84E-06	1.37	10	2	Igsf9b	
DMR8:58687901	8	58687901	1300	1	2.96E-06	1.18	11	0.846153846		
DMR8:93645801	8	93645801	1000	2	2.14E-07	2.08	5	0.5		
DMR8:119377801	8	119377801	100	1	6.65E-06	1.25	2	2	AABR07073453.1;Lrrfip2	Transcription
DMR9:36215801	9	36215801	2300	1	1.40E-06	1.09	12	0.52173913		
DMR9:64136401	9	64136401	200	1	8.73E-07	-1.95	3	1.5		
DMR9:82715101	9	82715101	300	1	3.56E-06	1.07	3	1	Stk11ip	Signaling
DMR9:95728701	9	95728701	1700	1	9.97E-06	1.16	9	0.529411765		
DMR9:98552001	9	98552001	300	2	8.60E-08	1.19	10	3.333333333	Per2;Hes6	Development;Transcription
DMR9:100420701	9	100420701	400	1	8.85E-06	1.6	6	1.5	AABR07068351.2;Sned1	Signaling
DMR9:120163001	9	120163001	800	1	7.19E-06	1.31	6	0.75		
DMR10:2287701	10	2287701	200	2	1.92E-06	1.22	1	0.5		
DMR10:3389401	10	3389401	300	1	7.01E-07	-1.01	2	0.666666667	RF00001	
DMR10:12565901	10	12565901	300	1	6.54E-06	-1.25	2	0.666666667	AC129054.2;Olr1372	Receptor
DMR10:29171001	10	29171001	1200	1	3.54E-06	0.66	15	1.25	Ccnjl	Signaling
DMR10:37507901	10	37507901	500	1	1.42E-06	2.12	6	1.2		
DMR10:41884201	10	41884201	900	1	5.35E-06	3.05	11	1.222222222	AABR07029634.1	
DMR10:47748901	10	47748901	400	1	6.58E-06	-0.46	4	1		
DMR10:49560901	10	49560901	1200	1	3.72E-06	-1.94	12	1	Pmp22	Development

DMR10:52387101	10	52387101	300	1	3.90E-07	2.03	2	0.666666667	Dnah9	Cytoskeleton
DMR10:55751901	10	55751901	2100	1	2.69E-06	2.59	31	1.476190476	Alox12b	
DMR10:57232901	10	57232901	400	1	8.69E-06	1.3	11	2.75	Mink1;Chrne;LOC687707	Signaling;Receptor
DMR10:86665101	10	86665101	500	1	1.47E-06	1.84	6	1.2	Thra	Receptor
DMR10:101361601	10	101361601	1300	1	1.37E-06	1.71	22	1.692307692		
DMR10:112279401	10	112279401	1400	1	8.69E-06	-0.72	27	1.928571429		
DMR11:680201	11	680201	1300	1	4.79E-06	1	4	0.307692308	Epha3	Receptor
DMR11:29318801	11	29318801	300	1	5.14E-06	-1.33	5	1.666666667		
DMR11:46634401	11	46634401	200	1	9.95E-06	1.89	0	0	Impg2	Receptor
DMR11:77980901	11	77980901	100	1	7.35E-06	-0.96	0	0		
DMR11:88071201	11	88071201	400	1	4.84E-06	1.06	6	1.5	Ube2l3	Metabolism
DMR12:408201	12	408201	1300	1	5.46E-06	-1.16	30	2.307692308		
DMR12:3349801	12	3349801	8000	4	3.23E-06	-0.43	205	2.5625		
DMR12:6621701	12	6621701	200	1	6.86E-06	-1.78	2	1	AABR07035194.1	
DMR12:22858801	12	22858801	1100	1	4.84E-06	1.93	16	1.454545455	Col26a1	Extracellular Matrix
DMR12:35086101	12	35086101	900	1	8.45E-06	0.9	2	0.222222222		
DMR12:42750201	12	42750201	1100	1	2.79E-06	0.53	8	0.727272727		
DMR12:44365101	12	44365101	1000	1	9.69E-09	-1.68	22	2.2	Nos1	Metabolism
DMR12:45380101	12	45380101	1700	1	5.31E-06	1.09	25	1.470588235		
DMR12:48103101	12	48103101	700	1	3.63E-06	-1.32	13	1.857142857	Foxn4	Transcription
DMR12:52445401	12	52445401	1200	1	4.94E-07	2.25	8	0.666666667	Pole;Pxmp2	Transcription
DMR13:8758401	13	8758401	200	1	2.10E-06	1.82	2	1		
DMR13:11812601	13	11812601	300	1	1.77E-06	1.37	0	0		
DMR13:22528401	13	22528401	3200	1	7.71E-06	2.24	134	4.1875	Cntnap5b	Cytoskeleton
DMR13:33485301	13	33485301	1200	1	5.85E-07	-0.43	35	2.916666667		
DMR13:33813601	13	33813601	1400	1	4.91E-07	-0.44	40	2.857142857		
DMR13:35207501	13	35207501	1300	1	3.29E-06	1.91	12	0.923076923		
DMR13:42910401	13	42910401	200	1	5.67E-06	2.14	6	3		
DMR13:51085901	13	51085901	2600	1	7.65E-06	-1.44	25	0.961538462	Adora1	Receptor
DMR13:107320901	13	107320901	500	1	7.24E-06	-1.41	4	0.8	Ush2a	Extracellular Matrix
DMR14:13687901	14	13687901	3900	1	9.25E-06	0.9	31	0.794871795		
DMR14:19854901	14	19854901	1300	1	5.35E-06	1.01	12	0.923076923		
DMR14:42938601	14	42938601	300	1	9.24E-06	0.99	5	1.666666667	Limch1	Cytoskeleton
DMR14:48647501	14	48647501	200	1	7.69E-06	0.81	2	1	Dthd1	
DMR14:51871401	14	51871401	300	1	1.34E-07	1.43	0	0	RF00026	
DMR14:53424201	14	53424201	300	1	4.55E-06	-0.51	6	2		
DMR14:67685001	14	67685001	600	1	5.81E-06	0.68	4	0.666666667		
DMR14:88037201	14	88037201	1200	1	9.24E-07	0.86	7	0.583333333		
DMR15:9508101	15	9508101	100	1	7.48E-06	-2.03	2	2		
DMR15:15245601	15	15245601	1200	1	3.76E-06	1.14	4	0.333333333		
DMR15:40093101	15	40093101	1700	1	4.44E-07	-2.34	32	1.882352941	Atp8a2	Transport
DMR15:82217301	15	82217301	1200	1	8.41E-06	-1.51	10	0.833333333	Dach1	Transcription
DMR15:82887301	15	82887301	200	1	1.46E-06	2.3	0	0		
DMR15:91000601	15	91000601	2400	1	5.57E-06	-0.84	47	1.958333333	Mycbp2	Metabolism
DMR15:106514501	15	106514501	2100	1	5.63E-06	3.68	57	2.714285714	Farp1	Signaling
DMR15:108973801	15	108973801	300	1	7.70E-06	1.26	13	4.333333333	Pcca;AC123185.4	Metabolism
DMR16:13859401	16	13859401	1100	1	5.56E-06	1.95	6	0.545454545		
DMR16:29666201	16	29666201	200	1	8.63E-06	1.69	0	0	Anxa10	Binding Protein
DMR16:51604801	16	51604801	200	1	9.62E-06	-1.46	2	1		
DMR17:11804901	17	11804901	1400	1	5.69E-07	-0.34	13	0.928571429		
DMR17:30211401	17	30211401	300	1	4.11E-07	-1.44	9	3		
DMR17:35829401	17	35829401	400	1	9.60E-06	0.85	1	0.25		
DMR17:58741501	17	58741501	300	1	2.29E-06	1.45	0	0		
DMR17:75765701	17	75765701	300	1	3.91E-06	1.38	2	0.666666667	Usp6nl	Signaling
DMR17:83786301	17	83786301	300	1	4.33E-06	1	1	0.333333333		
DMR17:84756501	17	84756501	1000	1	2.43E-07	1.9	17	1.7		
DMR18:16701701	18	16701701	800	1	2.63E-06	0.78	15	1.875	Fhod3;RF00100	Cytoskeleton
DMR18:37712401	18	37712401	700	1	3.18E-06	2.48	3	0.428571429	Stk32a;Dpysl3	Signaling;Metabolism
DMR18:41663001	18	41663001	200	1	2.85E-06	-1.84	2	1		
DMR18:71487001	18	71487001	300	1	2.02E-06	1.06	3	1	Ctif	
DMR19:5811801	19	5811801	400	1	9.08E-06	-1.99	1	0.25		
DMR19:6400801	19	6400801	300	1	1.78E-06	-0.99	2	0.666666667	Cdh8	Extracellular Matrix
DMR19:52315101	19	52315101	700	1	8.80E-06	1.24	12	1.714285714	Wfdc1	Growth factor
DMR20:45545801	20	45545801	200	1	7.93E-06	-1.68	1	0.5	Cdk19	Signaling

DMR20:47219801	20	47219801	2300	1	6.56E-07	1.43	35	1.52173913	Snx3	
DMRX:33678101	X	33678101	200	1	8.57E-06	1.71	7	3.5	RF00407;RF00598	
DMRX:48275401	X	48275401	1100	1	7.42E-06	1.15	13	1.181818182		
DMRX:51137601	X	51137601	300	1	4.21E-06	1.13	3	1		
DMRX:53194501	X	53194501	300	1	5.17E-06	1.55	2	0.666666667	Dmd	Development
DMRX:59538101	X	59538101	1000	1	5.17E-06	1.63	2	0.2		
DMRX:88234801	X	88234801	200	1	8.32E-06	1.96	1	0.5		
DMRX:103960701	X	103960701	700	1	1.36E-07	1.47	12	1.714285714		
DMRX:112965901	X	112965901	700	1	6.24E-06	1.63	4	0.571428571	Col4a5	
