# Supplementary material for: Vinclozolin induced epigenetic transgenerational inheritance of pathologies and sperm epimutation biomarkers for specific diseases
Source: PLoS One. 2018 Aug 29;13(8):e0202662. doi: 10.1371/journal.pone.0202662 (PMC6114855; doi:10.1371/journal.pone.0202662)
Supplement: S7 Table — DMR name, chromosome, start, length, number of signature windows, minimum p-value, CpG number, CpG density, maximum log fold change, annotation, gene and functional category presented. (PDF) [file pone.0202662.s008.pdf]

Supplemental Table S7
Multiple Diseases DMR Signature List

DMR Name	Chr	Start	Length	# Sig Win	minP	Log Fold Change	CpG #	CpG Density	Gene Annotation	Gene Category
DMR1:34771501	1	34771501	400	1	7.48E-06	-1.68	4	1		
DMR1:60694901	1	60694901	3200	1	4.36E-06	-0.85	27	0.84375		
DMR1:65169901	1	65169901	1500	1	8.76E-06	-1.47	3	0.2	Vom2r80	Receptor
DMR1:71095901	1	71095901	1700	1	2.29E-06	-1.8	11	0.647058824		
DMR1:84980301	1	84980301	1100	1	1.51E-06	-1.3	9	0.818181818	Psmc4	Proteolysis
DMR1:103226201	1	103226201	300	1	9.17E-06	-1.6	3	1	Ptpn5	Signaling
DMR1:109170001	1	109170001	500	1	7.25E-06	-1.26	21	4.2		
DMR1:146048201	1	146048201	1200	1	2.04E-06	1.23	9	0.75	Mesd	
DMR1:213704801	1	213704801	2200	1	7.16E-06	-1.09	27	1.227272727		
DMR1:217156201	1	217156201	200	1	3.74E-06	1.63	2	1	Shank2;AABR07006076.	
DMR1:233819601	1	233819601	200	1	3.87E-08	-1.45	3	1.5		
DMR1:249886501	1	249886501	200	1	3.61E-06	-1.66	3	1.5		
DMR1:261855401	1	261855401	1300	1	9.35E-06	1.24	30	2.307692308	Loxl4	Receptor
DMR1:274688901	1	274688901	900	1	6.40E-06	-1.48	4	0.444444444	Shoc2	Signaling
DMR2:7489201	2	7489201	1600	1	1.20E-06	1.38	18	1.125		
DMR2:80049101	2	80049101	1200	1	8.12E-06	-1.32	14	1.166666667		
DMR2:86852601	2	86852601	1500	1	4.41E-06	-1.36	5	0.333333333		
DMR2:91240201	2	91240201	600	1	4.51E-06	-1.63	6	1		
DMR2:96152901	2	96152901	100	1	3.93E-06	-1.84	1	1		
DMR2:101997401	2	101997401	200	1	8.40E-06	-1.16	3	1.5		
DMR2:186473201	2	186473201	1100	1	9.92E-06	-1.22	18	1.636363636	Kirrel1	
DMR2:254932701	2	254932701	100	1	3.93E-06	-1.84	2	2		
DMR3:4064701	3	4064701	2000	1	4.55E-06	1	41	2.05	Agpat2	Metabolism
DMR3:23897801	3	23897801	200	1	1.08E-06	1.34	0	0		
DMR3:37871401	3	37871401	200	1	8.90E-06	1.15	2	1	AABR07052125.1	
DMR3:38273401	3	38273401	200	1	2.03E-06	-1.26	7	3.5	Stam2;AABR07052130.1	Transport
DMR3:49289201	3	49289201	200	1	9.28E-06	-1.3	1	0.5		
DMR3:51696101	3	51696101	200	1	1.98E-06	-1.3	1	0.5	AABR07052405.1;Scn2a	
DMR3:61803101	3	61803101	1500	1	4.33E-06	-1.13	5	0.333333333	Mtx2	Binding Protein
DMR3:62471101	3	62471101	1100	1	8.31E-06	-1.68	14	1.272727273	AC109877.1;Hnrnpa3	Translation
DMR3:65055201	3	65055201	300	1	7.93E-06	-0.82	4	1.333333333		
DMR3:79979601	3	79979601	200	1	3.90E-06	1.04	2	1	Madd	Signaling
DMR3:81556401	3	81556401	100	1	5.83E-07	2.59	0	0		
DMR3:93232401	3	93232401	800	1	7.92E-06	1.69	11	1.375		
DMR3:116456101	3	116456101	900	1	1.98E-06	-1.69	14	1.555555556		
DMR3:142164001	3	142164001	300	1	1.98E-06	1.47	3	1		
DMR3:148059101	3	148059101	300	1	7.56E-06	1.09	4	1.333333333	Defb24;AC111428.2	Signaling
DMR3:149518401	3	149518401	400	1	5.99E-06	-1.41	2	0.5	Bpifa2f	
DMR3:153947101	3	153947101	300	1	2.99E-06	2.01	4	1.333333333	LOC100911217	
DMR3:163433701	3	163433701	1400	1	6.81E-06	1.34	22	1.571428571	Prex1	
DMR3:166129901	3	166129901	200	1	4.22E-06	1.57	3	1.5		
DMR4:50554801	4	50554801	200	1	3.15E-07	-1.87	1	0.5	Cadps2	Metabolism
DMR4:60639001	4	60639001	300	1	1.25E-06	-1.15	4	1.333333333	Exoc4	Transport
DMR4:88161601	4	88161601	500	1	9.02E-06	-0.51	9	1.8		
DMR4:91503901	4	91503901	500	1	1.67E-07	-1.9	6	1.2	Ccser1	
DMR4:91722401	4	91722401	200	1	5.00E-06	-1.55	2	1	Ccser1	
DMR4:102359001	4	102359001	200	1	3.93E-06	-1.84	1	0.5	AABR07060994.1	
DMR4:124370001	4	124370001	1400	1	8.81E-06	-1.56	16	1.142857143		
DMR4:132891201	4	132891201	2200	1	1.44E-06	1.29	27	1.227272727		
DMR4:134881101	4	134881101	1000	1	8.96E-06	-1.68	7	0.7	Cntn3	Cytoskeleton
DMR4:146418001	4	146418001	100	1	7.20E-06	-1.74	0	0		
DMR4:154364601	4	154364601	1100	1	7.59E-06	1.05	14	1.272727273	LOC100911545	Immune response
DMR4:155195601	4	155195601	200	1	1.22E-06	2.39	0	0		
DMR4:159624101	4	159624101	500	1	1.11E-06	1.43	3	0.6	Fgf23	Signaling
DMR4:167864901	4	167864901	3300	1	7.78E-06	-1.69	64	1.939393939	Etv6	Transcription
DMR5:1909301	5	1909301	1200	1	2.08E-06	-2.11	9	0.75		
DMR5:6461001	5	6461001	1500	1	2.39E-07	-2.01	20	1.333333333		
DMR5:25958101	5	25958101	300	1	2.07E-07	-1.1	8	2.666666667		

DMR5:48247701	5	48247701	1200	1	1.90E-06	-1.42	28	2.333333333	Rragd	Signaling
DMR5:51854001	5	51854001	200	1	4.84E-06	1.29	0	0		
DMR5:66422701	5	66422701	200	2	3.28E-06	2.1	1	0.5		
DMR5:100690401	5	100690401	500	1	2.61E-06	-1.61	4	0.8		
DMR5:105041901	5	105041901	400	1	4.35E-06	-1.69	4	1		
DMR5:107726001	5	107726001	300	1	2.99E-06	-1.05	7	2.333333333	Mtap	
DMR5:122381001	5	122381001	1500	1	8.74E-06	2.08	18	1.2	Sgip1	
DMR5:149515901	5	149515901	100	1	6.70E-06	-1.91	2	2		
DMR5:173548001	5	173548001	600	1	8.12E-06	-1.32	8	1.333333333	RGD1311517	
DMR6:11815201	6	11815201	300	1	2.89E-06	1.27	3	1		
DMR6:24283601	6	24283601	200	1	9.99E-06	-1.43	4	2	AABR07063250.1	
DMR6:24667401	6	24667401	2400	1	9.97E-06	-1.5	28	1.166666667	Capn13	Protease
DMR6:42059501	6	42059501	300	1	2.84E-06	-1.33	5	1.666666667		
DMR6:52647401	6	52647401	900	1	1.08E-07	2.85	29	3.222222222	Atxn7l1	Development
DMR6:60810901	6	60810901	200	1	4.30E-06	-1.6	3	1.5		
DMR6:63512101	6	63512101	2200	1	9.79E-07	-1.52	23	1.045454545	RF00026	
DMR6:66809001	6	66809001	100	1	4.48E-06	3.38	0	0		
DMR6:107610401	6	107610401	200	1	3.83E-07	1.28	2	1	Dnal1	Cytoskeleton
DMR6:115563801	6	115563801	200	1	3.46E-06	1.32	2	1	Sel1l	Signaling
DMR6:133902201	6	133902201	300	1	1.62E-06	-1.61	9	3	Mir541;Mir3581;Mir412;Mir3578;Mir410;Mir307	
DMR6:136004401	6	136004401	100	1	2.75E-06	-1.25	5	5	AABR07065593.1;Eif5;RF00400	Transcription
DMR7:2848801	7	2848801	700	1	9.51E-06	-1.23	27	3.857142857	Rnf41	
DMR7:3366301	7	3366301	300	1	8.12E-06	-1.26	12	4	Itga7	Signaling
DMR7:10726601	7	10726601	700	1	7.04E-06	-1.29	3	0.428571429		
DMR7:12343701	7	12343701	500	1	3.43E-06	-1.33	11	2.2	Mum1	Transcription
DMR7:34116701	7	34116701	1100	1	8.06E-06	0.72	29	2.636363636	Elk3	Transcription
DMR7:51597201	7	51597201	400	1	1.87E-06	-1.2	3	0.75		
DMR7:51967301	7	51967301	2300	1	2.76E-06	-0.77	20	0.869565217		
DMR7:68086101	7	68086101	1000	1	2.26E-06	-1.64	1	0.1		
DMR7:69279901	7	69279901	1400	1	1.12E-06	-1.38	17	1.214285714		
DMR7:77662301	7	77662301	1300	1	5.84E-06	-1.35	7	0.538461538	RF00001	
DMR7:87975401	7	87975401	400	2	1.25E-06	3.12	5	1.25	AABR07057765.1	
DMR7:115227401	7	115227401	200	1	8.13E-06	-1.18	1	0.5		
DMR7:124880201	7	124880201	1300	1	7.87E-06	0.74	16	1.230769231	Efcab6	Signaling
DMR7:136139201	7	136139201	600	1	7.37E-06	-1.43	17	2.833333333		
DMR7:141274601	7	141274601	1100	1	1.26E-06	-1.36	26	2.363636364	Racgap1	Binding Protein
DMR8:4018001	8	4018001	300	1	8.90E-06	1.41	2	0.666666667	Vom2r23	
DMR8:30326401	8	30326401	1200	1	2.67E-06	2.23	8	0.666666667		
DMR8:31147901	8	31147901	200	1	5.92E-06	2.45	0	0		
DMR8:52024701	8	52024701	1200	1	9.97E-06	-1.5	11	0.916666667		
DMR8:70311701	8	70311701	300	1	4.36E-06	-1.26	3	1	Dennd4a	
DMR8:114180801	8	114180801	300	1	2.64E-06	-1.14	8	2.666666667	LOC102552009;LOC102552166	
DMR8:119164201	8	119164201	400	1	2.93E-06	0.7	0	0	Tmie;Als2cl	
DMR8:127940901	8	127940901	1300	1	9.97E-06	-1.98	12	0.923076923	Oxsr1	Signaling
DMR9:18700601	9	18700601	300	1	3.68E-06	1.88	3	1	Runx2	Transcription
DMR9:19408201	9	19408201	300	1	6.33E-06	-0.81	6	2		
DMR9:32238601	9	32238601	2000	1	8.34E-06	-1.39	11	0.55		
DMR9:41923401	9	41923401	200	1	3.23E-06	-1.33	4	2		
DMR9:58221501	9	58221501	200	1	9.10E-07	-1.02	1	0.5		
DMR9:62162401	9	62162401	800	1	3.25E-06	-1.8	17	2.125		
DMR9:78715201	9	78715201	2100	1	2.41E-07	-0.74	12	0.571428571		
DMR9:91680501	9	91680501	100	1	4.29E-06	-1.73	0	0	Pid1	Signaling
DMR9:110373801	9	110373801	300	1	3.90E-07	-0.9	3	1		
DMR10:31415501	10	31415501	300	1	5.70E-06	2.98	5	1.666666667	Cyfip2	Development
DMR10:44409201	10	44409201	200	1	3.93E-06	-1.84	0	0	Olr1437	Receptor
DMR10:51189801	10	51189801	200	1	7.25E-06	1.4	1	0.5		
DMR10:81617801	10	81617801	300	1	6.47E-06	4.12	0	0	Mbtd1	Transcription
DMR11:20313601	11	20313601	1100	1	1.63E-06	1.42	7	0.636363636		

DMR11:46737901	11	46737901	300	1	2.84E-06	-1.67	2	0.666666667	Tmem45al;AABR07033979.1	
DMR11:48296201	11	48296201	100	1	4.30E-07	3.602	0	0		
DMR11:49442501	11	49442501	300	1	6.30E-06	1.09	1	0.333333333		
DMR12:31501	12	31501	200	1	8.49E-07	-1.33	8	4		
DMR12:1756401	12	1756401	800	1	8.45E-06	2.09	15	1.875	Insr	Receptor
DMR12:8885801	12	8885801	300	1	1.60E-06	1.86	8	2.666666667		
DMR12:11146001	12	11146001	2300	1	9.17E-06	0.57	17	0.739130435	Zfp655;Zkscan5	Transcription
DMR12:14506901	12	14506901	600	1	1.22E-06	2.82	4	0.666666667	AABR07035437.1	
DMR12:16362701	12	16362701	200	1	8.83E-06	-1.1	4	2	Snx8	Signaling
DMR13:24299101	13	24299101	900	1	8.22E-06	1.7	7	0.777777778		
DMR13:39166701	13	39166701	700	1	8.04E-06	-1.89	3	0.428571429		
DMR13:50169801	13	50169801	300	1	8.68E-06	-1.56	3	1	Lax1	
DMR13:87863701	13	87863701	800	1	5.97E-06	-0.86	6	0.75		
DMR13:89494501	13	89494501	1200	1	8.98E-06	-1.01	25	2.083333333	Cfap126;Sdhc	
DMR13:109752501	13	109752501	300	1	1.19E-06	1.33	3	1		
DMR14:16862901	14	16862901	700	1	6.35E-06	-1.42	4	0.571428571	Shroom3	Cytoskeleton
DMR14:30911701	14	30911701	1400	1	8.34E-06	-1.32	5	0.357142857		
DMR14:35522201	14	35522201	1900	1	2.91E-06	3.04	30	1.578947368	Pdgfra	Growth factor
DMR14:36027901	14	36027901	1300	1	5.28E-06	-1.43	13	1		
DMR14:83632401	14	83632401	300	1	9.41E-06	-0.98	8	2.666666667	Limk2	Signaling
DMR14:86964501	14	86964501	200	1	1.53E-06	1.41	3	1.5	RF00015	
DMR14:113842801	14	113842801	300	1	8.72E-06	-0.92	1	0.333333333		
DMR15:18396001	15	18396001	200	1	2.16E-06	1.45	1	0.5	Fam107a	
DMR15:18774001	15	18774001	700	1	3.30E-07	-1.33	12	1.714285714	Flnb	Cytoskeleton
DMR15:20066101	15	20066101	1200	1	1.25E-06	3.03	24	2		
DMR15:30134901	15	30134901	1100	1	5.55E-06	2.37	2	0.181818182		
DMR15:30786001	15	30786001	1200	1	7.24E-06	-1.61	7	0.583333333	AABR07017768.5;AABR07017768.6	
DMR15:56262401	15	56262401	300	1	5.63E-06	-1.22	0	0		
DMR15:61428501	15	61428501	2500	1	4.63E-06	-0.93	39	1.56		
DMR15:88240001	15	88240001	300	1	4.25E-06	-1.29	4	1.333333333	Mycbp2	Metabolism
DMR15:94720601	15	94720601	100	1	9.25E-06	-1.65	4	4		
DMR15:105161501	15	105161501	1300	1	1.00E-06	-1.28	32	2.461538462	Hs6st3	Metabolism
DMR16:20361201	16	20361201	600	1	3.06E-06	1.44	9	1.5	Arrdc2;AABR07024875.1;Il12rb1	
DMR16:28615301	16	28615301	300	1	1.38E-06	-0.93	3	1	Spock3	Signaling
DMR16:52425201	16	52425201	400	1	5.84E-06	1.14	2	0.5		
DMR16:67750401	16	67750401	1200	1	1.45E-06	-1.83	21	1.75		
DMR16:80495201	16	80495201	1400	1	2.61E-06	-1.76	23	1.642857143		
DMR16:81133501	16	81133501	400	1	8.29E-06	-1.28	4	1	Tfdp1;Atp4b	Transcription;Transport
DMR17:513001	17	513001	400	1	5.05E-06	-0.81	4	1	Npepo	Proteolysis
DMR17:6471601	17	6471601	1900	1	4.43E-06	3.01	27	1.421052632	Slc28a3	Metabolism
DMR17:19045601	17	19045601	1100	1	9.17E-06	-1.54	10	0.909090909	LOC102546683	
DMR17:27473001	17	27473001	600	1	8.19E-07	2	4	0.666666667	Cage1	
DMR17:50255201	17	50255201	2100	1	2.08E-06	-0.85	18	0.857142857		
DMR17:74170301	17	74170301	600	1	8.12E-06	0.86	6	1		
DMR18:3762101	18	3762101	1100	1	1.32E-06	-1.1	24	2.181818182	AABR07031193.1	
DMR18:6091701	18	6091701	300	1	1.28E-06	-0.97	1	0.333333333	Ss18	Transcription
DMR18:40104001	18	40104001	300	1	1.26E-06	-1.41	3	1	Trim36	Metabolism
DMR18:49880901	18	49880901	300	1	1.35E-06	-1.41	7	2.333333333	Zfp608	
DMR18:85107201	18	85107201	300	1	2.19E-06	-1.66	4	1.333333333		
DMR19:21842201	19	21842201	300	1	3.06E-06	-1.24	6	2		
DMR19:38647901	19	38647901	200	1	9.93E-06	1.28	3	1.5	LOC100360619	
DMR20:12220301	20	12220301	1100	1	8.90E-06	-1.94	5	0.454545455	Col18a1	Extracellular Matrix
DMR20:17828801	20	17828801	300	1	2.08E-07	-1.41	4	1.333333333		
DMR20:26654001	20	26654001	300	1	3.33E-06	0.92	3	1		
DMR20:39945301	20	39945301	300	1	2.11E-06	0.7	0	0		
DMR20:48263101	20	48263101	2200	1	8.47E-06	-1.24	36	1.636363636	Bend3	
DMRX:119142201	X	119142201	300	1	4.10E-06	1.92	1	0.333333333		
DMRX:135671701	X	135671701	500	1	2.26E-06	-1.49	4	0.8		
DMRX:143053801	X	143053801	300	1	9.20E-06	-1.43	1	0.333333333		
